# Two Novel Dinuclear Cobalt Polypyridyl Complexes in Electro‐ and Photocatalysis for Hydrogen Production: Cooperativity Increases Performance

**DOI:** 10.1002/cssc.202201049

**Published:** 2022-07-21

**Authors:** Nicola Weder, Nora S. Grundmann, Benjamin Probst, Olivier Blacque, Rangsiman Ketkaew, Fabrizio Creazzo, Sandra Luber, Roger Alberto

**Affiliations:** ^1^ Department of Chemistry University of Zurich Winterthurerstrasse 190 Switzerland

**Keywords:** artificial photosynthesis, electrocatalysis, hydrogen, solar fuels, water reduction

## Abstract

Syntheses and mechanisms of two dinuclear Co‐polypyridyl catalysts for the H_2_ evolution reaction (HER) were reported and compared to their mononuclear analogue (**R1**). In both catalysts, two di‐(2,2’‐bipyridin‐6‐yl)‐methanone units were linked by either 2,2’‐bipyridin‐6,6’‐yl or pyrazin‐2,5‐yl. Complexation with Co^II^ gave dinuclear compounds bridged by pyrazine (**C2**) or bipyridine (**C1**). Photocatalytic HER gave turnover numbers (TONs) of up to 20000 (**C2**) and 7000 (**C1**) in water. Electrochemically, **C1** was similar to the **R1**, whereas **C2** showed electronic coupling between the two Co centers. The *E*(Co^II/I^) split by 360 mV into two separate waves. Proton reduction in DMF was investigated for **R1** with [HNEt_3_](BF_4_) by simulation, foot of the wave analysis, and linear sweep voltammetry (LSV) with in‐line detection of H_2_. All methods agreed well with an (E)ECEC mechanism and the first protonation being rate limiting (≈10^4^ m ^−1^ s^−1^). The second reduction was more anodic than the first one. p*K*_a_ values of around 10 and 7.5 were found for the two protonations. LSV analysis with H_2_ detection for all catalysts and acids with different p*K*_a_ values [HBF_4_, p*K*_a_(DMF)≈3.4], intermediate {[HNEt_3_](BF_4_), p*K*_a_(DMF)≈9.2} to weak [AcOH, p*K*_a_(DMF)≈13.5] confirmed electrochemical H_2_ production, distinctly dependent on the p*K*_a_ values. Only HBF_4_ protonated Co^I^ intermediates. The two metals in the dualcore **C2** cooperated with an increase in rate to a competitive 10^5^ m ^−1^ s^−1^ with [HNEt_3_](BF_4_). The overpotential decreased compared to **R1** by 100 mV. Chronoamperometry established high stabilities for all catalysts with TON_lim_ of 100 for **R1** and 320 for **C1** and **C2**.

## Graphical abstract

**Take two**: Electrochemical investigations of different dinuclear cobalt‐polypyridyl water reduction catalysts reveal the effect of electrochemical coupling of both metal cores on the reduction mechanism.

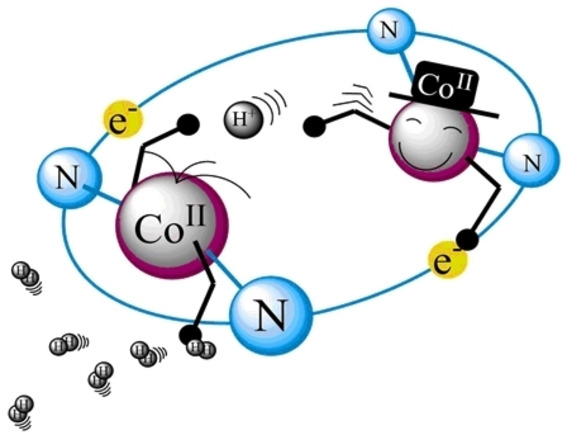

## Introduction

In photocatalytic water splitting, cobalt polypyridyl complexes are potent catalysts for proton reduction.[^1^, ^2^, ^3^, ^4^, ^5^, ^6^, ^7^, ^8^] While exhibiting higher stabilities compared to other molecular water reduction catalysts (WRCs),^[9]^ they display higher overpotentials decreasing the efficiency of light‐induced proton reduction. Due to the low basicity of pyridyl‐coordinated cobalt, several subsequent metal‐ and ligand‐based reductions are required, preceding the first protonation, except when very strong acids are applied.[^1^, ^4^, ^10^, ^11^]

Similar issues have been intensively addressed in water oxidation, where four subsequent hole transfers lead to charge accumulation, which causes high overpotentials. Inspired by the (Mn)_4_‐core of photosystem II,[^12^, ^13^] sequential charge accumulation on multinuclear complexes, lowering the overall onset potential, has been subject of numerous studies on photocatalytic water oxidation.[^14^, ^15^, ^16^, ^17^] On the reductive side, multinuclear cobalt complexes are reported in literature; however, the studies are mostly focused on structural and spectroscopic investigations or magnetic properties.[^18^, ^19^, ^20^, ^21^, ^22^, ^23^] Investigations of water reducing properties of these complexes are scarce.[^24^, ^25^]

We examine the synthesis and water‐reduction mechanisms of two new, dinuclear cobalt polypyridyl WRCs, structurally based on a mononuclear complex previously reported by our group.^[26]^ Both compounds comprise two di‐(2,2’‐bipyridin‐6‐yl)‐methanolyl units bridged via either bipyridine (**C1**) or pyrazine (**C2**). The previously reported compound **R1** is the mononuclear analogue and serves as reference.

## Results and Discussion

**Figure 1:**
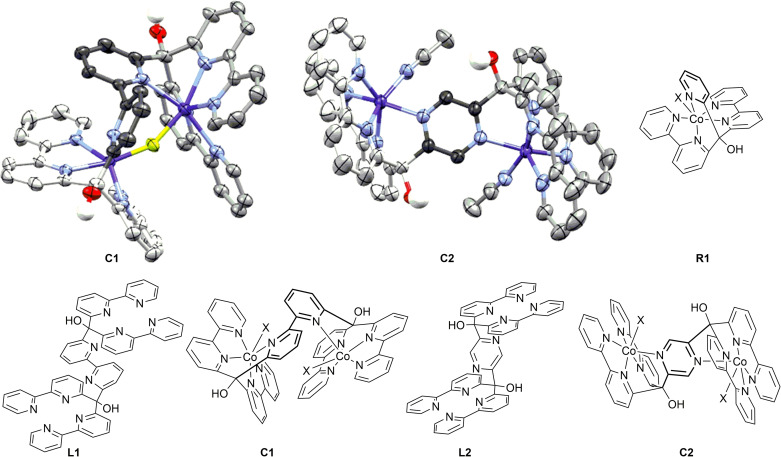
X‐ray structure of dual‐cores **C1** (left, X=μ‐F) and **C2** (right, X=MeCN) and molecular structures of the ligands and complexes. Thermal ellipsoids are at the 50 % probability level, selected hydrogen atoms, BF_4_^−^ counter ions, Br^−^, and solvent molecules are omitted for clarity. Color code: grey (C), white (H), red (O), blue (N), yellow (F), purple (Co).

The ligands **L1** and **L2** were synthesized by double‐lithiation of 6,6’‐dibromobipyridine or 2,5‐dibromopyrazine, respectively, and subsequent nucleophilic addition to di‐(2,2’‐bipyridin‐6‐yl)‐methanone (ESI).^[4]^ The final compounds **C1** and **C2** were obtained after addition of excess Co(BF_4_)_2_ to the respective ligand. Excess metal was removed by THF trituration. Crystals suitable for X‐ray crystallography of both complexes were obtained by the vapor diffusion method from MeCN with CHCl_3_ as anti‐solvent.^[27]^ The crystal structures are shown in Figure 1, and crystallographic data are given in Tables S1 and S2. Common structural features comprise the cobalt atoms in the centers of both di‐(bipyridyl)‐units, each additionally coordinated by the bridging moiety, connecting both metal centers. The sixth coordination site per cobalt is occupied by a bridging fluoride or solvent molecules, respectively, the former one being formed by fluoride abstraction from the counter ion (BF_4_)^−^.[^28^, ^29^, ^30^, ^31^, ^32^] Two distinct differences between both structures are notable: the “embracing” geometry of the ligand in **C1** forms an envelope‐shaped structure around both metal centers bringing them in close proximity (Figure 1). This strained configuration results in a twisted geometry of the bridging bipyridine unit, making electronic coupling via the ligand between the two metal centers unlikely. The distorted geometry is already anticipated in the crystal structure of the uncoordinated ligand **L1**, exhibiting a twisted bipyridyl‐bridge connecting both di‐(2,2’‐bipyridin‐6‐yl)‐methanol units (ESI). Complex **C2** shows a Z‐like structure, positioning the cobalt atoms in larger distances from each other on both sides of the pyrazine unit but enabling electronic coupling of the cobalt‐centers via the pyrazyl moiety.

**Figure 2:**
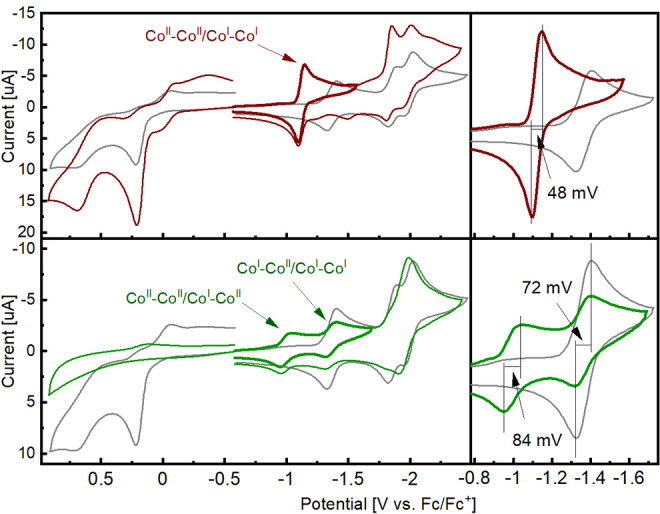
CVs of **C1** (top; 1 mm) and **C2** (bottom; 0.55 mm) recorded in DMF (0.1 m TBAPF_6_) at a glassy carbon electrode and 100 mV s^−1^. The grey dotted lines belong to **R1** (1 mm).

**Figure 3:**
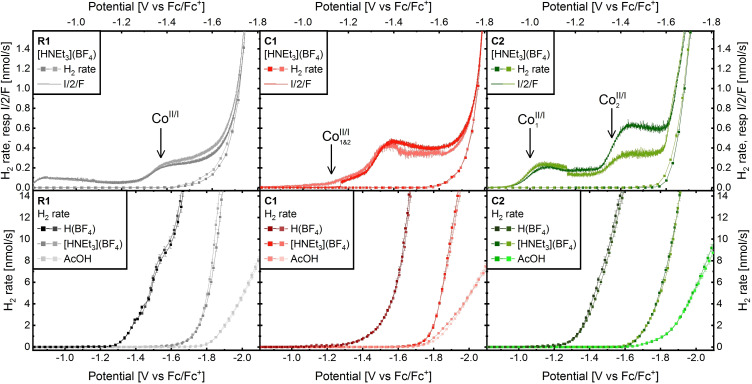
Results of LSV for **R1** (left), **C1** (middle), and **C2** (right) with additional inline monitoring of H_2_ production by gas chromatography. Top: H_2_ traces and converted current (**I/2/F**) recorded with 500 equiv. of [HNEt_3_](BF_4_) as proton source. Bottom: H_2_ traces recorded with AcOH, [HNEt_3_](BF_4_), and H(BF_4_), respectively. All data collected on an Hg pool (electrode area *S*=1.3±0.1 cm^2^), in DMF, 0.1 m TBAPF_6_, 0.4 mm catalyst, 200 mm proton source, scan rate 0.1 mV s^−1^, *R*_u_≈70 Ω, *T*=300 K.

**Figure 4:**
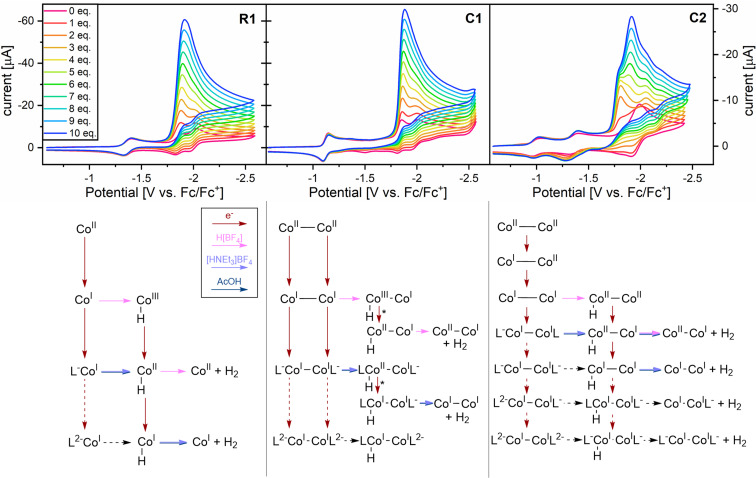
Top: CVs of **R1** (left), **C1** (middle, both 1 mm), and **C2** (right, 0.55 mm) recorded in DMF, 0.1 m [TBA](PF_6_) and equivalent‐wise addition of [HNEt_3_](BF_4_) (pink: 0 equiv. to blue: 10 equiv., in 1 equiv. steps) at a glassy carbon electrode and 0.1 V s^−1^. Bottom: Mechanistic scheme of electron (red arrows, vertical) and proton transfers (pink, blue, and navy arrows, horizontal) of **R1** (left), **C1**, (middle), and **C2** (right). Dashed arrows indicate alternative pathways relevant at more cathodic potentials. The oxidation numbers on cobalt are formal notations, assuming a simplistic polarization of the Co−H bond as a hydride. The electron transfer step for protonated **C1**, denoted with *, is displayed as electrode electron transfer but could as well be a homogenous electron transfer.

Cyclic voltammograms (CVs) of **C1**, **C2**, and **R1** were recorded in DMF with 0.1 m [N(Bu)_4_](PF_6_) ([TBA](PF_6_)) as electrolyte (Figure 2) and [HNEt_3_](BF_4_) as proton source (Figure 4). Linear sweep voltammograms (LSV) with in line H_2_ detection were recorded in the same electrolyte with either AcOH (acetic acid), [HNEt_3_](BF_4_), or H(BF_4_) as proton source (Figure 3; p*K*_a_ values in DMF are 13.5, 9.2, and 3.4).[^33^, ^34^] Glacial AcOH and [HNEt_3_](BF_4_) were added neat, H(BF_4_) was added as [HEt_2_O](BF_4_). Both di‐cobalt complexes are present as tetra‐(BF_4_)^−^ salts, with **C2** being in its crystalline form.

The region between −0.8 and −1.6 V, where the Co^II/I^ reduction is expected for polypyridyl complexes, is magnified on the right side of Figure 2. For **R1** there are three reversible reductions (grey line) at −1.37, −1.85, and −2.00 V. The first reduction is tentatively assigned to a formal reduction of Co^II^ to Co^I^, in line with our previous reports and density functional theory (DFT) calculations (see the Supporting Information for computational details).[^35^, ^36^, ^37^] At lower potentials (−1.75 to −2.25 V) redox processes are often ligand‐based. For fully conjugated ligand systems (such as cyano‐methylene bridged bipyridyls), one single reduction was reported in this region, whereas electronically separated moieties within the ligand, as in **R1**, **C1**, and **C2**, lead to several subsequent reductions.^[35]^ Analyses of the CVs of **R1** with the software package DigiElch[^38^, ^39^, ^40^, ^41^, ^42^, ^43^, ^44^, ^45^, ^46^, ^47^] at varying scan rates confirm these potentials, and gives a diffusion coefficient of 2.8×10^−6^ cm^2^ s^−1^ (Figure SI5 and Table SI3), in line with literature reports for similar compounds.[^10^, ^48^, ^49^]

The bipyridyl‐bridged **C1** (red trace) exhibits one reversible redox peak with a half wave potential of *E*_1/2_=−1.13 V, followed by two broader peaks at −1.84 and −1.98 V. Comparison with **R1** reveals an anodic shift for the first reduction by 245 mV, a significantly smaller peak separation of 48 mV (as compared to 71 mV for **R1**), and an approximately doubled peak current. The latter two are clear indications for a two‐electron reduction in **C1**, as opposed to a one‐electron process in **R1**. Simulation of the CVs indeed revealed two overlying reversible redox processes with half wave potentials of at −1.12 and −1.13 V. At potentials more negative, **C1** exhibits two reductive waves, very close to the half‐wave potentials of the respective peaks of **R1** but again peaking at twice the current. A simulation with four redox couples at −1.83, −1.84, −1.95, and −2.01 V reproduces the shape very well, with an increased spacing of the last two reductions leading to a broader shape of the second wave (see Figure SI6 and Table SI4). These events are consequently attributed to subsequent reductions of the bipyridyl subunits (bipy/bipy^−^), as both **R1** and **C1** carry those moieties, but **C1** contains twice as many. The very close spacing of all 6 reductions to three pairs in **C1** suggests significant electronic separation of the two halves. Electrochemical communication would effect a dependence of the reduction potential of one cobalt on the oxidation state of its counterpart. The same is valid for the ligand‐based reductions. However, in both cases, the “mirrored” units are electrochemically almost indistinguishable. We thus hypothesize that the twisted configuration of the bridging bipyridyl‐unit interrupts the mesomeric structure and prevents electrochemical communication between both cobalt centers. To confirm this hypothesis, we calculated population charges and electron transfer coupling using (constrained) DFT to quantify intramolecular electronic communication of **C1** and **C2** (see section 6 for computational details). Quite interestingly, our calculations reveal a stable geometry of the complex in the proposed twisted form.

The CVs of the pyrazine‐bridged dual‐core **C2** demonstrate novel features, as shown in Figure 2 (bottom). The enlarged potential window displays two clearly distinguishable reductions at −1.0 and −1.36 V. The peak separations of both events match a one‐electron transfer, and the concentration‐corrected peak current is equal to the reference **R1**. This suggests two subsequent one‐electron reductions at both cobalt centers (Co^II^−Co^II^ to Co^I^−Co^II^ to Co^I^−Co^I^, see also Figure 4, right bottom). Consequently, the two metal centers are electronically coupled and the reduction potential of one cobalt depends on the oxidation state of the other. In accordance with the crystal structure, electronic coupling occurs via the bridging pyrazyl moiety, coordinating to both cobalt atoms via the conjugated 6‐membered ring.^[50]^ The next reductions, with a broad peak at around −1.96 V, likely consist of four overlapping, ligand based redox processes, in analogy to **R1** and **C1**. Simulations of these processes with DigiElch gave an estimate of −1.83, −1.91, −1.96, and −2.00 V vs. Fc/Fc^+^ (Figure SI7 and Table SI5).

The potentials of the first reductions in both **C1** and **C2** are anodically shifted as compared to **R1**. This is in line with the higher overall charge of the dual‐cores **C1** and **C2**. Moreover, this is consistent with earlier results showing less negative *E*_1/2_(Co^II/I^) for larger conjugated systems in the ligand, for example, hexapyridyl>pentapyridyl>tetrapyridyl^[26]^ or in pyridazyl‐bridged cobalt dual‐cores.[^18^, ^24^] An increased number of α‐imines (pyrazine>pyridine) on the ligand exhibits stronger π‐backbonding from the metal, thereby stabilizing the Co^I^ state and shifting the potential in an anodic direction.^[51]^

Insights to the proton reduction mechanism is gained upon incremental addition of the H^+^ source [HNEt_3_](BF_4_) by careful analysis of both, the CVs (Figure 4) and the LSVs with in‐line H_2_ detection (Figure 3). The upper part of Figure 4 shows CVs after addition of 0–10 proton‐equivalents to a solution of **R1**, **C1**, and **C2**, respectively (left to right) in DMF. The corresponding mechanisms are illustrated in the lower part of Figure 4. Reductions are vertically arranged red arrows; protonation steps correspond to horizontally aligned pink, blue, or navy arrows (for H(BF_4_), [HNEt_3_](BF_4_), or AcOH).

Paralleling (E)ECEC and (E)EECC pathways were extracted in the case of **R1** upon addition of [HNEt_3_](BF_4_), by simulation and fitting in DigiElch (Figure SI8), as depicted in the lower panel of Figure 4. Protonation thus occurs on both, [L^−^−Co^I^] and [L^2−^−Co^I^], in the former case with a p*K*_a_ of around 10, and a rate constant of 1.3×10^4^ m ^−1^ s^−1^; in the latter case, however, the p*K*_a_ is coupled to the reduction potential *E*([Co^II/I^−H]). Assuming a realistic value of −1.8 V for the latter reduction, a p*K*_a_ of around 13.5 is obtained for protonation of [L^2−^−Co^I^] (see Table SI6). As for the second, final protonation step of [L−Co^I^−H], a p*K*_a_ of around 7.5 is obtained. A foot of the wave analysis (FOWA) of the CV data can only be used to confirm the (E)ECEC pathway, operational at less negative potentials, since the (E)EECC pathway is obscured by the former.[^52^, ^53^, ^54^, ^55^, ^56^] The catalytic half wave potential is constant at −1.84±0.02 V, and independent on the acid concentration (Figures SI9–SI17). Moreover, the first reduction remains reversible upon acid addition. This excludes a homolytic pathway, or a second, more negative reduction than the first one, indicating that the second step is faster than the first one. No further distinction between the remaining options (E)ECEC, EECC and an (E)ECCE, or their analogues with one homogeneous electron transfer, (E)ECE'C, EE'CC or (E)ECCE’, is possible by a FOWA, since the same dependencies are expected. In any of these cases, the second protonation must be significantly faster than the first one. A rate constant for the slower first protonation of 5.3×10^3^ m ^−1^ s^−1^ is obtained (Table SI7), which is reasonably close to the 1.3×10^4^ m ^−1^ s^−1^ obtained from the full simulation described before. By simulation, the seemingly simpler EECC mechanism does not reproduce the increasing irreversibility of the first catalytic wave as well as the (E)ECEC mechanism. The same is true for the (E)ECCE mechanism; the simulation reveals a deviation in the shape of the reverse wave. Albeit not accessible by FOWA only, simulation thus favors the (E)ECEC (or (E)ECE'C) mechanisms. Further confirmation of the ECEC sequence comes from the fact that the first chemical step is significantly slower than the second one is. It seems unlikely that double protonation of one species (successive CC steps) becomes faster in the second step.

**Figure 5:**
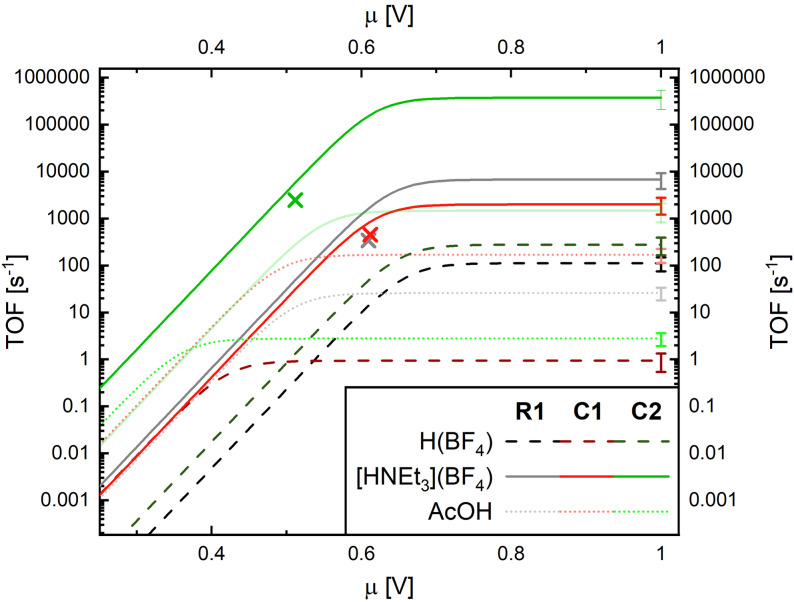
Catalytic Tafel plots for **R1** (grey), **C1** (red), and **C2** (green) with H(BF_4_), [HNEt_3_](BF_4_), and AcOH. The asterisks represent the CA experiments. The solid faint green line represents the (E)EECC mechanism for **C2** and [HNEt_3_](BF_4_).

LSV data with inline probing for H_2_ can be used to shed some more light on these findings. First, H_2_ production is confirmed at the respective potentials, and balancing the spent electrons with detected hydrogen by coulometric analysis reveals one “excess” electron (Figures SI19–SI21), corresponding to a one‐electron reduced Co^I^ as the resting state during catalysis. Moreover, also LSV data can be subjected to FOWA analysis, adding the asset that product formation rate can be analyzed with respect to the transformed potential {1+exp[F/RT(*E*−*E*_cat/2_)]}^−1^ instead of the current, which is often obscured by pre‐catalytic reductions. In line with simulations in DigiElch and FOWA of catalytic CVs, the rate of the first protonation in a (E)ECEC mechanism is determined to be 6.8±2.5×10^3^ m ^−1^ s^−1^ this way (see Figure 5 and Table SI8).

The catalytic CVs of the bipyridyl‐bridged dual‐core **C1** and the reference **R1** are highly compatible (see Figure 4 and discussion of redox events above). For identical conditions, DMF, [HNEt_3_](BF_4_), the (E)ECEC mechanisms found for **R1** would translates to an (EE)ECEC mechanism for **C1**. The simultaneous reduction of both Co^II^‐cores to [L−Co^I^−Co^I^−L] at −1.13 V is confirmed by LSV data. One subsequent ligand‐based reduction initiates the catalytic wave, and two excess electrons are found on the resting state of the **C1** catalyst during catalysis with [HNEt_3_](BF_4_) (see Figure 3 and Figures SI19–SI21). A catalytic half wave potential *E*_cat/2_ of −1.82±0.01 V, independent of acid concentration, is found by CV (Figure SI9), matching the potential of the first ligand‐based reduction at −1.83 V. This indicates a second protonation faster than the first one. A second order rate constant of 2.1±0.1×10^3^ m ^−1^ s^−1^ is extracted for the first, rate‐limiting protonation (Table SI7, Figures SI9 and SI10). The similarity of the CV at all potentials in the presence of acid (Figure 4) suggest furthermore an equally fast catalytic turnover as for **R1**, also after the second set of ligand reductions at −1.95 to −2.01 V [(EE)EECC pathway]. **C1** eludes itself from a full simulation with for example DigiElch, due to the high number of free parameters required in the simulation of catalysis on top of a 6‐electron reduction. As compared to **R1**, a slightly lower rate constant for the first step in the ECEC pathway, an equal current magnitude in the EECC domain, and the absence of a shift of *E*_cat/2_ to more anodic potentials all indicate a lack of cooperativity between the two cobalt centers in **C1**. This coincides with the electronic isolation of the two centers mentioned before; the two cores operate independently of one another, which in sum reduces the overall turnover frequency (TOF) per cobalt.

The electrochemical response of **C2** upon [HNEt_3_](BF_4_) addition is distinctly different. The two first reductions remain unchanged (Figure 4, top right) and their characters as two one‐electron transfers is further supported by two distinct events in the LSVs at those potentials (see Figure 3, top right). The shape of the next four reductions present in the absence of protons (−1.83, −1.91, −1.96, and −2.00 V), changes significantly upon addition of [HNEt_3_](BF_4_): a process with a *E*_cat/2_ of −1.76 V becomes apparent (Figure top right, Figure SI9; most pronounced at lower acid equivalents). Comparison with LSV scans (500 equiv. of protons) confirms this new peak, suggesting, however, that H_2_ evolution starts at slightly more cathodic potentials (Figure 3). Integration gives three excess electrons on **C2** during electrocatalysis (Figure SI19–21). The peak shift indicates a faster first (*k*_1_), and a slower second (*k*_2_) chemical step, according to Equation 1:

(1) $$E_{cat/2}-E_{1/2}=\frac{RT}{2F}\log\frac{k_{1}}{k_{2}}$$

Rates of 1.5±0.1×10^5^ m ^−1^ s^−1^ for the first and 8.2±0.4×10^2^ m ^−1^ s^−1^ for the second protonation are obtained from FOWA analysis of CV data (Figures SI14–SI17, Table SI7). An (E)EECC mechanism with a fast first protonation and a slower second protonation leading to release of H_2_ is compatible with these observations. Due to the fast first protonation, Co^I^−Co^II^−H is the resting state of the catalyst during catalysis, in line with the coulometric analysis (Figures SI19–SI21). As subsequent reductions of the complex take place, alternative mechanisms, for example (EE)ECEC, and later, as noted for **R1** and **C1**, (EE)EECC, come into play, obscuring the former pathway with increasing potential. Thus, **C2** reacts remarkably different from **R1** and **C1** in the lowest energy pathway. Upon reduction to the Co^I^ intermediate, one ligand‐based reduction initiates catalysis by double protonation. Likely the basicity of the [H−Co^II^−Co^I^] is increased by electronic coupling to the neighboring Co^I^ and thus allows for its protonation.

Computationally a slight increase in the atomic charge of the cobalt atom in a metal‐to‐metal electron transfer is found. For **R1** and **C1** further reduction was required to increase the basicity of the hydride. In agreement with this notion, the first protonation is two to three orders of magnitude faster than the second is. It should be noted, however, that the (EE)ECEC mechanism as found for **C1** and **R1** dominates catalysis and, albeit at higher overpotential, gives rise to a beneficial catalytic Tafel plot (see Figure 5).

In all three cases, the addition of the weaker AcOH results in catalytic hydrogen production at the same potentials, but with a slower increase of hydrogen production, as is evident from the LSV scans with inline H_2_ detection in Figure 3. This indicates a slower hydrogen formation rate, which is confirmed by FOWA of the scans: rates of 26±8, 170±60, and 630±240 m ^−1^ s^−1^ were found for **R1**, **C1**, and **C2**, respectively (ECEC pathway, see Figure 5 and Table SI8). As in the case of [HNEt_3_](BF_4_), protonation of reduced **C2** is significantly faster than for **R1** and **C1**, and a slow but visible sign of the (E)EECC mechanism is found for **C2**. The two orders of magnitude decrease in rate from [HNEt_3_](BF_4_) to AcOH is in line with the increase of p*K*_a_ (9.2 to 13.5), and consistent with literature data.^[57]^ Faradaic efficiencies (FEs) between 0.80 and 0.89 were found for the complete LSV scan (Table SI9), indicating alternative reductive pathways other than hydrogen formation.

Also, in the presence of the strongest acid H(BF_4_), hydrogen formation was confirmed in all three cases. In stark contrast to the weaker acids, H(BF_4_) shifts the onset of H_2_ production anodically for all three compounds (Figure 3, bottom). In the presence of this strong proton source, H_2_ begins to evolve directly after the reduction to Co^I^ (**R1**), resp. Co^I^−Co^I^ (**C1** and **C2**). As opposed to the weaker acids, H(BF_4_) is capable to protonate the Co^I^ intermediates. Rate limiting constants extracted from LSV by FOWA give 110±40, 0.93±0.39, and 280±110 m ^−1^ s^−1^ (see Figure 5 and Table SI8). Although the catalytic half wave potential *E*_cat/2_ shifts significantly in anodic direction, the shift in $E_{H^{+}/H_{2}}^{0}$ from [HNEt_3_](BF_4_) to H(BF_4_) (−1.21 to −0.71 V vs. Fc/Fc^+^)^[34]^ is not compensated for **R1** and **C2**, or at the cost of a very slow rate (**C1**).

The elucidation of the rate‐limiting step (TOF_max_) and the respective catalytic half wave potential *E*_cat/2_ relative to the potential of proton reduction with the respective acid ($E_{H^{+}/H_{2}}^{0}$ ) can be used to benchmark catalysts.[^52^, ^53^, ^54^, ^58^, ^59^] Comparison with [Co^II^(dmgH)_2_py] and [Fe^II^(TPP)], both with [HNEt_3_]^+^ in DMF, and [Ni^II^(P_2_^Ph^N^Ph^)_2_]^2+^ using [HDMF]^+^ in MeCN, is given in the Supporting Information (Figure SI22). Clearly, [Co^II^(dmgH)_2_py] is unrivalled in terms of overpotential. Interestingly, protonation with the relatively weak acid [HNEt_3_]^+^ occurs at Co^I^ for the latter, as opposed to the cobalt polypyridyl catalysts investigated here, or elsewhere.[^10^, ^11^, ^49^] This, in turn, explains the substantial difference in overpotential, and might be related to the spin state of the respective Co^I^ intermediate: Tong and co‐workers recently demonstrated a high‐spin S=1 state for a similar Co^I^ intermediate, derived from a S=3/2 Co^II^.^[49]^ These results coincide with a recent study on **R1** and a related cobalt polypyridyl, evidencing the importance of the Co^I^ singlet state for protonation.[^36^, ^37^] [Co^II^(dmgH)_2_py], however, is a low spin system, and might give rise to a low spin Co^I^, and thus facilitate protonation on a full $d_{z^{2}}$ orbital (e. g., oxidative addition to a d^8^ system). The [Ni^II^(P_2_^Ph^N^Ph^)_2_]^2+^ catalyst of DuBois and co‐workers together with the [Fe^II^(TPP)] as described by Savéant and co‐workers,^[58]^ are still one to two orders of magnitude faster than the mononuclear **R1**, or **C1**, with [HNEt_3_]^+^, at comparable to slightly worse overpotentials. **C2**, however, in the (EE)ECEC pathway and [HNEt_3_]^+^, reaches a TOF_max_ of 10^5^ s^−1^, similar to [Fe^II^(TPP)], but at 250 mV more anodic potentials.

Besides rate and overpotential, stability, preferably in water, is a very important merit for a hydrogen production catalyst. We thus investigated the three catalysts in photochemical hydrogen production in a system with [Ru(bipy)_3_]^2+^ as photosensitizer (PS), ascorbic acid (AscOH) as electron donor, and tris(2‐carboxyethyl)phosphine (TCEP) to regenerate spent AscOH (Figure SI28). TOFs (mol H_2_ per mol of catalyst per second) between 0.1 and 1 were found, three orders of magnitude lower than what was found in electrochemistry in DMF.

This is, however, expected, as a reasonably fast catalyst is not limiting turnover in this photochemical setup. Concerning stability, turnover numbers (TONs) above 20000 have been found for **R1** and **C2**, and about 7000 for **C1**. In the photochemical cycle, the catalysts are reduced by the reduced dye [Ru(bipy)_3_]^+^, formed by reductive quenching of the excited dye by ascorbic acid. Interestingly, *E*([Ru(bipy)_3_]^2+/+^) is at −1.74 V vs. Fc/Fc^+^, allowing reduction to the Co^I^ state, but around 100 mV anodic of the second cobalt‐based reduction. In line with this, an {EC}EC mechanism has been proposed below pH 7, with {} signifying a coupled electron‐proton transfer. Above pH 7, an E{EC}C mechanism was proposed for a very closely related system.^[4]^

The high stability of the systems is further substantiated by a 7‐day chronoamperometry (CA) performed on the three catalysts in DMF with 200 mm [HNEt_3_](BF_4_), as summarized in Table 1 (see also Figures SI26 and SI27). Decrease in activity in such experiments is assigned to catalyst deactivation and can be quantified by exponential fitting of charge and product build‐up.^[59]^ Towards this end, *t*_chem_ as described in ref. 59 of 2 to 4 days were found. This is roughly one order of magnitude higher than a related system using an amine‐containing polypyridyl ligand in DMF for H_2_ production,^[10]^ or iron porphyrins for electrocatalytic CO_2_ reduction.^[59]^ In the case of **R1**, a limiting TON of around 90 was calculated, whereas 320 was found for **C1** and **C2**. A [HNEt_3_](BF_4_) addition experiment at 6 days leads to a partial recovery of electrocatalysis, further substantiating the high stability of the latter two catalysts in DMF. As compared to the FOWA analysis, TOF_max_ values extracted from the CA runs are one order of magnitude lower for **R1**, but in‐line for **C1** and **C2** (see colored stars in Figure). Possibly the faster deactivation of **R1** compared to **C1** and **C2** prevents the former system from achieving the high rates observed in the fast CV experiments. We speculate that the (E)EECC pathway results in faster catalyst deactivation in comparison to the (E)ECEC mechanism since lower oxidation states are achieved in the former. Possibly the dual‐cores mediate this by distributing the charge across both cobalt cores, thereby increasing stability under these harsh conditions.

**Table 1:** Summary of CA.^[a]^

Compound	*E*_app_ [V]	*v*_init_ [nmol s^−1^]	TOF_max_ [s^−1^]	TON^lim^	FE	***t***_**chem**_ [h]
R1	−1.80	0.9±0.1	340±100	88±1	0.89±0.03	54±1
C1	−1.80	1.6±0.1	450±150	319±1	0.84±0.03	113±1
C2	−1.70	1.6±0.1	2500±350	324±1	0.88±0.03	114±1

[a] 400 μm **R1**, **C1**, and **C2**, 200 mm [HNEt_3_](BF_4_), 0.1 m [TBA](PF_6_), 5 mL DMF; working electrode was Hg pool, reference electrode was Ag/AgCl, V was given vs. Fc/Fc^+^, counter‐electrode is Pt separated by a glass frit (Figures SI26, SI27). *E* ^0^ ${}_{H{}^{+}/H{}_{2}}$ for 200 mm [HNEt_3_](BF_4_) in DMF was calculated at −1.19 V,^[34]^ *R*_u_ was 60±10 Ω.

## Conclusion

We herein report on the synthesis and characterization of two new dinuclear cobalt polypyridyl water reduction catalysts containing equivalent pentadentate cobalt‐binding sites linked by either a bipyridyl or a pyrazyl bridge. Comparison to the equivalent mononuclear reference compound **R1** allowed to draw correlations of electronic and, more importantly, catalytic behavior with structure and number of cores.

This work demonstrated cooperativity of the cobalt centers in the dual‐core catalyst featuring electronically coupled cobalt centers, as compared to the single‐core catalyst or the electronically separated dual‐core, with respect to rates and overpotentials. We hypothesize that an increase in p*K*_a_, and thus rate, results from electronic interaction of the two cobalt centers. Additionally, an increase in stability resulted for the dual‐core catalysts, which deserves further investigation.

## Experimental Section

### Analytical methods

**Photocatalysis**: Before use, the catalysis vials were washed by immersion in iPrOH/KOH for at least 48 h and rinsing with doubly distilled water. The standard concentrations for the electron relay, the SED and the photosensitizer are 0.1 m NaAsc, 0.1 m TCEP, and 500 mm Ru(bpy)_3_Cl_2_, respectively. NaAsc, TCEP, and PS were weighted directly into the vial with appropriate accuracy and the (much lower concentrated) WRC was added by taking an aliquot of a stock solutions (200 μm) in H_2_O. pH was adjusted by NaOH (2 m) or TFA (2 m). An LED (453 nm) served as light source for photoexcitation and a constant flow of aq. sat. Ar (6.0 mL min^−1^) through the catalytic solution followed by a tube filled with drying agent (3 Å molecular sieves) and connected to a GC allowed for frequent analysis of the composition of the gaseous phase (Bruker GC‐450 gas chromatograph with argon as carrier gas and a 3 μm×2 mm packed molecular sieve 13X 80–100 column). Injection volume was 1000 μL. The column and reference gas flow (Ar) was set to 20 mL min^−1^. The oven was operated isothermally at 100 °C. The gases were detected using a thermal conductivity detector operated at 150 or 50 °C. Retention time was about 1 min for H_2_. The detection limit of this setup was 0.3 pmol${}_{H{}_{2}}$ s^−1^.). A Metrohm Autolab PGSTAT302N was used to apply potential and to record the current. Evaluation and illustration of the results were performed with OriginPro 2017.

**Figure 6:**
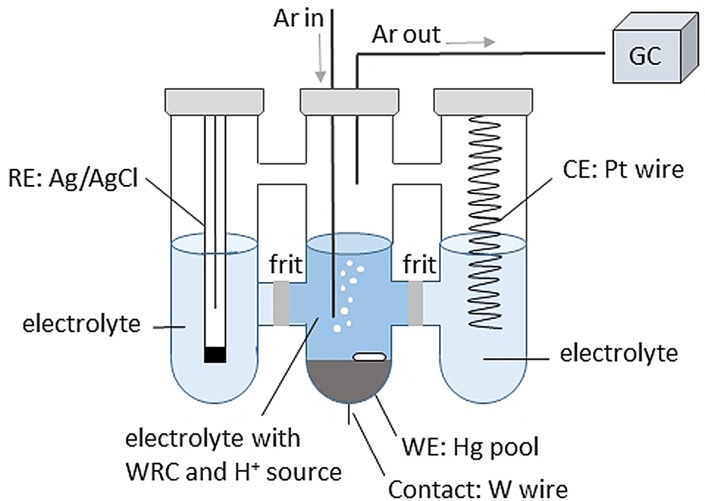
Experimental setup for LSV. Details can be found in the main text.

**LSV and CA**: LSV and CA experiments were conducted using a three‐electrode set‐up, with a Pt coil serving as counter electrode, an Ag/AgCl reference electrode, and an Hg pool as working electrode. The Hg pool was connected to the potentiostat by means of a tungsten wire. The electrodes were separated from each other by P4 glass filter frits and only the working electrode was exposed to catalyst and proton source in 5 mL electrolyte (dark blue region in Figure 6). The reference electrode was calibrated regularly against Fc/Fc^+^. A steady stream of DMF‐saturated Ar (6.0 L min^−1^) through the WE chamber followed by a tube filled with drying agent (3 Å molecular sieves) and connected to a GC allowed for frequent analysis of the composition of the gaseous phase.

### Synthesis

Appropriate analytical spectra of the compounds are provided in the Supporting Information (Figures SI1–SI3).

**6,6’‐di(di‐(6‐bipyridyl)‐methanol)bipyridine (L1)**: A solution of 6,6’‐dibromo‐2,2’‐bipyridine (0.3 mmol, 100 mg) in 8 mL dry THF was added dropwise to a solution of *n*‐butyllithium (380 μL of a 1.6 m solution in hexane) in 4 mL dry THF at a rate so that the temperature never exceeded −95 °C and stirred for 30 min. A solution of di‐(bipyridine‐6‐yl)‐methanone (0.6 mmol, 207 mg) in 7 mL THF was added dropwise along the glass wall of the flask, which was deeply immersed in the cooling bath by means of a syringe. The glass wall subsequently rinsed with 2 mL dry THF. The mixture was allowed to reach −40 °C and quenched with MeOH. The crude mixture was extracted with DCM from water (3×) and subjected to flash column chromatography (C_18_‐silica, flash master, H_2_O/MeCN, 60 : 40, +10 % MeCN per 100 mL). The pure fractions were combined to obtain the product as white solid (95.4 mg, 37 %). ^1^H NMR (400 MHz, CDCl_3_): *δ*=8.64 (*dq*, *J*=4.81 Hz, *J*=0.81 Hz, 4H), 8.35 (*dd*, *J*=7.6 Hz, *J*=1.03 Hz, 4H), 8.20 (*d, J*=7.96 Hz, 4H), 8.15 (*dd, J*=7.72 Hz, *J*=0.82 Hz, 2H), 7.90–7.81 (*m*, 10H), 7.73 (*t, J*=7.84 Hz, 2H), 7.66 (*td, J*=7.84 Hz, *J*=1.73, 4H), 7.51 (*s, J=*7.92 Hz, 2H), 7.25–7.23 ppm (*m*, 4H). ^13^C NMR (400 MHz, CDCl_3_): *δ*=160.8, 154.5, 152.2, 152.1, 147.6, 135.8, 135.7, 135.4, 122.2, 122.0, 121.9, 121.8, 119.6, 118.0, 115.0, 64.4 ppm.

**2,4‐di(di‐(6‐bipyridyl)‐methanol)pyrazine (L2)**: A solution of 2,5‐dibromopyrazine (0.14 mmol, 33 mg) in 4 mL dry THF was added dropwise to a solution of *n*‐butyllithium (175 μL of a 1.6 m solution in hexane) in 3 mL dry THF at −90 °C. After 1 h of additional stirring, a solution of di‐(bipyrin‐6‐yl)‐methanone (0.29 mmol, 100 mg) in 4 mL dry THF was added dropwise, and the cooling bath was subsequently removed. The reaction was quenched at −50 °C with MeOH and the solvent evaporated under reduced pressure. The crude mixture was taken up in aq. K_2_CO_3_ and extracted with DCM (3×). The combined organic phases were dried over MgSO_4_ and the solvent evaporated under reduced pressure. The crude mixture was subjected to FC chromatography (C_18_‐silica, flash master, H_2_O/MeCN, 70 : 30, +20 % MeCN per 150 mL). The pure fractions were combined, and the solvent was evaporated under reduced pressure to yield the pure product as white solid (17 mg, 16 %). ^1^H NMR (400 MHz, CDCl_3_): *δ*=9.06 (*s*, 2H), 8.55 (*d, J*=4.76 Hz, 4H), 8.62 (*dd, J*=6.96 Hz, *J*=1.80 Hz, 4H), 8.15 (*d, J*=8.00 Hz, 4H), 7.87–7.81 (*m*, 8H), 7.59 (*td, J*=7.68 Hz, *J*=1.72 Hz, 4H), 7.22 ppm (*ddd*, *J*=7.44 Hz, *J*=4.80 Hz, *J*=1.00 Hz, 4H). No suitable ^13^C NMR spectrum was recorded due to the small‐scale synthesis.

**[Co_2_(2,4‐di(di‐(6‐bipyridyl)‐methanol)bipyridine)](BF_4_)_4_ (C1)**: A solution of Co(BF_4_)_2_×6H_2_O (2.2 equiv.) in 10 mL MeOH was added to the ligand **L1** (0.16 mmol, 130 mg) in 10 mL CHCl_3_ and stirred for 5 min. The solvent was evaporated under reduced pressure and the solid triturated in THF, filtered, and washed with THF until the filtrate was not colored anymore. The pure product was washed through the filter with MeCN and obtained after freeze‐drying as brownish solid (187 mg, 90 %). HRMS (ESI‐MS) *m*/*z*: [Co_2_L‐H^+^]^3+^ Calcd for C_52_H_35_Co_2_N_10_O_2_ 316.38640; Found: 316.38694. UHPLC‐MS *m*/*z*: [Co_2_L‐H^+^]^3+^ calcd for C_52_H_35_Co_2_N_10_O_2_: 316.72; found 316.72. *R*_f_=1.3/5 min. Crystals suitable for X‐ray crystallography were obtained by the vapor diffusion method from MeCN with CHCl_3_ as anti‐solvent.

**[Co_2_(2,4‐di(di‐(6‐bipyridyl)‐methanol)pyrazine)](BF_4_)_4_ (C2)**: A solution of Co(BF_4_)_2_ (2.5 equiv.) in 1.3 mL MeOH was added to the ligand (**L2**) (0.02 mmol, 16.9 mg) in 1 mL CHCl_3_ and stirred for 30 min. The solvent was evaporated under reduced pressure and the solid triturated in THF, filtered, and washed with THF until the filtrate was not colored anymore. The pure product was washed through the filter with MeCN and the solvent removed to obtain the pure product according to UHPLC‐MS (see Supporting Information). The product was purified by crystallization by the vapor diffusion method in MeCN with CHCl_3_ as anti‐solvent. No appropriate yield could be determined due to the small‐scale synthesis. HRMS (ESI‐MS) *m*/*z*: [Co_2_L‐(H^+^)]^3+^ Calcd for C_46_H_31_Co_2_N_10_O_2_: 291.04263; Found: 291.04261. UHPLC‐MS *m*/*z*: [Co_2_L −2(H^+^)]^2+^ calcd for C_46_H_30_Co_2_N_10_O_2_: 436.52; found 436.34. *R*_f_=1.3/5 min.

## Conflict of interest

The authors declare no conflict of interest

1

## Supporting information

As a service to our authors and readers, this journal provides supporting information supplied by the authors. Such materials are peer reviewed and may be re‐organized for online delivery, but are not copy‐edited or typeset. Technical support issues arising from supporting information (other than missing files) should be addressed to the authors.

Supporting Information

## Data Availability

The data that support the findings of this study are available in the supplementary material of this article.
