# Supplementary material for: Two Novel Dinuclear Cobalt Polypyridyl Complexes in Electro‐ and Photocatalysis for Hydrogen Production: Cooperativity Increases Performance
Source: ChemSusChem. 2022 Jul 21;15(17):e202201049. doi: 10.1002/cssc.202201049 (PMC9545343; doi:10.1002/cssc.202201049)
Supplement: Supplementary file 1 — Supporting Information [file CSSC-15-0-s001.pdf]

ChemSusChem

Supporting Information

Two Novel Dinuclear Cobalt Polypyridyl Complexes in Electro- and Photocatalysis for Hydrogen Production: Cooperativity Increases Performance

Nicola Weder+, Nora S. Grundmann+, Benjamin Probst, Olivier Blacque, Rangsiman Ketkaew, Fabrizio Creazzo, Sandra Luber, and Roger Alberto*© 2022 The Authors. ChemSusChem published by Wiley-VCH GmbH. This is an open access article under the terms of the Creative Commons Attribution License, which permits use, distribution and reproduction in any medium, provided the original work is properly cited.

Table of Contents:

1 Experimental ... 2

1.1 Analytical Methods ... 2

1.2 Synthesis ... 3

2 Crystallographic data ... 7

3 Electrochemistry ... 10

3.1 Cyclovoltammetry ... 10

3.2 Electrocatalysis ... 12

3.3 FOWA analysis of CV Data ... 13

3.4 LSV Experiments ... 19

3.5 CA Experiments ... 24

4 Photocatalytic Experiments in H_2O ... 26

5 References ... 27

6 Computational details ... 28

7 Studied system ... 29

8 Computational Models of Dinuclear Complexes ... 30

9 Computed Reduction Potentials ... 31

10 Population Analysis ... 33

11 Intramolecular Electron Transfer ... 34

12 Molecular Orbitals ... 35

13 References (Computation) ... 50

1

1 Experimental

1.1 Analytical Methods

Crystallography: Single crystal X-ray diffraction data were collected at 160(1) K on a Rigaku OD Synergy (Pilatus 200K detector) diffractometer equipped with an Oxford liquid-nitrogen Cryostream cooler using a single wavelength X-ray source from a micro-focus sealed X-ray tube with the Cu Kα radiation (λ= 1.54184 Å). The selected single crystals were mounted using polybutene oil on a flexible loop fixed on a goniometer head and transferred to the diffractometer. Pre-experiments, data collections, data reductions and analytical absorption corrections[1] were performed with the program suite *CrysAlisPro*. Using *Olex2*,[2] the structures were solved with the SHELXT[3] small molecule structure solution program and refined with the *SHELXL2018/3* program package[4] by full-matrix least-squares minimization on F^2. The crystal data collections and structure refinement parameters are summarized in Tables S1 – S2. CCDC 1990212 (for **L1**), 1990213 (for **C2**) and 1990214 (for **C1**) contain the supplementary crystallographic data for these compounds, and can be obtained free of charge from the Cambridge Crystallographic Data Centre via www.ccdc.cam.ac.uk/data_request/cif.

In the crystal structure of **C1**, the dinuclear species has a 3+ charge and co-crystallized with BF_4^- counterions and solvent molecules of methanol and water in a ratio 1/3/1/1.25. Two counterions are disordered over two sets of positions with site-occupancy factors of 0.500(6)/0.500(6) and 0.475(6)/0.525(6). One bipyridine ligand (including the coordinated COH group) is also disordered over two sets of positions, the site-occupancy factors of the two parts were refined as 0.465(4) and 0.535(4). Many restraints had to be used to correct the geometry of the disordered groups and the thermal parameters of the corresponding non H atoms. In the crystal structure of **C2**, the dinuclear species lies on a two-fold axis. Only half of the molecule had to be refined, the other part was reproduced by a symmetry operation. The asymmetric unit contains half of the main molecule, two BF_4^- counterions occupying three different positions (two of them located on a two-fold axis) and one solvent molecule of chloroform, disordered over two sets of positions with site-occupancy factors of 0.443(5) and 0.557(5). In the third crystal structure, the ligand **L1** lies on a center of inversion. Only one half of the molecule is refined, the other part is reproduced by a symmetry operation. Solvent molecules of chloroform cocrystallized with **L1** in a ratio 1/2.

UHPLC-MS: UPLC-MS spectra (low resolution) were measured on an ACQUITY UPLC BEH C_{18} column (1.7 µm, 2.1x50mm). The solvents used were of LCMS grade and the eluent composed as following: 0.1% formic acid (A), acetonitrile (B). Gradient: 0 min: 95% A, 5% B, 0.5 min: 95% A, 5% B, 4 min: 100% B, 5 min 100% B.

High-resolution ESI-MS spectra: The samples were dissolved in an appropriate solvent at a concentration of around 1 µmol/mL and measured at a continuous flow of 3 µL/min. The Bruker maXis QTof high-resolution mass spectrometer (Bruker GmbH, Bremen, Germany) was operated in the positive or negative electrospray ionization mode at 4'000 V capillary voltage, -500 V endplate offset, with a N2 nebulizer pressure of 0.8 bar and dry gas flow of 4 L min-1 at 180 °C. MS acquisitions were performed in the mass range from m/z 50 to 2'000 at 20'000 resolution (full width at half maximum) and 1.0 Hz spectra rate. Masses were calibrated prior analysis and below 2 ppm accuracy between m/z 158 and 1450 with a 2 mM solution of sodium formate or between m/z 118 and 2721 with a Fluka electrospray calibration solution (Sigma-Aldrich, Buchs, Switzerland) that has been 100 times diluted with acetonitrile, respectively.

Cyclic Voltammetry: Cyclic voltammetry was recorded with a *Biologic SP-300*, under N_2 atmosphere in DMF (0.1 M $TBAPF_6$) with a glassy carbon working electrode, a Pt counter electrode and an Ag/AgCl reference. Scan rates, catalyst and substrate concentrations as indicated. Calibration of the reference electrode against Fc was performed with the same setup. Acids were added neat and incrementally, either *via* Eppendorff pipette (HBF_4 x 2 Et_2O, AcOH) or as solid ($HNEt_3BF_4$).

1.2 Synthesis

The synthetic details are described in the experimental section of the main text.

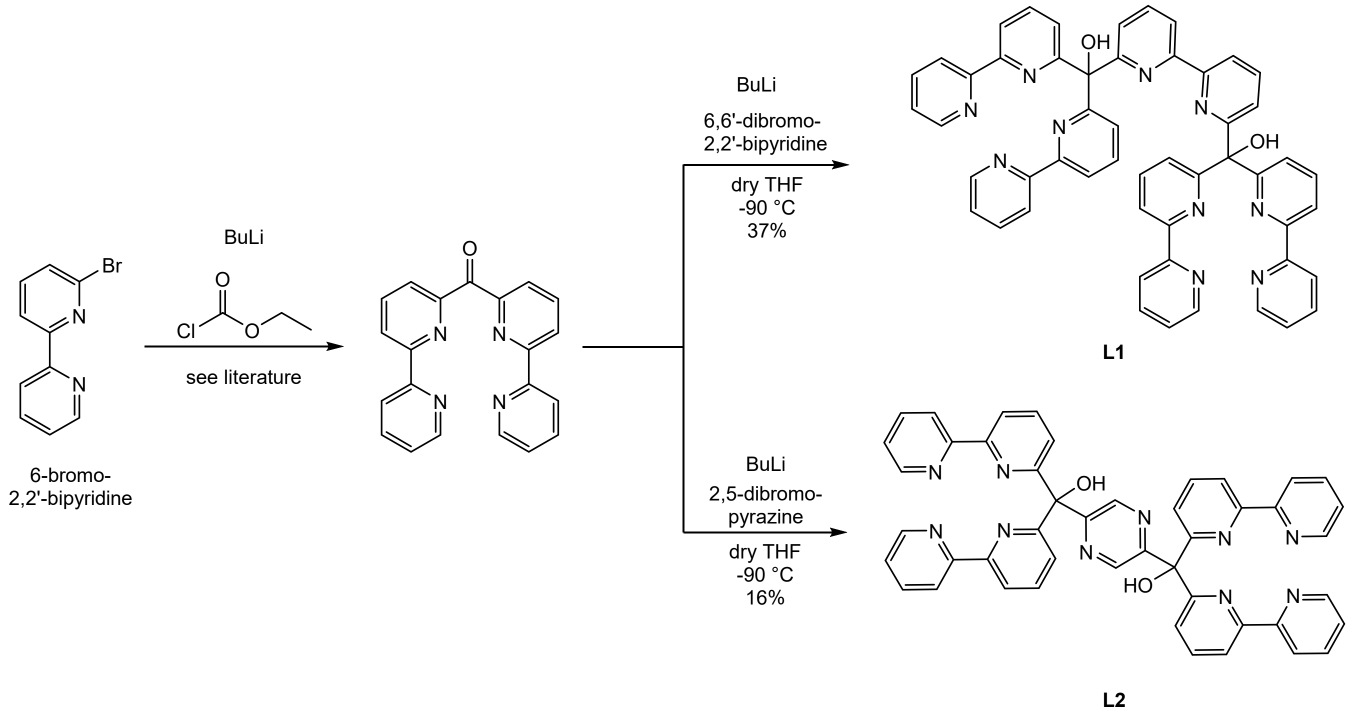

Scheme SI 1: Synthetic pathway from the commercially available 6-bromo-2,2'-bipyridine via two steps to the ligands **L1** and **L2**, respectively.

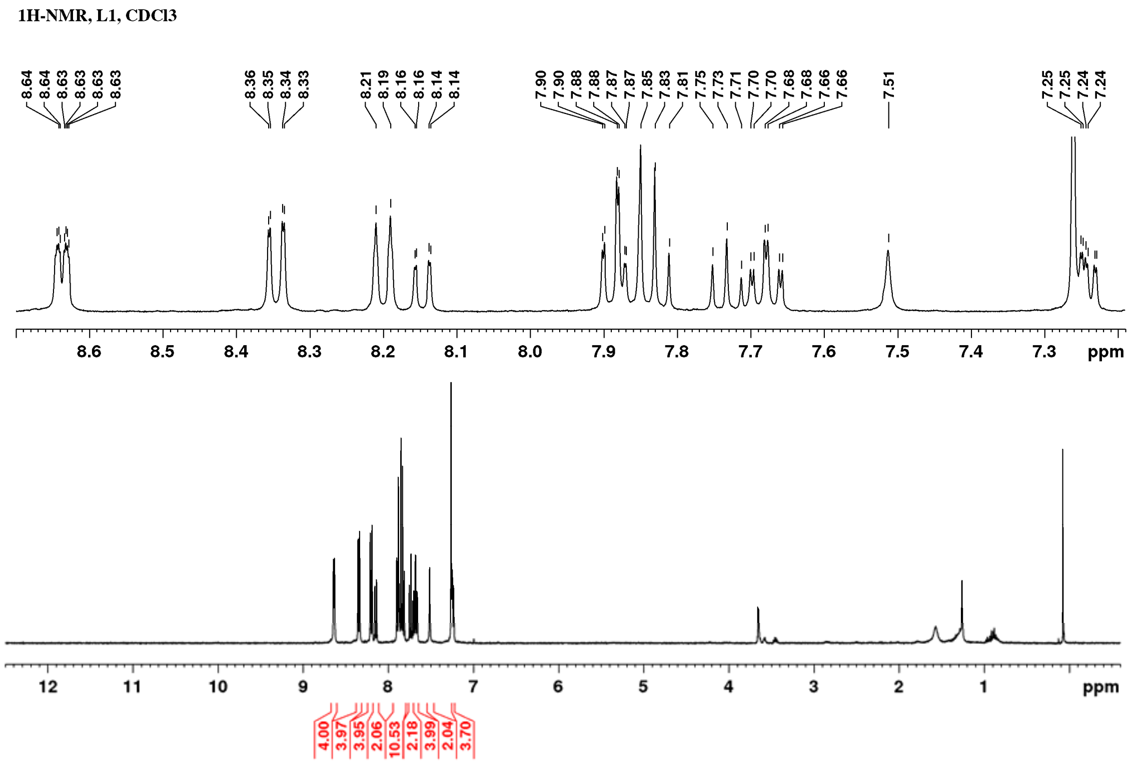

Figure SI1: ^{1}H-NMR of the ligand **L1** in $CDCl_3$. The signal at 7.25 ppm overlaps with the solvent signal ($CHCl_3$), thus, the integral of 3.7 should be corrected to 2.

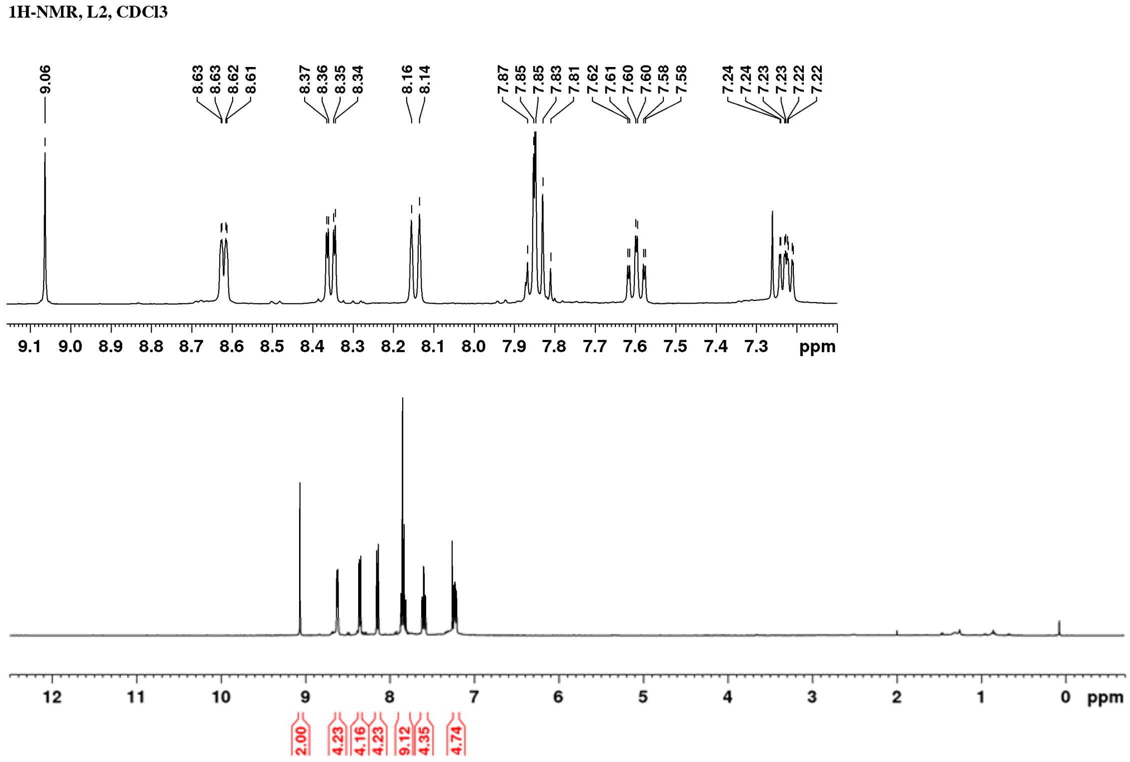

Figure SI2: ^{1}H-NMR of ligand **L2** in $CDCl_3$.

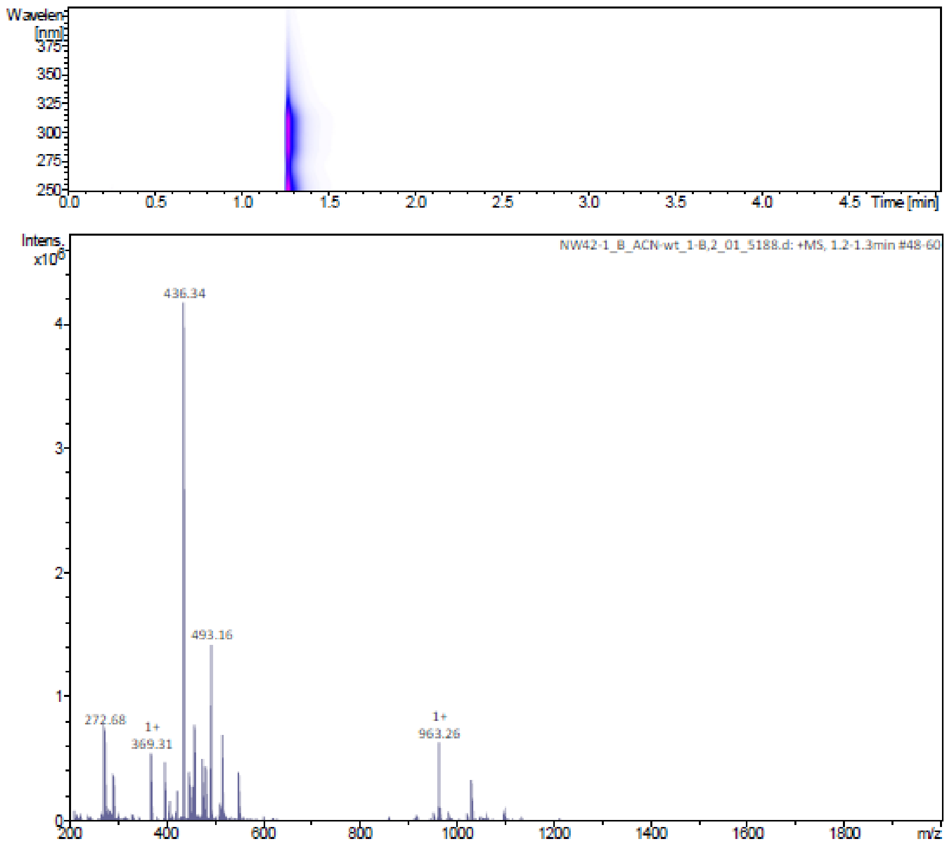

Figure SI3: UPLC-MS-trace of the pure compound C2. calcd for C46H30Co2N10O2: 436.52; found 436.34. Rf = 1.3 / 5 min.

2 Crystallographic data

Table SI1. Crystal data and structure refinement parameters for C1 and C2.

	C1	C2
CCDC number	1990214	1990213
Empirical formula	$C_{212}H_{170}B_{12}Co_8F_{52}N_{40}O_{17}$	$C_{52}H_{40}B_4Cl_6Co_2F_{16}N_{12}O_2$
Formula weight	5139.03	1542.76
Temperature/K	160(1)	160(1)
Crystal system	monoclinic	monoclinic
Space group	C2/c	I2
a/Å	15.6270(3)	12.87084(13)
b/Å	22.4882(4)	17.4783(2)
c/Å	30.4238(6)	13.83815(16)
α/°	90	90
β/°	92.323(2)	99.7576(10)
γ/°	90	90
Volume/$Å^3$	10682.8(4)	3068.00(6)
Z	2	2
$\rho_{calc}g/cm^3$	1.598	1.670
μ/mm^{-1}	5.782	7.546
F(000)	5204.0	1544.0
Crystal size/mm^3	0.21 × 0.207 × 0.118	0.17 × 0.15 × 0.09
Radiation	CuKα (λ = 1.54184)	CuKα (λ = 1.54184)
2Θ range for data collection/°	6.892 to 149.004	8.224 to 148.994
Index ranges	-18 ≤ h ≤ 19, -28 ≤ k ≤ 27, -35 ≤ l ≤ 38	-16 ≤ h ≤ 16, -21 ≤ k ≤ 19, -17 ≤ l ≤
Reflections collected	51347	42466
Independent reflections	10888 [R_{int} = 0.0745, R_{sigma} = 0.0534]	6198 [R_{int} = 0.0408, R_{sigma} = 0.0246]
Data/restraints/parameters	10888/661/1004	6198/74/457
Goodness-of-fit on F^2	1.031	1.051
Final R indexes [I>=2σ (I)]	R_1 = 0.0668, wR_2 = 0.1843	R_1 = 0.0641, wR_2 = 0.1800
Final R indexes [all data]	R_1 = 0.0905, wR_2 = 0.2029	R_1 = 0.0693, wR_2 = 0.1864
Largest diff. peak/hole / e $Å^{-3}$	0.96/-0.91	1.03/-0.65
Flack parameter	-	-0.020(3)

Table SI2. Crystal data and structure refinement parameters for L1**.**

	L1
CCDC number	1990212
Identification code	NW1112
Empirical formula	$C_{54}H_{38}Cl_6N_{10}O_2$
Formula weight	1071.64
Temperature/K	160(1)
Crystal system	triclinic
Space group	P-1
a/Å	7.8686(3)
b/Å	12.7099(5)
c/Å	12.7912(5)
α/°	93.389(3)
β/°	98.336(3)
γ/°	100.340(3)
Volume/Å^3	1240.39(8)
Z	1
ρ_{calc}g/cm^3	1.435
μ/mm^{-1}	3.600
F(000)	550.0
Crystal size/mm^3	0.11 × 0.03 × 0.03
Radiation	CuKα (λ = 1.54184)
2Θ range for data collection/°	7.012 to 148.928
Index ranges	-9 ≤ h ≤ 9, -14 ≤ k ≤ 15, -15 ≤ l ≤ 15
Reflections collected	20003
Independent reflections	5051 [R_{int} = 0.0280, R_{sigma} = 0.0271]
Data/restraints/parameters	5051/0/329
Goodness-of-fit on F^2	1.077
Final R indexes [I>=2σ (I)]	R_1 = 0.0467, wR_2 = 0.1317
Final R indexes [all data]	R_1 = 0.0516, wR_2 = 0.1358
Largest diff. peak/hole / e Å^{-3}	0.56/-0.54

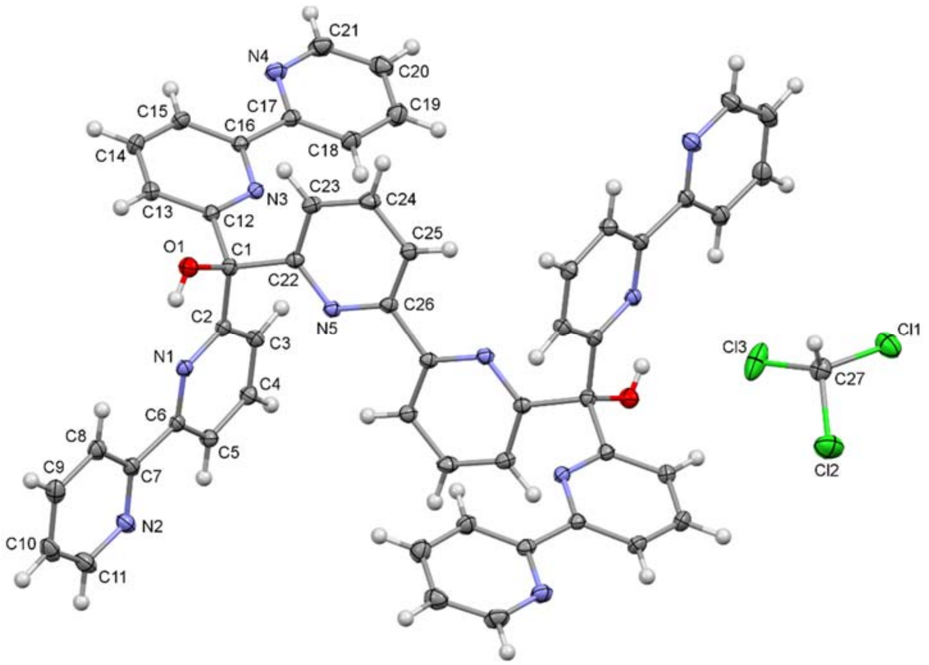

Figure SI4: The molecular structure of **L1** with displacement ellipsoids drawn at the 30% probability level.

3 Electrochemistry

3.1 Cyclovoltammetry

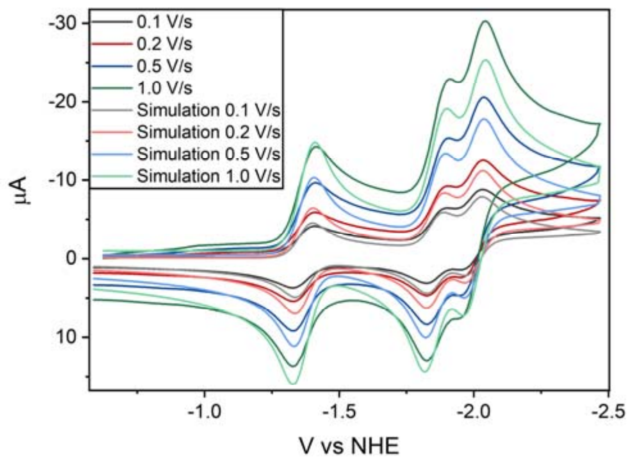

Figure SI5: Cyclic voltammogram of **R1** (1 mM) in DMF, containing 100 mM $TBAPF_6$ and fitting in DigiElch,[5-10] using the parameters in **Table SI3**.

Table SI3: Charge transfer reactions and parameters used for fitting of **R1** using DigiElch 8. D = 2.8E-6 cm2/s, Ru = 600 Ω, Cdl = 1E-6 F, T = 298.5 K, electrode area S = 0.0314 cm2, geometry planar, diffusion semi-infinite 1D. Fitted values are cursive.

Charge Transfer Reactions	$E_{1/2}$ (V vs Fc/Fc^+)	α	k_s (cm/s)
L-Co^{II} + e^- => L-Co^I	-1.37	0.5	10000
L-Co^I + e^- => L^{-1}-Co^I	-1.85	0.5	10000
L^{-1}-Co^I + e^- => L^{-2}-Co^I	-2.00	0.5	10000

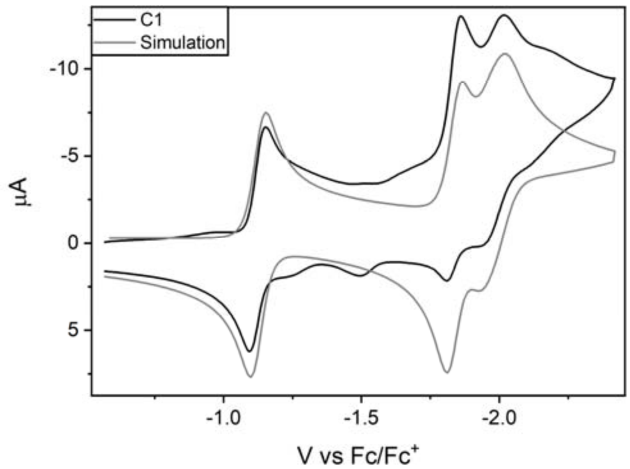

Figure SI6: Cyclic voltammogram of **C1** (1 mM) in DMF (0.1 V/s), containing 100 mM $TBAPF_6$ and simulation in DigiElch,[5-10] using the parameters in **Table SI4.**

Table SI4: Charge transfer reactions and parameters used for simulation of **C1** using DigiElch 8. D = 1.53E-6 cm2/s, Ru = 600 Ω, Cdl = 3E-6 F, T = 298.5 K, electrode area S = 0.0314 cm2, geometry planar, diffusion semi-infinite 1D.

Charge Transfer Reactions	$E_{1/2}$ (V vs Fc/Fc$^+$)	α	k_s (cm/s)
L-Co^{II}-Co^{II}-L + e^- => L-Co^{I}-Co^{II}-L	-1.12	0.5	10000
L-Co^{I}-Co^{II}-L + e^- => L-Co^{I}-Co^{I}-L	-1.13	0.5	10000
L-Co^{I}-Co^{I}-L + e^- => L^{-1}-Co^{I}-Co^{I}-L	-1.83	0.5	10000
L^{-1}-Co^{I}-Co^{I}-L + e^- => L^{-1}-Co^{I}-Co^{I}-L^{-1}	-1.84	0.5	10000
L^{-1}-Co^{I}-Co^{I}-L^{-1} + e^- => L^{-2}-Co^{I}-Co^{I}-L^{-1}	-1.95	0.5	10000
L^{-2}-Co^{I}-Co^{I}-L^{-1} + e^- => L^{-2}-Co^{I}-Co^{I}-L^{-2}	-2.01	0.5	10000

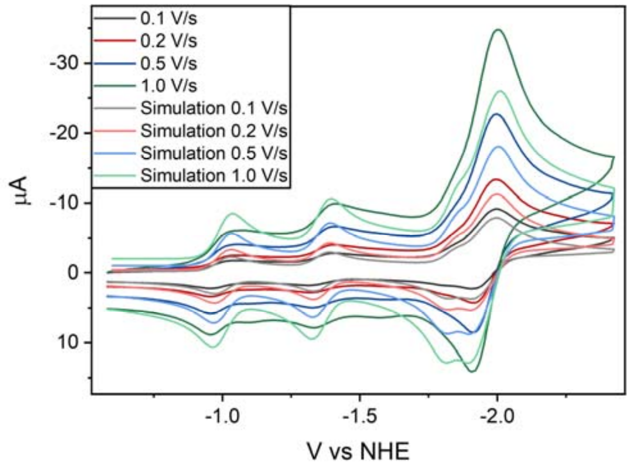

Figure SI7: Cyclic voltammogram of **C2** (0.55 mM) in DMF, containing 100 mM $TBAPF_6$ and simulation in DigiElch,5-10 using the parameters in **Table SI5**.

Table SI5: Charge transfer reactions and parameters used for simulation of **C2** using DigiElch 8. D = 2.0E-6 cm^2/s, R_u = 600 Ω, C_{dl} = 2E-6 F, T = 298.5 K, electrode area S = 0.0314 cm^2, geometry planar, diffusion semi-infinite 1D.

Charge Transfer Reactions	$E_{1/2}$ (V vs Fc/Fc^+)	α	k_s (cm/s)
L-Co^{II}-Co^{II}-L + e^- = L-Co^{I}-Co^{II}-L	-1.00	0.5	10000
L-Co^{I}-Co^{II}-L + e^- = L-Co^{I}-Co^{I}-L	-1.36	0.5	10000
L-Co^{I}-Co^{I}-L + e^- = L^{-1}-Co^{I}-Co^{I}-L	-1.83	0.5	10000
L^{-1}-Co^{I}-Co^{I}-L + e^- = L^{-1}-Co^{I}-Co^{I}-L^{-1}	-1.91	0.5	10000
L^{-1}-Co^{I}-Co^{I}-L^{-1} + e^- = L^{-2}-Co^{I}-Co^{I}-L^{-1}	-1.96	0.5	10000
L^{-2}-Co^{I}-Co^{I}-L^{-1} + e^- = L^{-2}-Co^{I}-Co^{I}-L^{-2}	-2.00	0.5	10000

3.2 Electrocatalysis

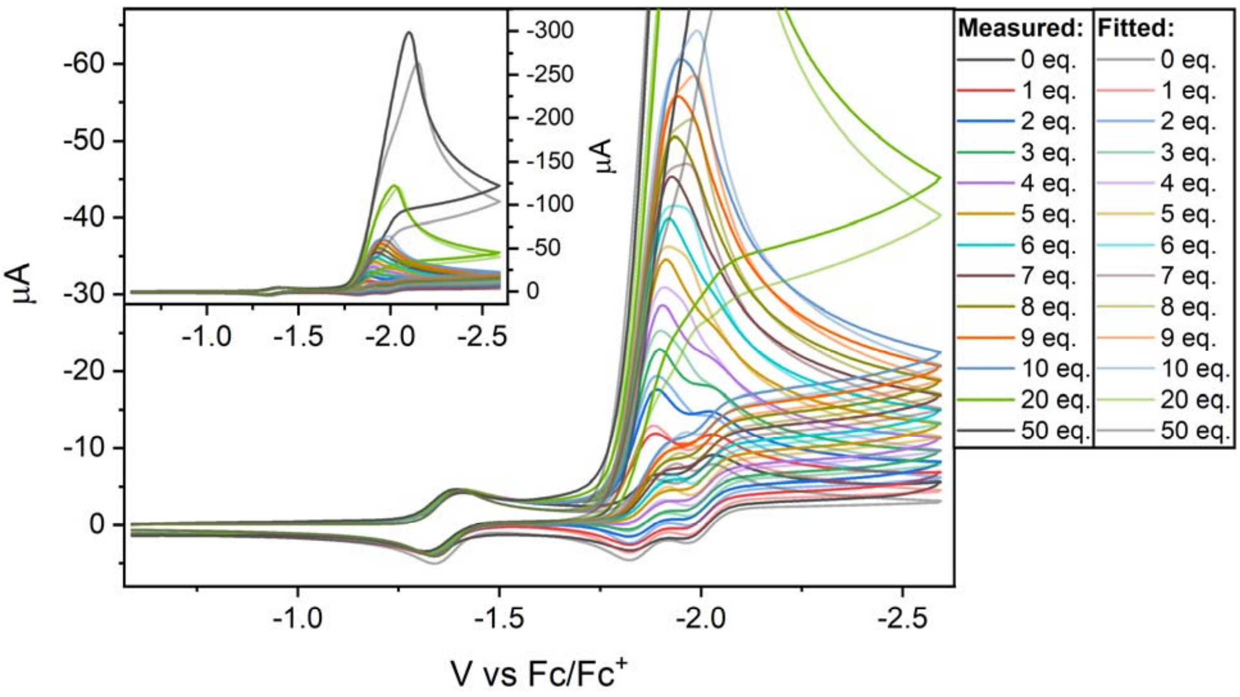

Figure SI8: Cyclic voltammogram of **R1** (1 mM) in DMF, upon addition of varying equivalents of $[HNEt_3](BF_4)$ (100 mM $TBAPF_6$) and fitting in DigiElch,[5-10] using the parameters in **Table SI6** and the mechanism shown in the main text.

Table SI6: Set of equations and parameters used for fitting of CV data of **R1** (1 mM) in DMF, upon addition of varying equivalents of $[HNEt_3](BF_4)$ (100 mM $TBAPF_6$) using DigiElch 8. D(**R1**) = 2.8E-6 cm^2/s, R_u = 600 Ω, C_{dl} = 3E-6 F, T = 298.5 K, electrode area S = 0.0314 cm^2, geometry planar, diffusion semi-infinite 1D. Fitted values are cursive, the two values with * are linked, fitted values are cursive. D(H_2) = 5E-5 and D($[HNEt_3](BF_4)$) = 8E-6 cm^2s^{-1} were used.[11-15]

Charge Transfer Reactions	$E_{1/2}$ (V vs Fc/Fc^+)	α	k_s (cm/s)
L-Co^{II} + e^- => L-Co^{I}	-1.37	0.5	10000
L-Co^{I} + e^- => L^{-1}-Co^{I}	-1.85	0.5	10000

L^{-1}-Co^{I} + e^{-} => L^{-2}-Co^{I}	-2.00	0.5	10000
L-Co^{II}-H + e^{-} => L-Co^{I}-H	-1.80*	0.5	10000
Chemical Reactions	K_{eq}	k_f	k_b
L^{-1}-Co^{I} + $HNEt_3^+$ => L-Co^{II}-H + NEt_3	8.16	12594	1543
L^{-2}-Co^{I} + $HNEt_3^+$ => L-Co^{I}-H + NEt_3	19578*	7.77E5	39.7
L-Co^{I}-H + $HNEt_3^+$ => L-Co^{I}-H_2 + NEt_3	0.023	1E9	4.3E10
L-Co^{I}-H_2 => L-Co^{I} + H_2	-	1E10	-

3.3 FOWA analysis of CV Data

For a ECEC, ECCE and EECC mechanism, with only one acid involved in the chemical step, the second reduction easier than first, $k_2 >> k_1$, $E_{cat/2}$ becomes $E_{1/2}$, we find at the foot of the wave:[16-19]

$$I = 2FSc_{cat}^0\sqrt{D_{cat}c_{acid}^0 k_1} \times \frac{1}{1+e^{\frac{F}{RT}(E-E_{cat/2})}}, \text{ in A} \qquad (1)$$

$$v_{H_2} = Sc_{cat}^0\sqrt{D_{cat}c_{acid}^0 k_1} \times \frac{1}{1+e^{\frac{F}{RT}(E-E_{cat/2})}}, \text{ in mol/s} \qquad (2)$$

Analogously, for a ECE'C, ECCE' and EE'CC mechanism (one electron transfer in solution), with only one acid involved in the chemical step, the second reduction easier than first, $k_2 >> k_1$, $E_{cat/2}$ becomes $E_{1/2}$, we find at the foot of the wave:

$$I = FSc_{cat}^0\sqrt{D_{cat}c_{acid}^0 2k_1} \times \frac{1}{1+e^{\frac{F}{RT}(E-E_{cat/2})}}, \text{ in A} \qquad (3)$$

$$v_{H_2} = Sc_{cat}^0\sqrt{\frac{D_{cat}c_{acid}^0 k_1}{2}} \times \frac{1}{1+e^{\frac{F}{RT}(E-E_{cat/2})}}, \text{ in mol/s} \qquad (4)$$

In the same case however, but with $k_1 \gg k_2$, $E_{cat/2}$ shifts anodically by $E_{cat/2} = E_{1/2} + \frac{RT}{F}\ln\left(\frac{k_1}{k_2}\right)$, and[17, 18]

$$I = 2FSc_{cat}^0\sqrt{D_{cat}c_{acid}^0 k_1} \times \frac{1}{1+e^{\frac{F}{RT}(E-E_{1/2})}}, \text{ in A} \qquad (5)$$

$$I = 2FSc_{cat}^0\sqrt{D_{cat}c_{acid}^0 k_2} \times \frac{1}{1+e^{\frac{F}{RT}(E-E_{cat/2})}}, \text{ in A} \qquad (6).$$

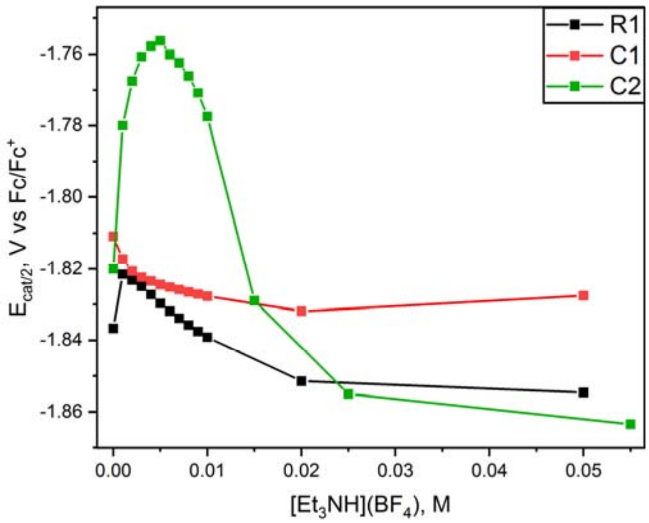

Figure SI9: Catalytic halve wave potentials extracted from CV data (**R1** and **C1** 1 mM, **C2** 0.55 mM; in DMF, 0.1 M $[TBA](PF_6)$; $[HNEt_3](BF_4)$ as indicated; glassy carbon working electrode (S = 0.0314 cm^2; R_u = 600 Ω; T = 300 K; 0.1 V s^{-1}).

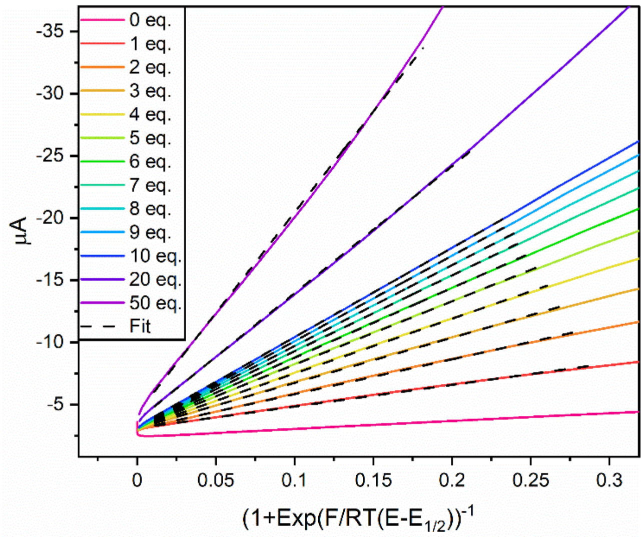

Figure SI10: FOWA of **R1** (1 mM) in DMF, upon addition of varying equivalents of $[HNEt_3](BF_4)$ (100 mM $TBAPF_6$) on $E_{1/2}$ = -1.85 V (S = 0.0314 cm^2; R_u = 600 Ω; T = 300 K; 0.1 V s^{-1}).

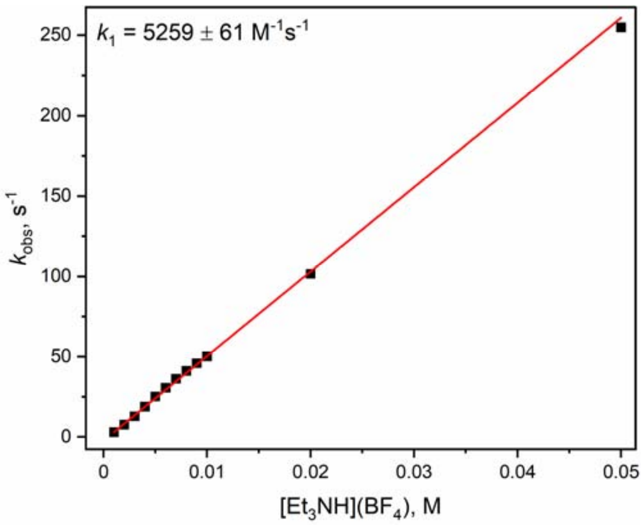

Figure SI11: Analysis of FOWA slopes for **R1** (1 mM) in DMF (**Figure SI10**), upon addition of varying equivalents of $[HNEt_3](BF_4)$ (100 mM $TBAPF_6$) on $E_{1/2}$ = -1.85 V, D = 2.8E-6 cm^2/s, R_u = 600 Ω, T = 300 K, electrode area S = 0.0314 cm^2.

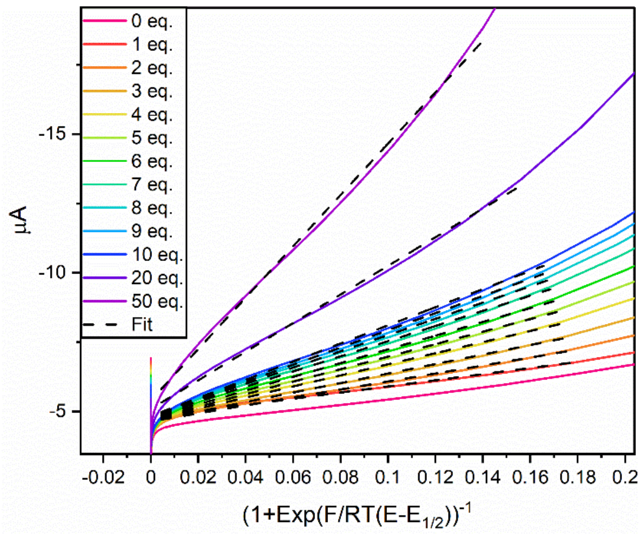

Figure SI12: FOWA of **C1** (1 mM) in DMF, upon addition of varying equivalents of $[HNEt_3](BF_4)$ (100 mM $TBAPF_6$) on $E_{1/2}$ = -1.83 V (S = 0.0314 cm^2; R_u = 600 Ω; T = 300 K; 0.1 V s^{-1}).

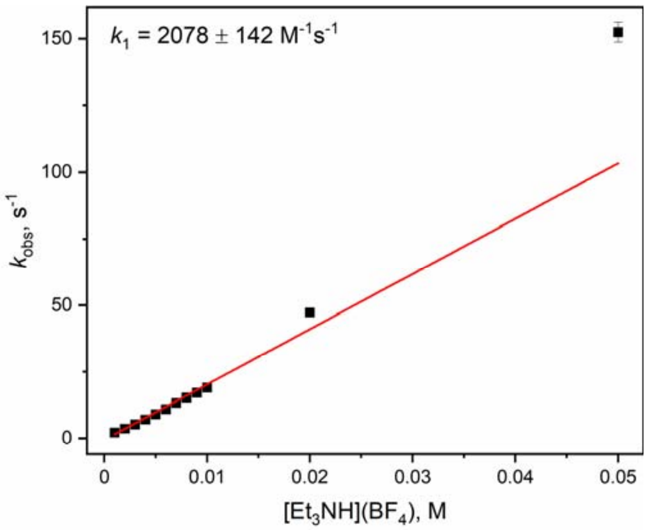

Figure SI13: Analysis of FOWA slopes for **C1** (1 mM) in DMF (**Figure SI12**), upon addition of varying equivalents of [$HNEt_3$](BF_4) (100 mM $TBAPF_6$) on $E_{1/2}$ = -1.83 V, D = 1.53E-6 cm^2/s, R_u = 600 Ω, T = 300 K, electrode area S = 0.0314 cm^2.

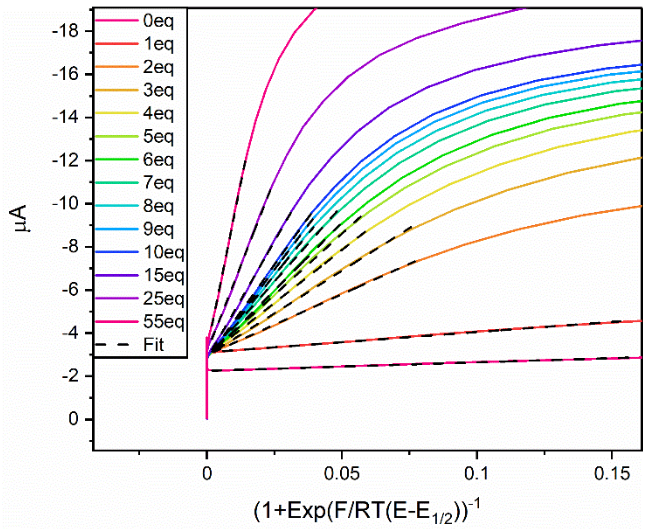

Figure SI14: FOWA of **C2** (0.55 mM) in DMF, upon addition of varying equivalents of [$HNEt_3$](BF_4) (100 mM $TBAPF_6$) on $E_{1/2}$ = -1.83 V (S = 0.0314 cm^2; R_u = 600 Ω; T = 300 K; 0.1 V s^{-1}).

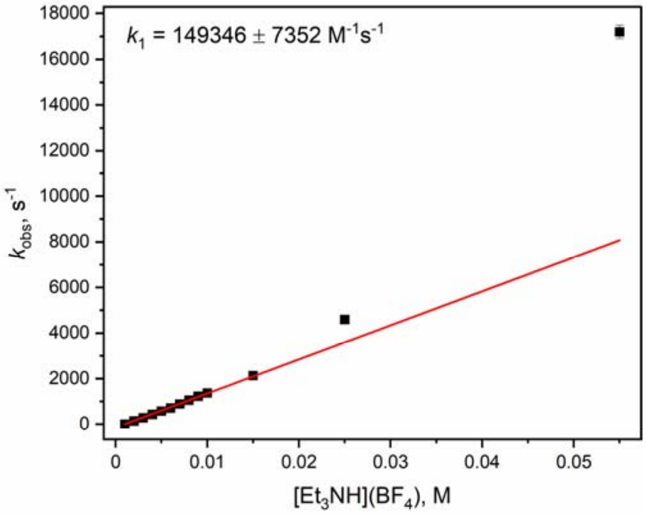

Figure SI15: Analysis of FOWA slopes for **C2** (0.55 mM) in DMF (**Figure SI14**), upon addition of varying equivalents of $[HNEt_3](BF_4)$ (100 mM $TBAPF_6$) on $E_{1/2}$ = -1.83 V, D = 2.0E-6 cm^2/s, R_u = 600 Ω, T = 300 K, electrode area S = 0.0314 cm^2.

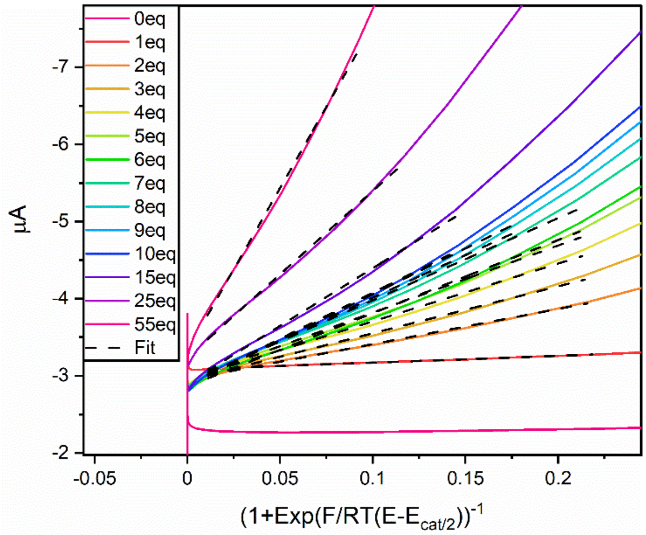

Figure SI16: FOWA of **C2** (0.55 mM) in DMF, upon addition of varying equivalents of $[HNEt_3](BF_4)$ (100 mM $TBAPF_6$) on $E_{cat/2}$ = -1.76 V (S = 0.0314 cm^2; R_u = 600 Ω; T = 300 K; 0.1 V s^{-1}).

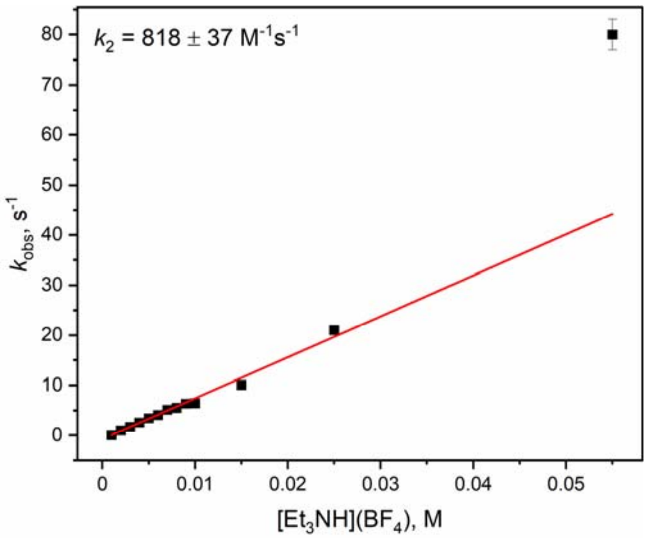

Figure SI17: Analysis of FOWA slopes for **C2** (0.55 mM) in DMF (**Figure SI16**), upon addition of varying equivalents of $[HNEt_3](BF_4)$ (100 mM $TBAPF_6$) on $E_{cat/2}$ = -1.76 V, D = 2.0E-6 cm^2/s, R_u = 600 Ω, T = 300 K, electrode area S = 0.0314 cm^2.

Table SI7: Summary of FOWA analysis of CV experiments, analysed according to Eq. 1. **R1** and **C1** 1 mM, **C2** 0.55 mM; in DMF, 0.1 M $[TBA](PF_6)$; 1 to 55 mM $[HNEt_3](BF_4)$; glassy carbon working electrode (S = 0.0314 cm^2); R_u = 600 Ω; T = 300 K; 0.1 V s^{-1}; **Figure SI9** - **Figure SI17**). Fitting to the transformed potential $\left(\mathbf{1}+\boldsymbol{Exp}(\boldsymbol{F}/\boldsymbol{RT}(\boldsymbol{E}-\boldsymbol{E}_{\boldsymbol{cat}/\mathbf{2}}))\right)^{-\mathbf{1}}$ was performed in the range of 10 µA, to prevent deviations due to acid depletion and decomposition.

	D cm^2/s	$E_{1/2}$, V	$E_{cat/2}$, V	k_1, $M^{-1}s^{-1}$	k_2, $M^{-1}s^{-1}$	Mechanism
**R1**	2.8E-6	-1.85	-1.85	5.3±0.1E3	k1 ≪	(E)ECEC
**C1**	1.53E-6	-1.83	-1.83	2.1±0.1E3	k1 ≪	(EE)ECEC
**C2**	2.0E-6	-1.83	-1.76	1.5±0.1E5	8.2±0.4E2	(E)EECC
**C2**	2.0E-6	-1.83	-1.83	1.5±0.1E5	k1 ≪	(EE)ECEC

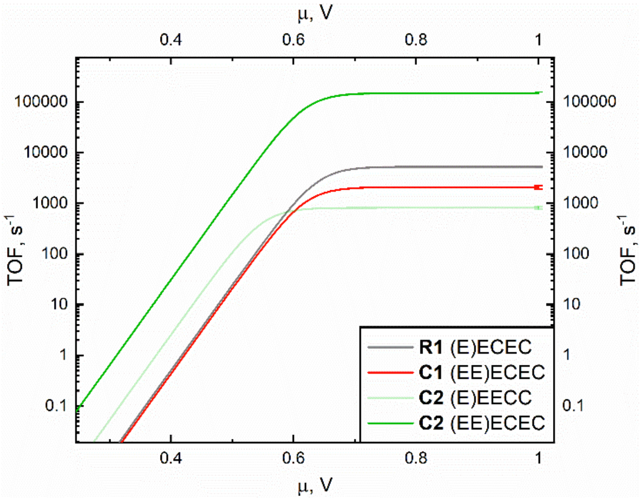

Figure SI18: Catalytic Tafel Plots for all three catalysts at 1 M [Et_3NH](BF_4), based on CV data from **Table SI7.**

3.4 LSV Experiments

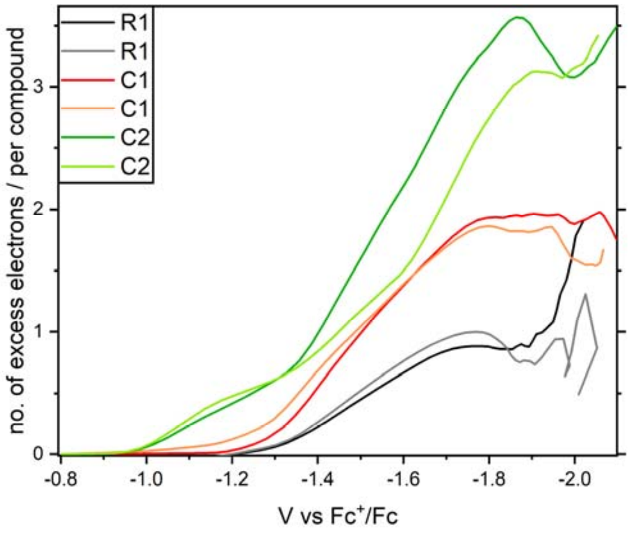

Figure SI19: Electron balance in electron equivalents per compound in LSV experiments. [$HNEt_3$](BF_4) served as proton source, inline H_2 monitoring was conducted. Curves represent two independent runs per compound. All data collected on an Hg pool, in 5 ml DMF, 0.1 M $TBAPF_6$, 0.4 mM catalyst, 200 mM [$HNEt_3$](BF_4), scan rate 0.1 mV s^{-1}, $R_u \approx 70\ \Omega$, T = 300 K, electrode area S = 1.3±0.1 cm^2.

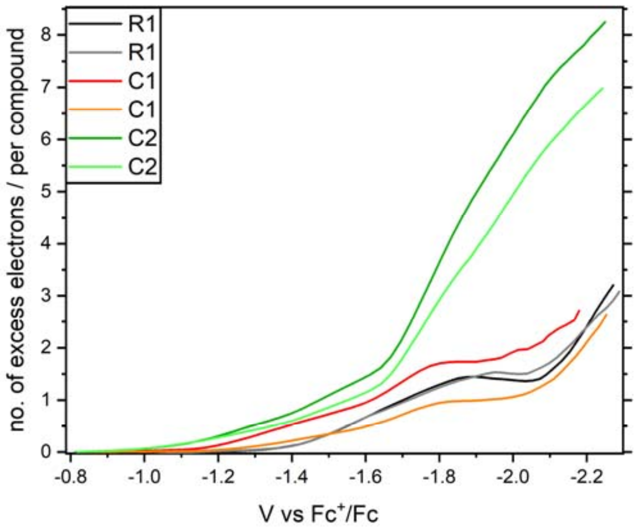

Figure SI20: Electron balance in electron equivalents per compound in LSV experiments. AcOH served as proton source, inline H_2 monitoring was conducted. Curves represent two independent runs per compound. All data collected on an Hg pool, in 5 ml DMF, 0.1 M $TBAPF_6$, 0.4 mM catalyst, 200 mM AcOH, scan rate 0.1 mV s^{-1}, $R_u \approx 70\ \Omega$, T = 300 K, electrode area S = 1.3±0.1 cm^2.

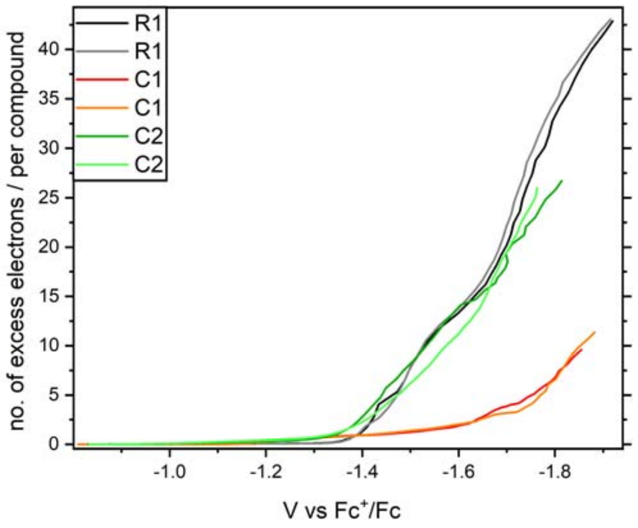

Figure SI21: Electron balance in electron equivalents per compound in LSV experiments. H(BF4) served as proton source, inline H_2 monitoring was conducted. Curves represent two independent runs per compound. All data collected on an Hg pool, in 5 ml DMF, 0.1 M $TBAPF_6$, 0.4 mM catalyst, 200 mM $H(BF_4)$, scan rate 0.1 mV s^{-1}, $R_u \approx 70\ \Omega$, T = 300 K, electrode area S = 1.3±0.1 cm^2.

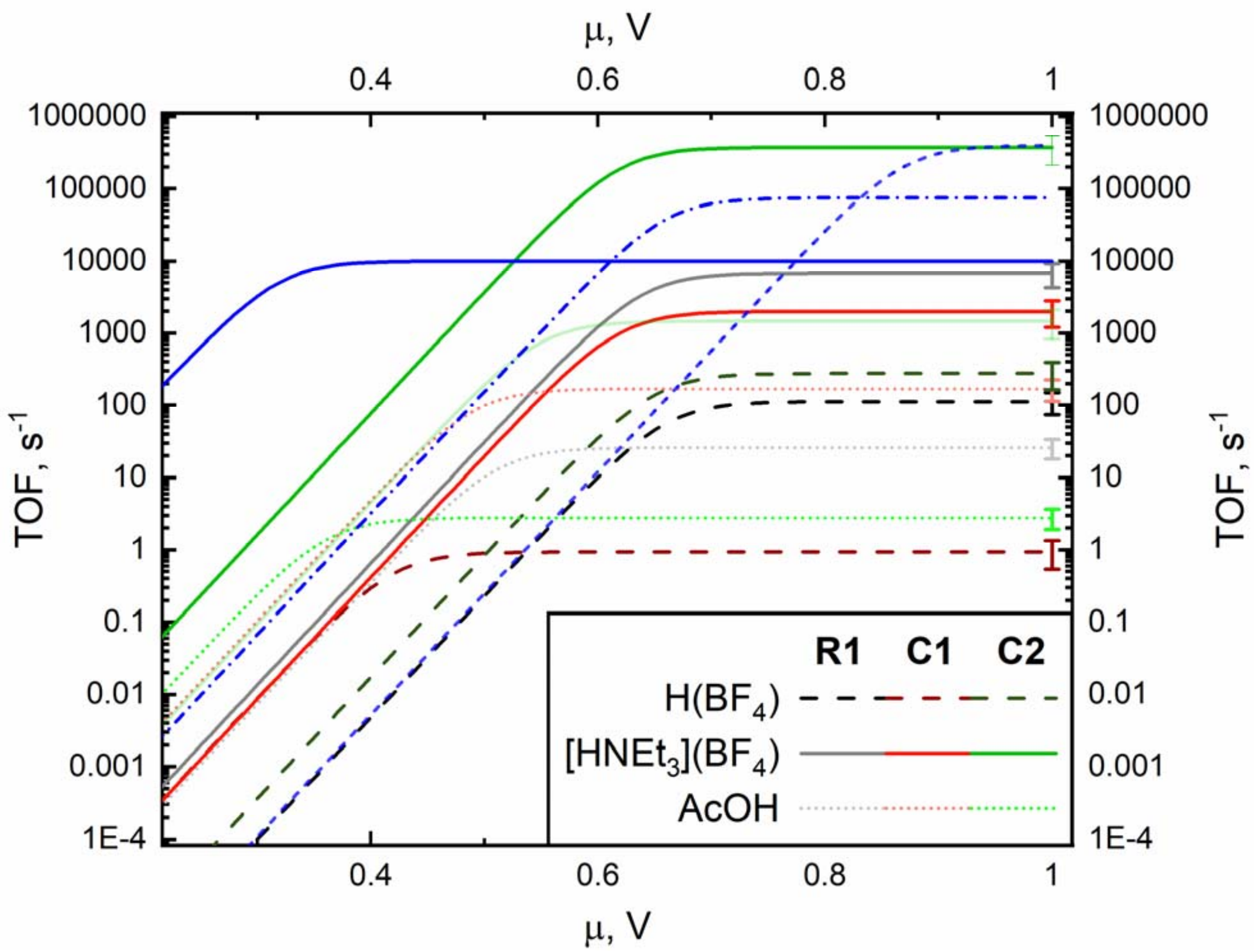

Figure SI22: Catalytic Tafel Plots for all three catalysts and all three acids (1 M) discussed in this work (based on LSV data from **Table SI8**). For **C2** and $[HNEt_3](BF_4)$ both the (E)EECC (solid shaded green line) and (EE)ECEC (solid green line) mechanisms are displayed. The solid blue line is $[Co^{II}(dmgH)_2py]$ using $[HNEt_3]^+$, the dash dotted blue line is $[Ni^{II}(P_2^{Ph}N^{Ph})_2]^{2+}$ using $[HDMF]^+$ and the short dashed blue line is $[Fe^{II}TPP]$ using $[HNEt_3]^+$.[13, 20, 21]

Table SI8: Summary of FOWA analysis of all the LSV experiments. H_2 formation rates measured by GC/TCD were correlated with the respective currents, and transferred according to Eq. 2. All data collected on an Hg pool, in 5 ml DMF, 0.1 M $TBAPF_6$, 0.4 mM catalyst, 200 mM proton source, scan rate 0.1 mV s^{-1}. Diffusion coefficients from CV data (**Figure SI5** - **Figure SI7**), $R_u \approx 70\ \Omega$, T = 300 K, electrode area S = 1.3±0.1 cm^2. Fitting to the transformed potential $\left(\mathbf{1} + \boldsymbol{Exp}(\boldsymbol{F}/\boldsymbol{RT}(\boldsymbol{E} - \boldsymbol{E_{cat/2}}))\right)^{-\mathbf{1}}$ was performed in the range of 1 nmol/s, to prevent deviations due to acid depletion and decomposition.

Acid, pK_a	$H(BF_4)$, 3.4				$[HNEt_3](BF_4)$, 9.2				AcOH, 13.5			
	$E_{1/2}$, V	$E_{cat/2}$, V	k_1, $M^{-1}s^{-1}$	k_2, $M^{-1}s^{-1}$	$E_{1/2}$, V	$E_{cat/2}$, V	k_1, $M^{-1}s^{-1}$	k_2, $M^{-1}s^{-1}$	$E_{1/2}$, V	$E_{cat/2}$, V	k_1, $M^{-1}s^{-1}$	k_2, $M^{-1}s^{-1}$
**R1**	-1.37	-1.37	1.1 ±0.4E2	$k_1 \ll$	-1.85	-1.85	6.8 ±2.5E3	$k_1 \ll$	-1.85	-1.85	26±8	$k_1 \ll$
**C1**	-1.13	-1.13	9.3 ±3.9E-1	$k_1 \ll$	-1.83	-1.83	2.0 ±0.8E3	$k_1 \ll$	-1.83	-1.83	1.7 ±0.6E2	$k_1 \ll$
**C2**	-1.36	-1.36	2.8 ±1.1E2	$k_1 \ll$	-1.83	-1.76	3.7 ±1.6E5	1.5 ±0.6E3	-1.83	-1.70	6.3 ±2.4E2	2.8±0.9

Table SI9: Overall faradic efficiencies for LSV experiments with inline H_2 detection (**Figure 3** and **Figure SI23** - **Figure SI25**). All data collected on an Hg pool, electrode area S = 1.3±0.1 cm2, in 5 ml DMF, 0.1 M $TBAPF_6$, 0.4 mM catalyst, 200 mM proton source, scan rate 0.1 mV s^{-1}.

Compound	Proton Source	Faradaic Efficiency, %	Error, %
**R1**	AcOH	87	1.7
	$[HNEt_3](BF_4)$	97	1.9
	$H(BF_4)$	83	1.6
**C1**	AcOH	80	1.6
	$[HNEt_3](BF_4)$	95	1.8
	$H(BF_4)$	86	1.6
**C2**	AcOH	89	1.6
	$[HNEt_3](BF_4)$	93	1.8
	$H(BF_4)$	80	1.5

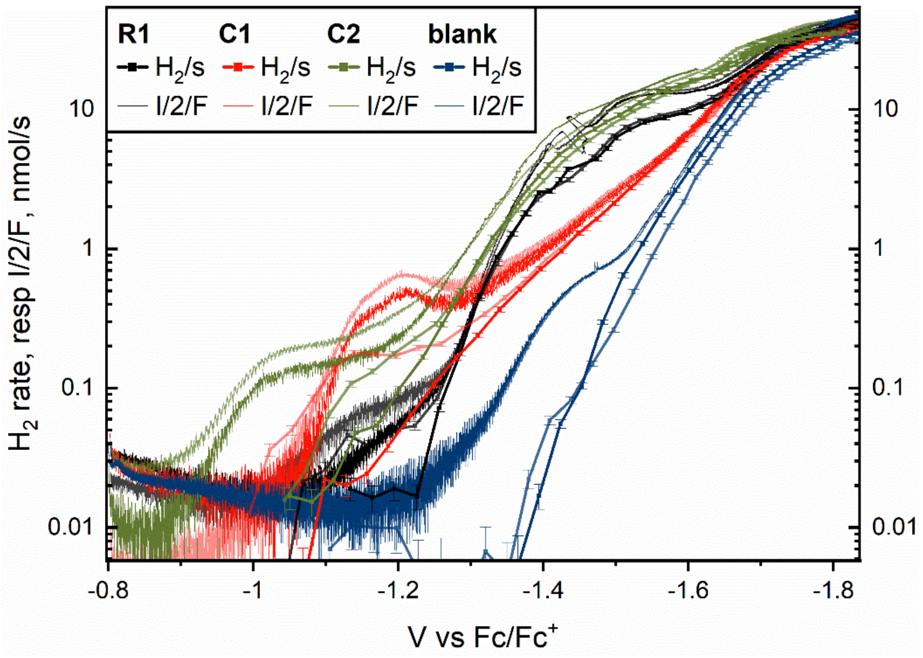

Figure SI23: LSV with H(BF4) as proton source with in line H_2 formation rates measured by GC/TCD. H_2 formation rates measured by GC/TCD were correlated with the respective currents (I/2/F). All data collected on an Hg pool, in 5 ml DMF, 0.1 M $TBAPF_6$, 0.4 mM catalyst, 200 mM $H(BF_4)$, scan rate 0.1 mV s^{-1}, $R_u \approx 70\ \Omega$, T = 300 K, electrode area S = 1.3±0.1 cm^2.

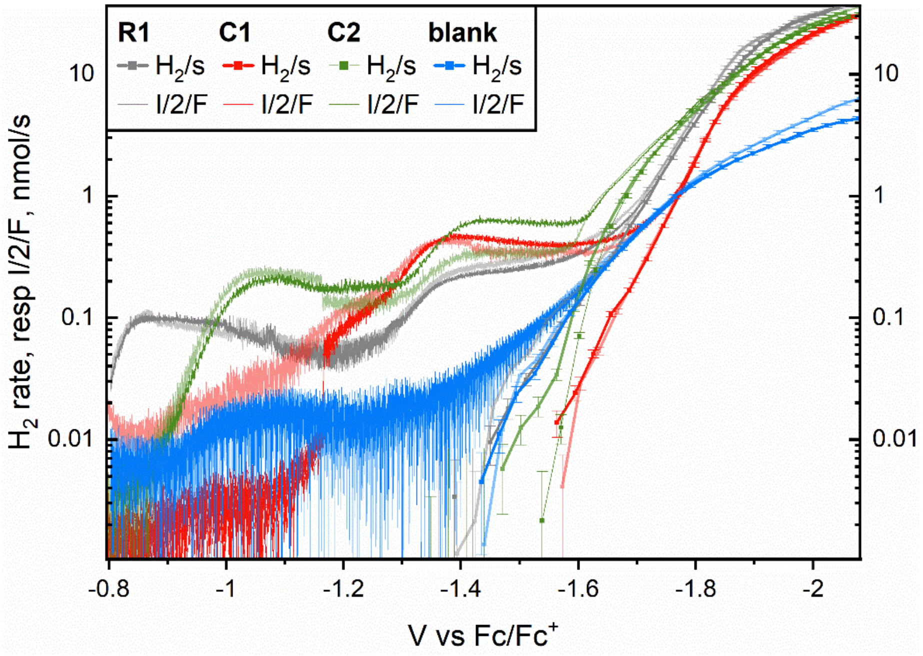

Figure SI24: LSV with $[HNEt_3](BF_4)$, as proton source with in line H_2 formation rates measured by GC/TCD. H_2 formation rates measured by GC/TCD were correlated with the respective currents (I/2/F). All data collected on an Hg pool, in 5 ml DMF, 0.1 M $TBAPF_6$, 0.4 mM catalyst, 200 mM $[HNEt_3](BF_4)$, scan rate 0.1 mV s^{-1}, $R_u \approx 70\ \Omega$, T = 300 K, electrode area S = 1.3±0.1 cm^2.

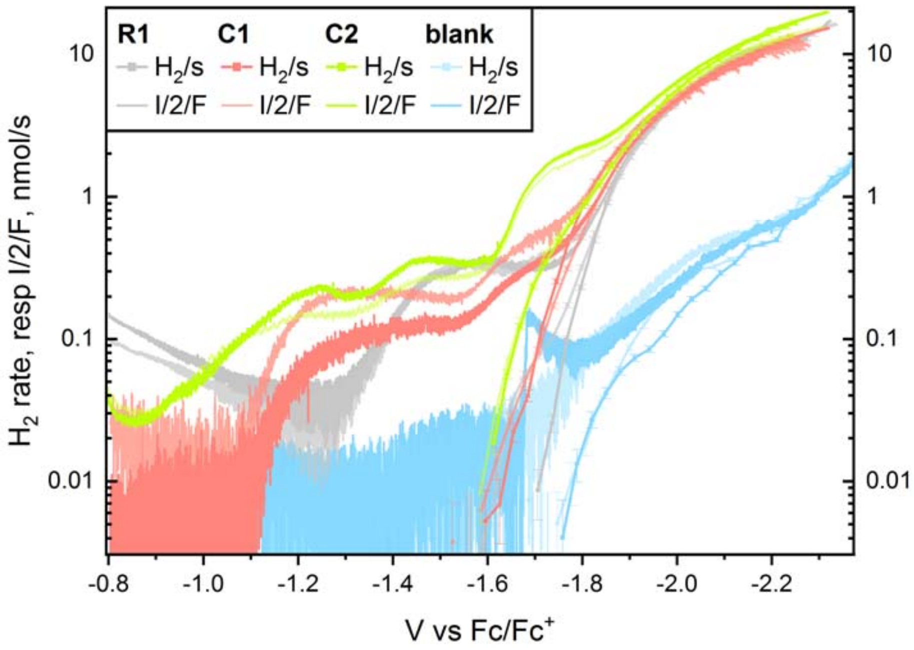

Figure SI25: LSV with AcOH as proton source with in line H_2 formation rates measured by GC/TCD. H_2 formation rates measured by GC/TCD were correlated with the respective currents (I/2/F). All data collected on an Hg pool, in 5 ml DMF, 0.1 M TBAPF_6, 0.4 mM catalyst, 200 mM AcOH, scan rate 0.1 mV s^{-1}, $R_u \approx 70\ \Omega$, T = 300 K, electrode area S = 1.3±0.1 cm^2.

3.5 CA Experiments

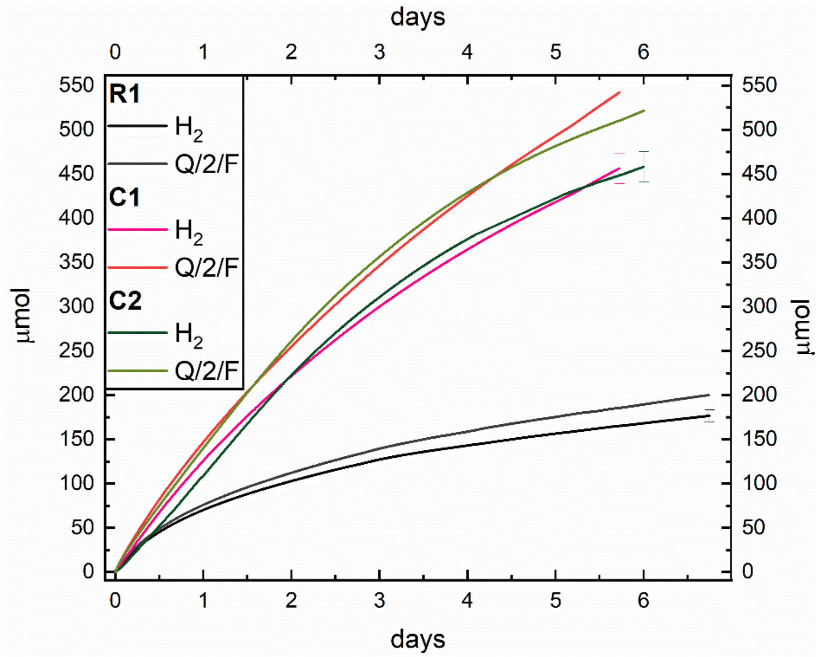

Figure SI26: Chronoamperometry: 400 µM **R1**, **C1** and **C2**, 200 mM $[HNEt_3](BF_4)$, 0.1 M $[TBA](PF_6)$, 5 ml, DMF, WE is a Hg pool, RE is Ag/AgCl, V is given vs Fc/Fc^+, AE is Pt separated by a glass frit.

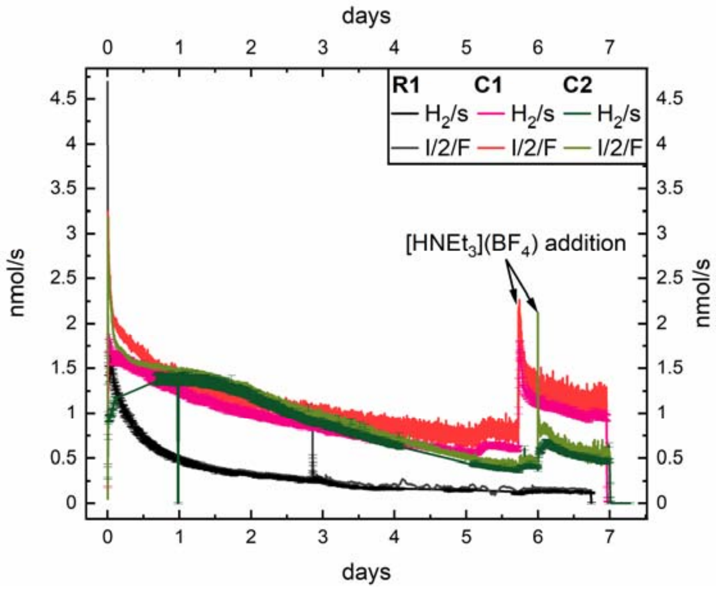

Figure SI27: Chronoamperometry: 400 µM **R1**, **C1** and **C2**, 200 mM $[HNEt_3](BF_4)$, 0.1 M $[TBA](PF_6)$, 5 ml, DMF, WE is a Hg pool, RE is Ag/AgCl, V is given vs Fc/Fc^+, AE is Pt separated by a glass frit. In the case of **C1**, $[HNEt_3](BF_4)$ (0.55 mmol) was added after 5.75 days and for the **C2** experiment 0.4 mmol were added after 6 days.

4 Photocatalytic Experiments in H_2O

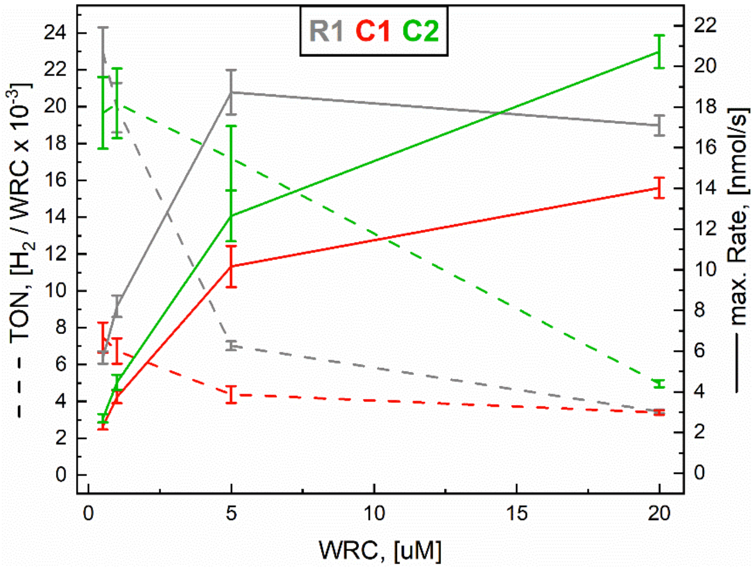

Figure SI28: Photocatalytic measurements with **R1**, **C1** and **C2** were conducted in H_2O at pH 5, with 0.1 M NaAsc and 0.1 M tris(carboxyethyl)phosphine as electron relay and sacrificial electron donor, respectively, 500 µM $[Ru(bipyridine)_3]Cl_2$ as photosensitizer, illumination at 450 nm, total volume 10 mL.

5 References

1. R. C. Clark and J. S. Reid, *Acta Cryst.*, 1995, **51**, 887-898.
2. O. V. Dolomanov, L. J. Bourhis, R. J. Gildea, J. A. K. Howard and H. Puschmann, *J. Appl. Cryst.*, 2009, **42**, 339-341.
3. G. M. Sheldrick, 2015, **Acta Cryst.**, 3-8.
4. G. M. Sheldrick, *Acta Cryst.*, 2015, **C71**, 3-8.
5. M. Rudolph, *J. Electroanal. Chem.*, 1992, **338**, 85-98.
6. M. Rudolph, *J. Electroanal. Chem.*, 1994, **375**, 89-99.
7. M. Rudolph, *J. Electroanal. Chem.*, 2001, **503**, 15-27.
8. M. Rudolph, *J. Electroanal. Chem.*, 2002, **529**, 97-108.
9. M. Rudolph, *J. Electroanal. Chem.*, 2003, **543**, 23-39.
10. M. Rudolph, *J. Electroanal. Chem.*, 2004, **571**, 289-307.
11. K. Sporka, J. Hanika, V. Ruzicka and M. Halousek, *Collect. Czech. Chem. Commun.*, 1971, **36**, 2130-2136.
12. I. M. Krieger, G. W. Mulholland and C. S. Dickey, *J. Phys. Chem.*, 1967, **71**, 1123-+.
13. M. Razavet, V. Artero and M. Fontecave, *Inorg. Chem.*, 2005, **44**, 4786-4795.
14. V. Fourmond, P. A. Jacques, M. Fontecave and V. Artero, *Inorg. Chem.*, 2010, **49**, 10338-10347.
15. X. L. Hu, B. S. Brunschwig and J. C. Peters, *J. Am. Chem. Soc.*, 2007, **129**, 8988-8998.
16. N. Queyriaux, D. Sun, J. Fize, J. Pecaut, M. J. Field, M. Chavarot-Kerlidou and V. Artero, *J. Am. Chem. Soc.*, 2020, **142**, 274-282.
17. E. S. Rountree, B. D. McCarthy, T. T. Eisenhart and J. L. Dempsey, *Inorg. Chem.*, 2014, **53**, 9983-10002.
18. C. Costentin and J. M. Saveant, *ChemElectroChem*, 2014, **1**, 1226-1236.
19. C. Costentin, S. Drouet, M. Robert and J. M. Saveant, *J. Am. Chem. Soc.*, 2012, **134**, 11235-11242.
20. V. Artero and J. M. Saveant, *Energ. Environ. Sci.*, 2014, **7**, 3808-3814.
21. I. Bhugun, D. Lexa and J. M. Saveant, *J. Am. Chem. Soc.*, 1996, **118**, 3982-3983.

6 Computational Details

6.1 Geometry Optimization and Single-Point Calculation

All Kohn-Sham DFT calculations were performed using Gaussian 16[1] with NVIDIA P100 GPU acceleration on Pitz Daint supercomputer. Initial structure of ferrocene (Fc), ferricinium (Fc^+), **R1**, **C1** and **C2** complexes were extracted from X-ray crystal structure. Geometry optimizations of all species including oxidized and reduced ones were carried out in the gas phase with the B3LYP exchange-correlation functional. All atoms were treated with 6-31G(d) basis set, except the cobalt atoms which were treated with the Stuttgart-Dresden relativistic (SDD) effective core potential (ECP). The vibrational frequency calculations were carried out at the same level of theory to to calculate Gibbs free energies assuming a temperature of 298.15 K. Solvation effects were approximately included by the polarizable continuum model (PCM) with dielectric constant of 37.219 to define the N,N-dimethylformamide (DMF) phase.

6.2 Reduction Potential Calculations

Reduction potential E_{re} as used in this work is defined as the difference between absolute reduction potential (E_{abs}) and reference potential E_{ref}

$$E_{re} = E_{abs} - E_{ref} \tag{1}$$

with

$$E_{abs} = -\frac{\Delta G}{nF} \tag{2}$$

where ΔG is the change of Gibbs free energy of the reduction reaction, n is the number of electrons, and F is Faraday constant (23.06 kcal.mol^{-1}.V^{-1}). In our study the computed Fc^+/Fc electrode potential $E_{Fc^+/Fc}$ is used as reference potential.

Reduction potentials were computed from the gas phase and solvation Gibbs free energies following Born-Haber (BH) cycle.[2, 3] The estimation of E_{re} using the BH cycle was also discussed in our previous work.[4]

7 Studied System

Table 1: Charges and spin multiplicities of cobalt for C1 and C2 complexes.

**Charge of cobalt**	**Charge of dimer complex**	**Spin multiplicity**
2+	4+	Septet
1+	2+	Quintet
0	0	Triplet

Combinations of charge and spin multiplicity of Co(II), Co(I), and Co(0) atoms considered in the calculations is reported in Table 1.

7.1 Cobalt Complexes

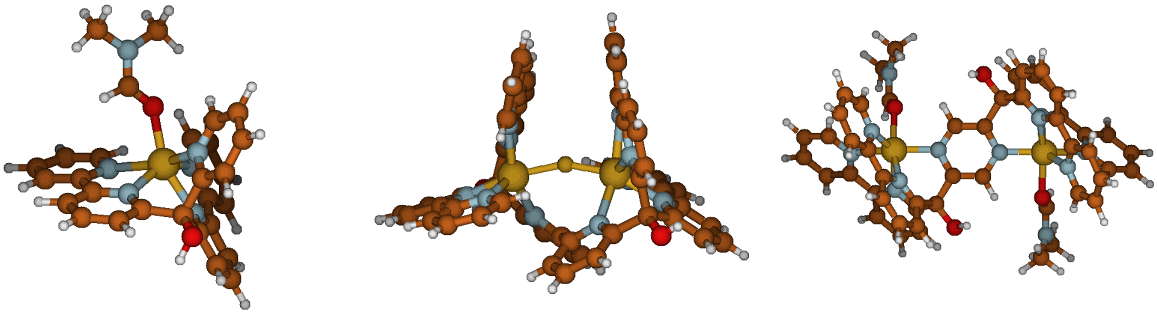

(a) R1 (b) C1 (c) C2

Figure 1: Optimized structures of mononuclear **R1** and dinuclear **C1** and **C2** at DFT/B3LYP/6-31G(d)/SDD-ECP in DMF.

7.2 Ferrocene

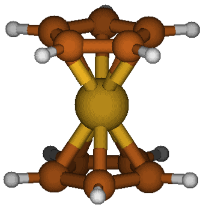

Figure 2: Optimized structures of ferrocene at DFT/B3LYP/6-31G(d)/SDD-ECP in DMF.

8 Computational Models of Dinuclear Complexes

8.1 Reduction Models for C1 and C2

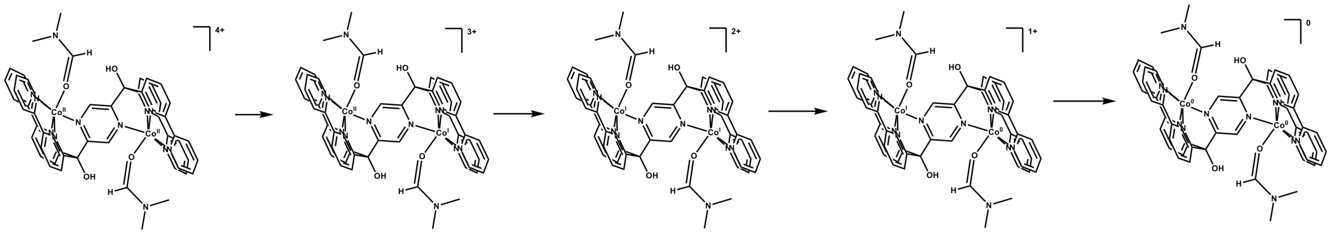

(a) Model 1: No changes to the octahedral ligand field.

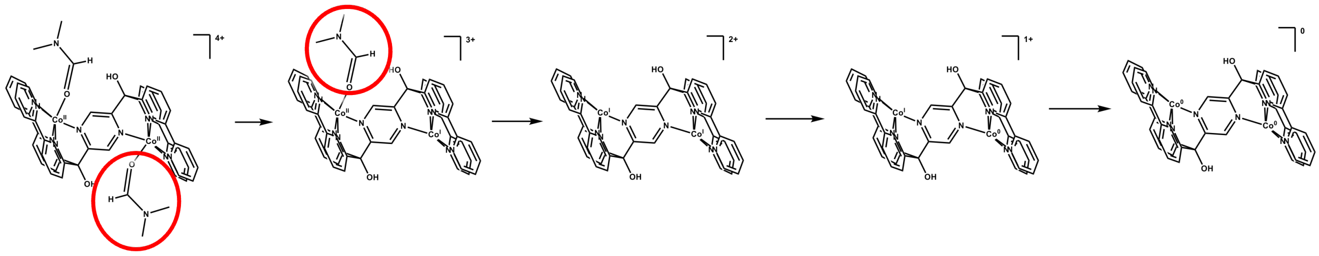

(b) Model 2: DMF is lost upon reduction to Co(I) (in two consecutive steps).

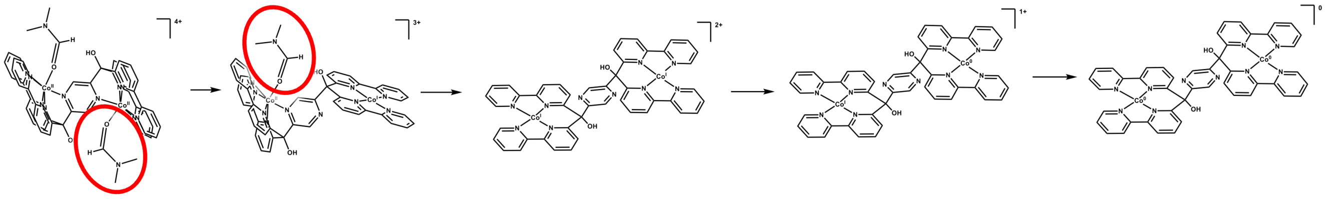

(c) Model 3: DMF and pyrazine de-coordinate upon reduction to Co(I) to give a square structure.

Figure 3: Models for structural change of complexes **C1** and **C2**.

8.2 Proposed DMF Dissociation Models

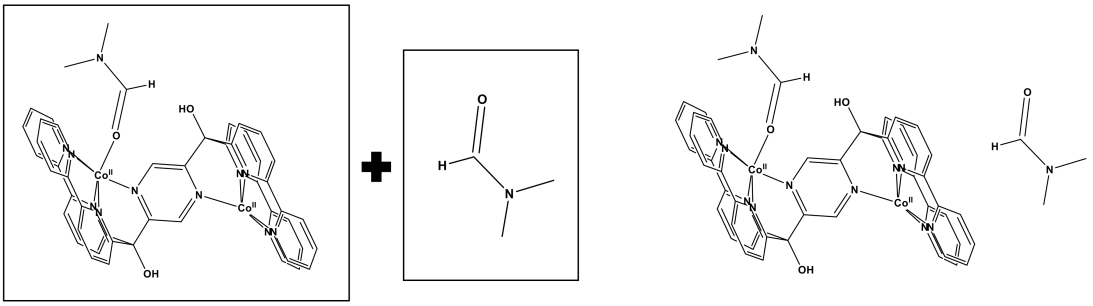

$E_{total} = E_{Co\text{-}Co} + E_{DMF}$

(a) Model A: Separation model

$E_{total} = E_{Co\text{-}Co\text{---}DMF}$

(b) Model B: Association model

Figure 4: Proposed models for DMF dissociation for complexes **C1** and **C2**. Model A: In the separation model, the DMF and metal complex molecule are independent of each other. Model B: In the association model, a DMF molecule surrounds a metal complex.

We propose in this work two models (Model A and Model B) for modeling the dissociation of DMF in the reduction-based change for the complexes **C1** and **C2**. These two models are associated with the Model 2 and Model 3 as the two DMF molecules are sequentially lost upon the first and the second reduction of the dinuclear complex, respectively.

9 Computed Reduction Potentials

Table 2: Computed reduction potentials (in V) for the first 4 reductions.

Complex	Model	Change of total charge			
		4+ → 3+	3+ → 2+	2+ → 1+	1+ → 0
**R1**	1	-	-	-1.309	-1.188
	2A	-	-	-1.346	-1.750
	2B	-	-	-1.327	-1.424
**C1**	1	-1.120	-1.255	-1.884	-2.559
	2	-1.186	-1.672	-2.743	-2.745
	3A	-1.120	-1.255	-1.884	-2.559
	3B	-1.160	-1.701	-2.063	-2.127
**C2**	1	-1.106	-1.204	-1.986	-2.119
	2A	-1.302	-1.654	-2.246	-2.580
	2B	-1.060	-1.396	-1.846	-2.000
	3	-1.338	-1.630	-2.211	-2.545

The computed reduction potentials of **R1**, **C1** and **C2** are listed in Table 2. We applied the proposed reduction model (Model 1, 2, and 3) to all complexes except of the **R1** complex as it shows only two reductions and there is no structural transformation to a square-planar form elucidated by experiments.

For the **C1** complex with the Model 1, the reduction potentials clearly show that charges of both cobalt atoms gradually decrease and the electron is distributed equally over two cobalt atoms, whereas in the Model 2 cobalt atoms gain electrons for the first and third reductions.

In the case of the **C2** complex, we found different behaviors of the complex and distinct changes of its electronic structure in four different models: (1) Model 1: a stack of electrons to cobalt atoms is detected after the second reduction, (2) Model 2A: atomic charge fo the cobalt atoms slightly decreased after the first and second reductions, indicating that most of the reducing electron transfers from the metals to the ligand. We also found that the charge of the cobalt atom decreased for the third reaction which is in contrast to previous reduction steps, confirming electron transfer to the cobalt atoms, (3) Model 2B: we found the same change of charge as in Model 2A and additionally there is no electron distribution to cobalt atoms for the fourth reduction, (4) Model 3: for the first reduction (one side of the complex is destructured to a square-planar shape), electrons hop to the cobalt atom on the destructured side compared to the other, and electrons are distributed equally to both cobalt atoms for the third and fourth reductions.

However, we note here that Mulliken charge depends significantly on the atomic orbitals (basis set) chosen for the calculation and do not have a well-defined complete basis set limit.

Delocalized MOs, localized natural bond orbitals (NBOs) and Boys orbitals (BOs) are provided in the section 12.

Table 3: Computed reduction potentials (in V) of different **C2** conformers of Model 2B for the first 4 reductions. Percent relative range is computed using maximum and minimum computed reduction potentials.

**Conformer**	**Change of total charge**			
	**4+ → 3+**	**3+ → 2+**	**2+ → 1+**	**1+ → 0**
1	-1.060	-1.396	-1.846	-2.000
2	-1.044	-1.366	-1.802	-2.005
3	-1.043	-1.398	-1.789	-1.995
Average	-1.049	-1.386	-1.812	-2.005
Percent relative range	± 1.6%	± 1.9%	± 3.1%	± 0.4%

We investigated the effect of different placements of a DMF molecule around the **C2** complex. Three conformers were modeled with four reductions. Table 3 reports the DFT-computed reduction potentials as a function of change of total charge for **C2** for different conformers. As can be seen in the table, the reduction potentials of all conformers are very close.

10 Population Analysis

Table 4: Mulliken charge (in atomic charge unit) of the **C1** and **C2** complexes computed at DFT/B3LYP/6-31G(d)/SDD-ECP level of theory.

**Complex**	**Model**	**Metal**	**non-red**	**1st red**	**2nd red**	**3rd red**	**4th red**
**C1**	1	Co_1	0.579	0.498	0.431	0.409	0.394
		Co_2	0.580	0.511	0.425	0.399	0.395
**C1**	2	Co_1	0.579	0.510	0.489	0.417	0.387
		Co_2	0.580	0.521	0.487	0.405	0.385
**C2**	1	Co_1	0.611	0.567	0.479	0.450	0.445
		Co_2	0.611	0.567	0.479	0.450	0.397
**C2**	2A	Co_1	0.611	0.585	0.485	0.435	0.401
		Co_2	0.611	0.590	0.485	0.436	0.410
**C2**	2B	Co_1	0.611	0.560	0.490	0.466	0.466
		Co_2	0.611	0.574	0.498	0.492	0.492
**C2**	3	Co_1	0.611	0.576	0.496	0.477	0.472
		Co_2	0.611	0.517	0.491	0.477	0.472

Table 5: Löwdin charge (in atomic charge unit) of the **C1** and **C2** complexes computed at DFT/B3LYP/6-31G(d)/SDD-ECP level of theory.

**Complex**	**Model**	**Metal**	**non-red**	**1st red**	**2nd red**	**3rd red**	**4th red**
**C1**	1	Co_1	0.623	0.535	0.464	0.439	0.423
		Co_2	0.630	0.556	0.462	0.438	0.429
**C1**	2	Co_1	0.623	0.539	0.517	0.441	0.409
		Co_2	0.630	0.554	0.518	0.430	0.409
**C2**	1	Co_1	0.608	0.564	0.477	0.448	0.443
		Co_2	0.608	0.572	0.484	0.454	0.449
**C2**	2A	Co_1	0.608	0.576	0.487	0.457	0.403
		Co_2	0.608	0.594	0.492	0.442	0.407
**C2**	2B	Co_1	0.608	0.567	0.485	0.472	0.472
		Co_2	0.608	0.579	0.496	0.487	0.487
**C2**	3	Co_1	0.608	0.552	0.475	0.457	0.453
		Co_2	0.608	0.496	0.471	0.457	0.453

11 Intramolecular Electron Transfer

To investigate the feasibility of our proposed electron transfer reaction mechanism in dinuclear Co(II)-Co(II), we adopt Marcus theory to calculate the rate of intramolecular electron transfer (IET).[5] Electronic communication between two adiabatic states A and B (ϕ_1 and ϕ_2) can be quantified by ET matrix element (coupling) H_{AB}[6] (see Tables 6 and 7). Generally, the rate of ET is directly proportional to H_{AB}.

Constrained DFT calculations were carried out at the same level of theory used in the calculation of reduction potential, using NWChem[7] in combination with the calculation of H_{AB} using in-house code based on the implementation described in a previous publication.[8]

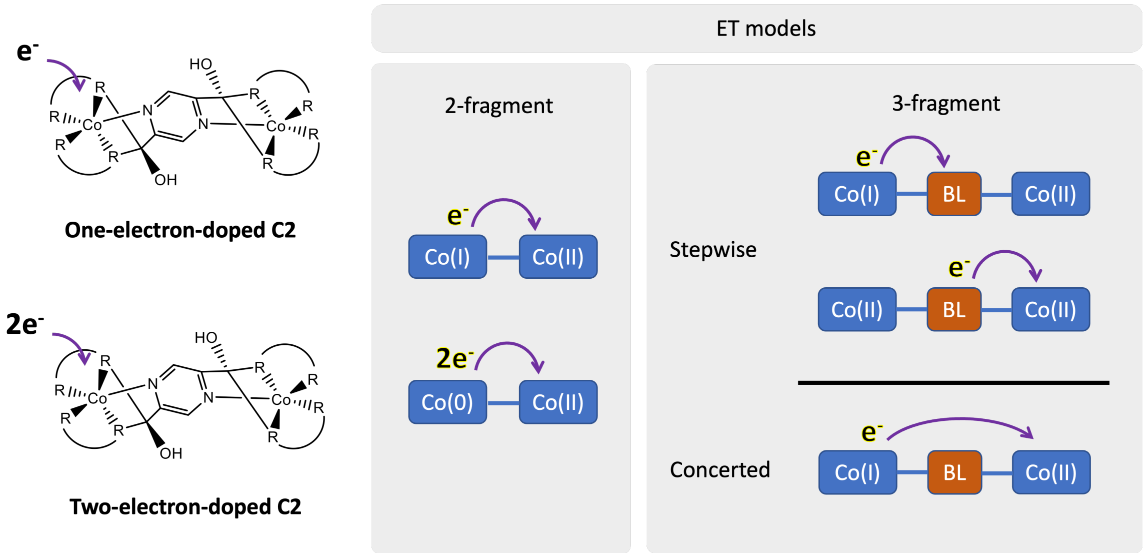

Figure 5: 2-fragment and 3-fragment models for intramolecular electron transfer in the dinuclear **C2** complex. Pyrazine is a bridging ligand (BL).

Figure 5 displays a graphical scheme of two models, 2-fragment and 3-fragment, that are used to simulate stepwise and concerted IET. Regions (part of the molecule) are divided into two and three fictitious fragments to construct the so-called *before* and *after* electron transfer fragments, which refer to the first and second cobalt atoms being a host of the electron as donor and acceptor, respectively.

Table 6: Computed H_{AB} of one electron transfer (ET) in dinuclear **C1** and **C2** complexes. Energies are in kcal mol^{-1}.

Complex	Before ET	After ET	H_{AB}
**C1**	Co(I)-Co(II)	Co(II)-Co(I)	0.013
**C2**	Co(I)-Co(II)	Co(II)-Co(I)	0.604

Table 7: Comparison of computed H_{AB} between stepwise and concerted two electron transfer (ET) mechanisms in a dinuclear C2 complex. BL is bridging ligand. Energies are in kcal mol^{-1}.

**Degree of ET**	**Model**	**Step**	**Situation**		$\mathbf{H}_{AB}$
			**Before ET**	**After ET**	
1 electron	2 fragments	Concerted	Co(I)-Co(II)	Co(II)-Co(I)	0.604
	3 fragments	Stepwise	Co(I)-BL(0)-Co(II)	Co(II)-BL(-1)-Co(II)	0.123
			Co(II)-BL(-1)-Co(II)	Co(II)-BL(0)-Co(I)	0.053
		Concerted	Co(I)-BL(0)-Co(II)	Co(II)-BL(-0)-Co(I)	2.902
2 electrons	2 fragments	Concerted	Co(0)-Co(II)	Co(II)-Co(0)	0.016
	3 fragments		Co(0)-BL(0)-Co(II)	Co(II)-BL(-0)-Co(0)	0.034

12 Molecular Orbitals

12.1 Delocalized (Canonical) Molecular Orbitals (MOs)

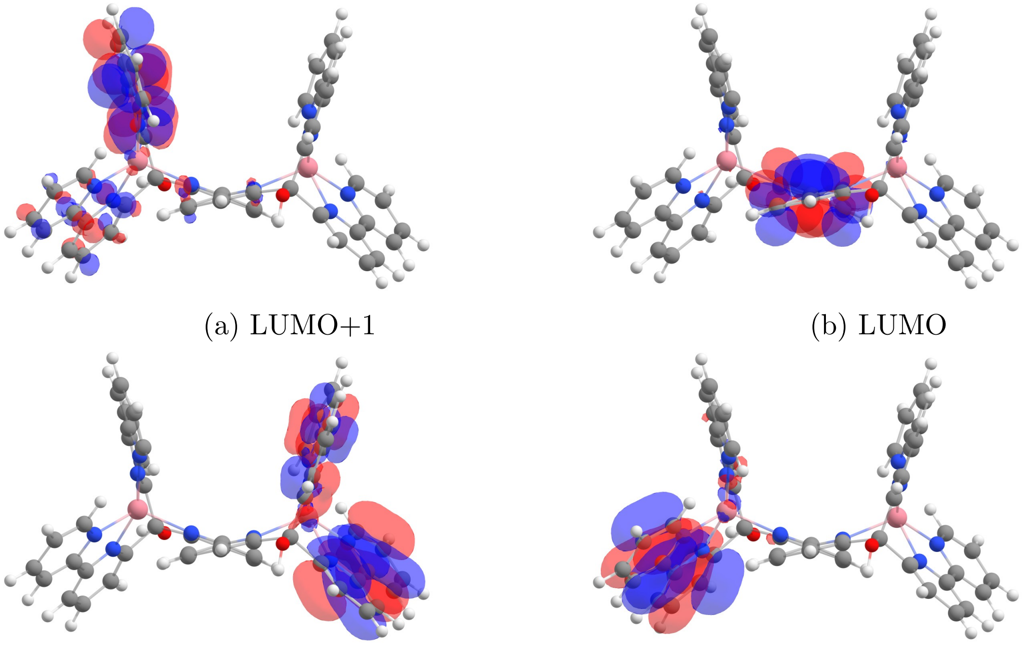

(a) LUMO+1 (b) LUMO (c) HOMO (d) HOMO-1

Figure 6: Delocalized α MOs of non-reduced **C1** structure.

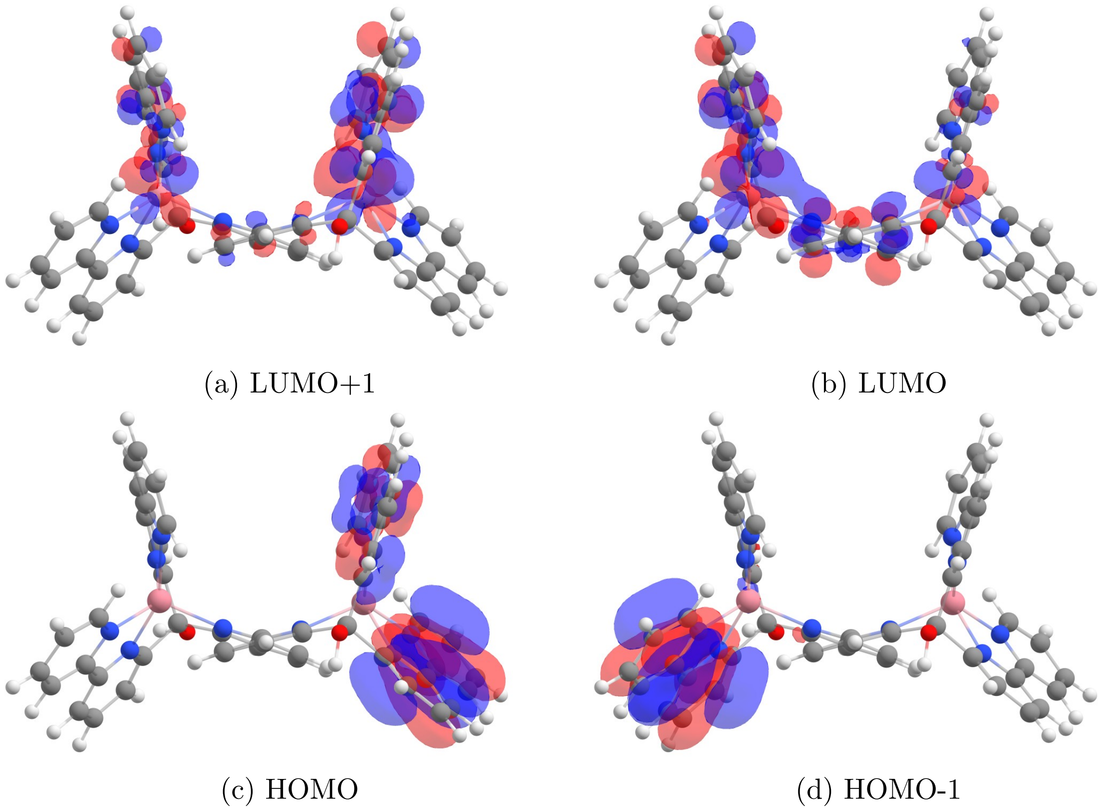

(a) LUMO+1 (b) LUMO

(c) HOMO (d) HOMO-1

Figure 7: Delocalized β MOs of non-reduced **C1** structure.

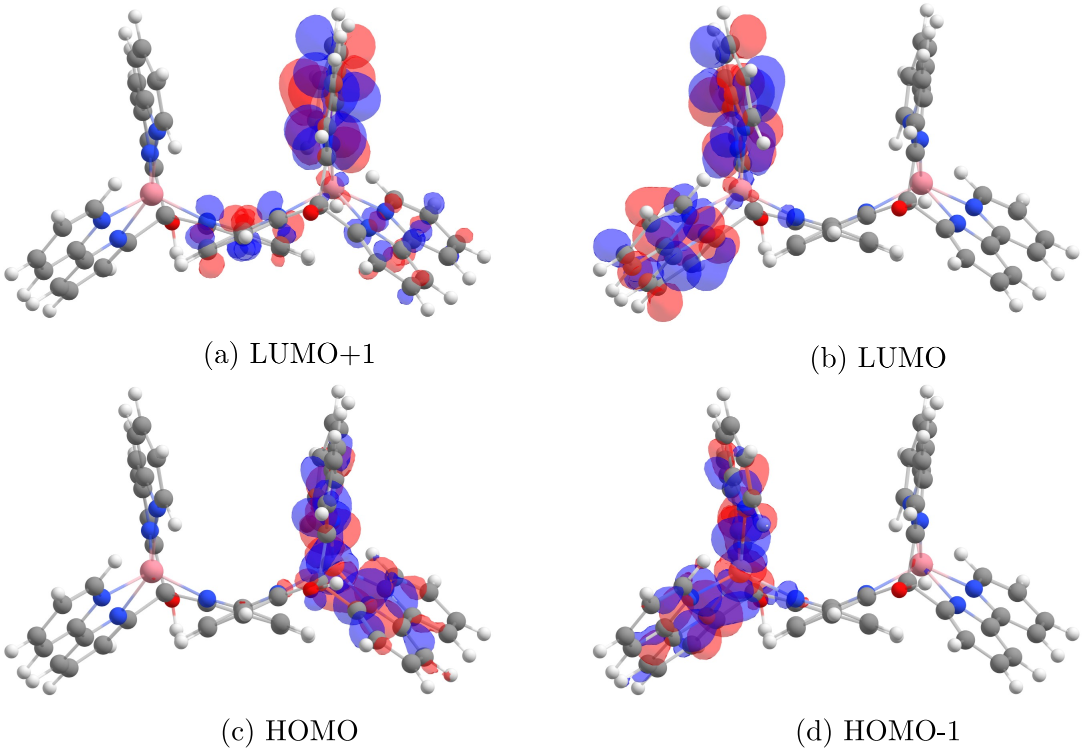

(a) LUMO+1 (b) LUMO

(c) HOMO (d) HOMO-1

Figure 8: Delocalized α MOs of **C1** structure after the first reduction.

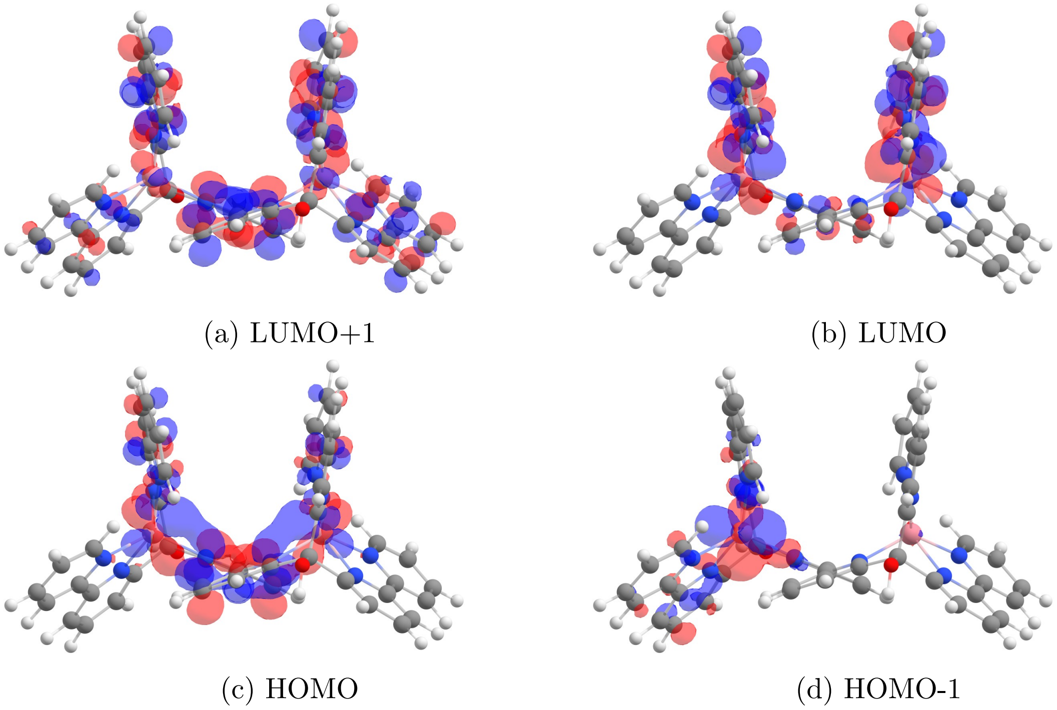

(a) LUMO+1 (b) LUMO

(c) HOMO (d) HOMO-1

Figure 9: Delocalized β MOs of **C1** structure after the first reduction.

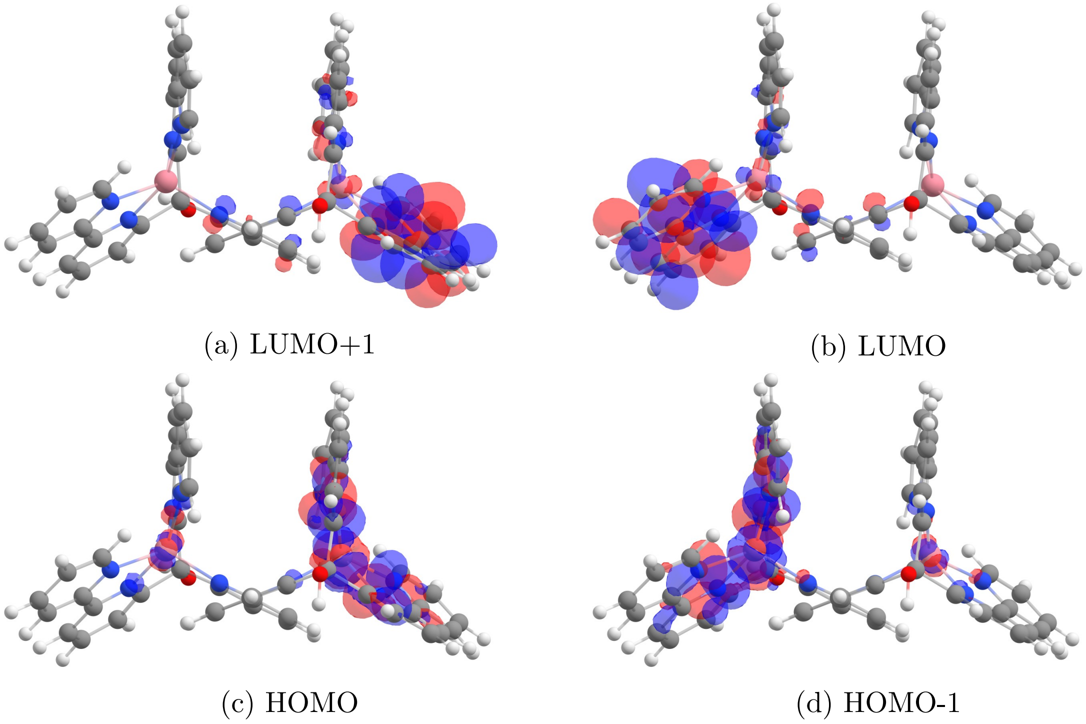

(a) LUMO+1 (b) LUMO

(c) HOMO (d) HOMO-1

Figure 10: Delocalized α MOs of **C1** structure after the second reduction.

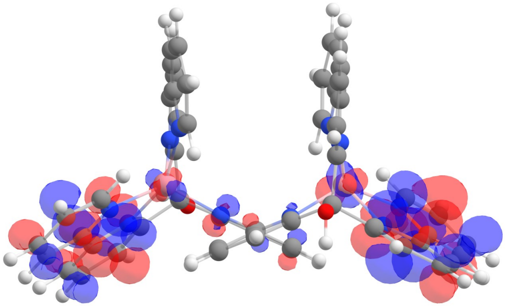

(a) LUMO+1

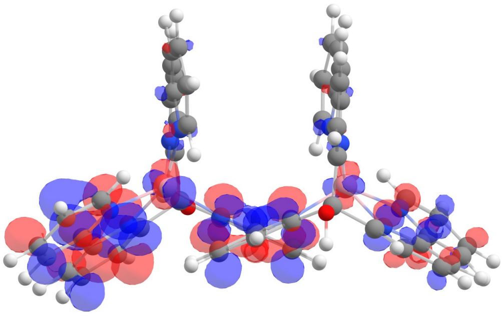

(b) LUMO

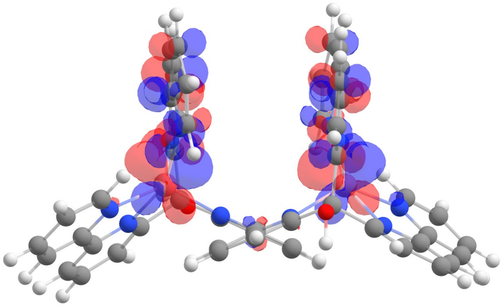

(c) HOMO

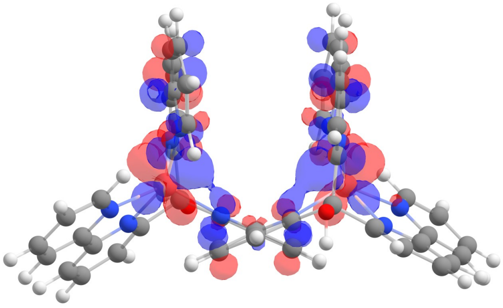

(d) HOMO-1

Figure 11: Delocalized β MOs of **C1** structure after the second reduction.

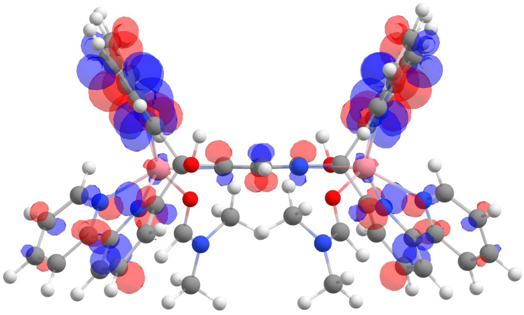

(a) LUMO+1

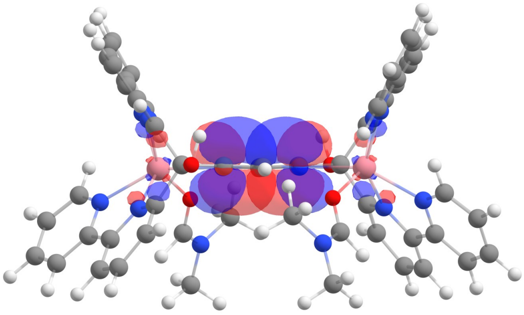

(b) LUMO

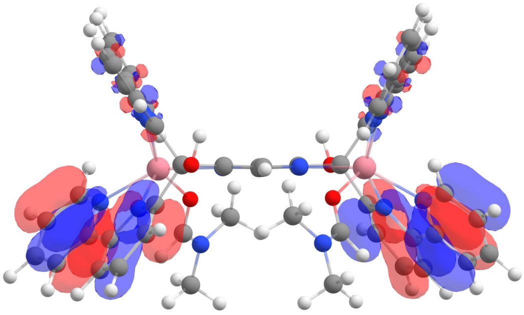

(c) HOMO

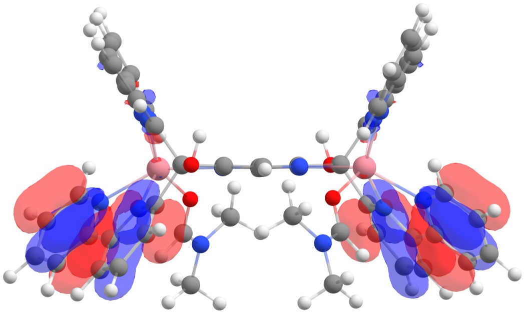

(d) HOMO-1

Figure 12: Delocalized α MOs of non-reduced **C2** structure.

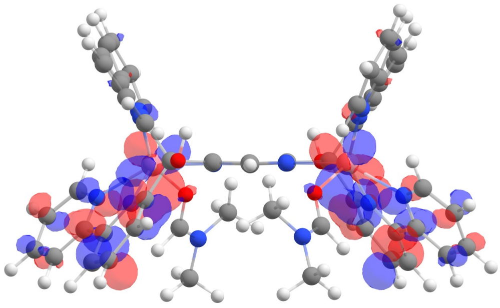

(a) LUMO+1

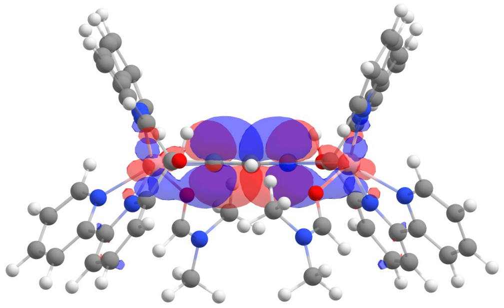

(b) LUMO

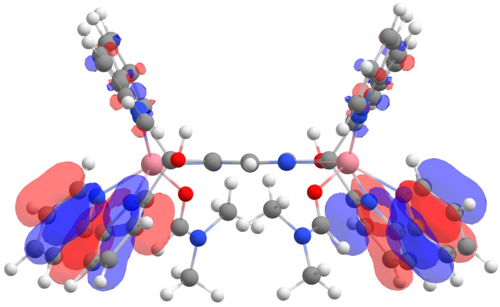

(c) HOMO

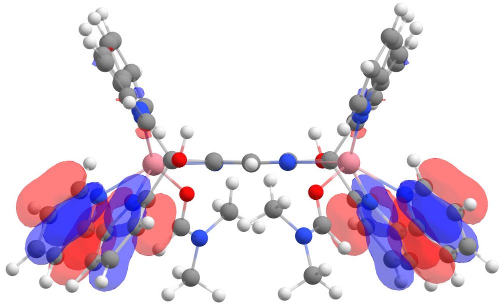

(d) HOMO-1

Figure 13: Delocalized β MOs of non-reduced **C2** structure.

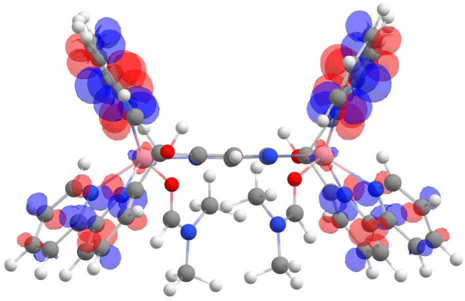

(a) LUMO+1

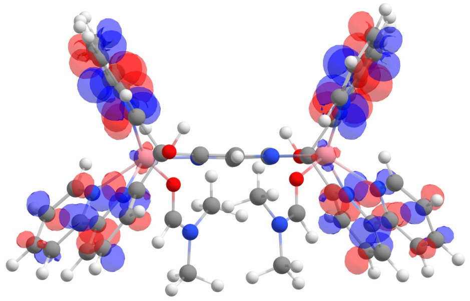

(b) LUMO

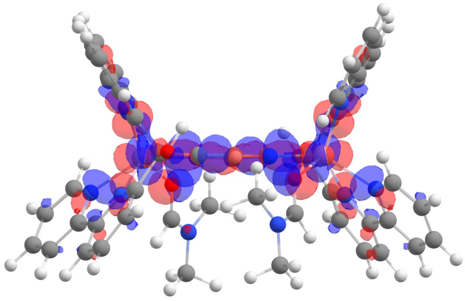

(c) HOMO

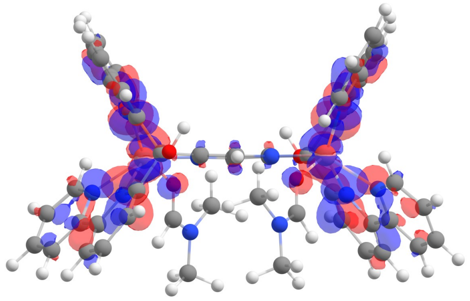

(d) HOMO-1

Figure 14: Delocalized α MOs of **C2** structure after the first reduction.

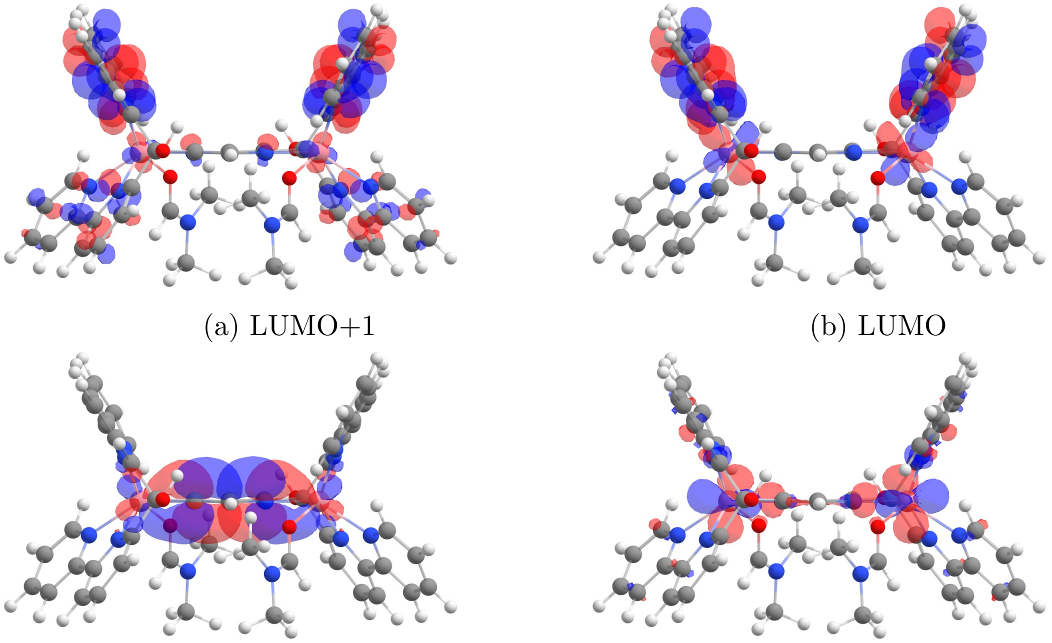

(a) LUMO+1 (b) LUMO

(c) HOMO (d) HOMO-1

Figure 15: Delocalized β MOs of **C2** structure after the first reduction.

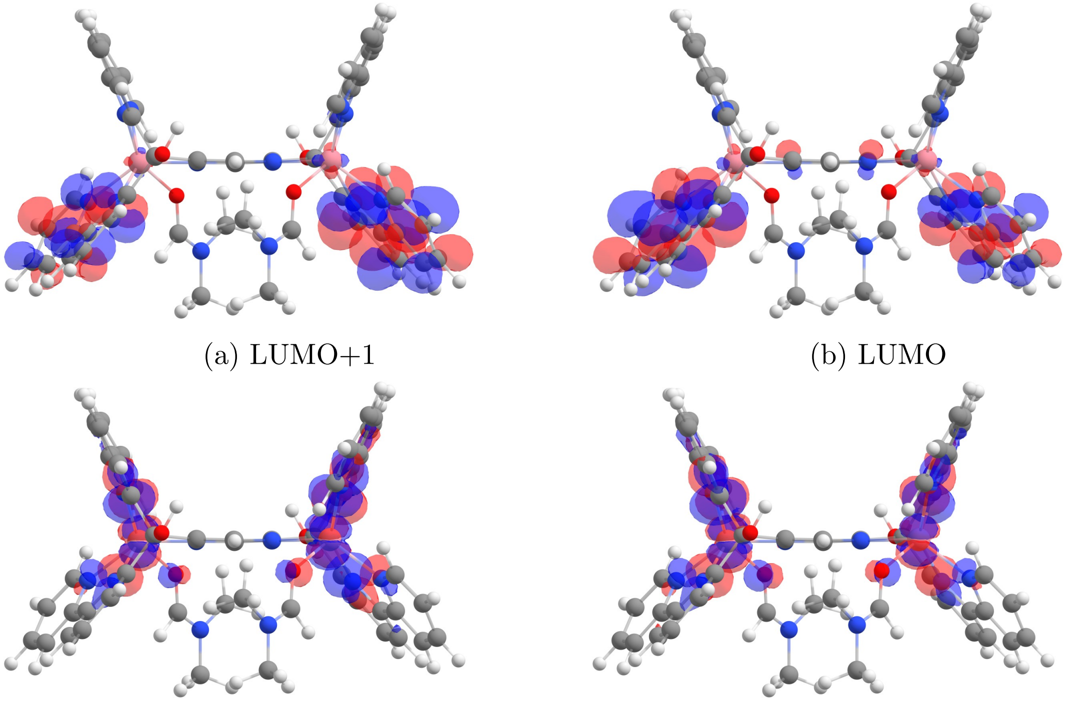

(a) LUMO+1 (b) LUMO

(c) HOMO (d) HOMO-1

Figure 16: Delocalized α MOs of **C2** structure after the second reduction.

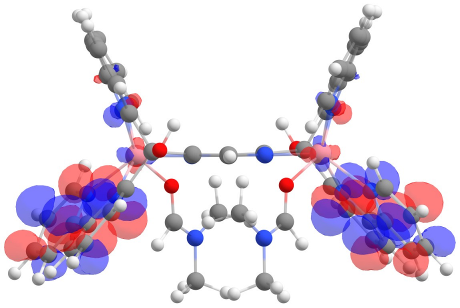

(a) LUMO+1

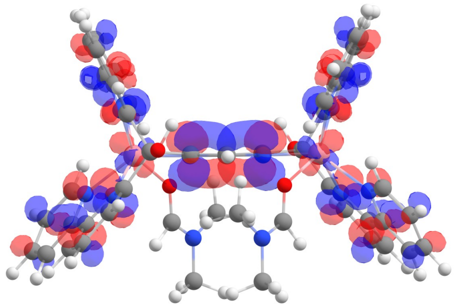

(b) LUMO

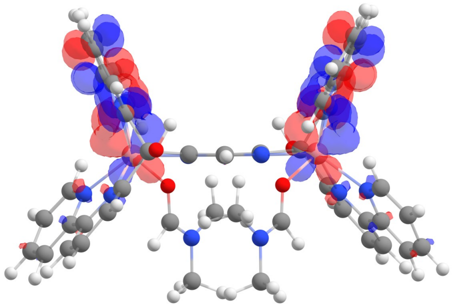

(c) HOMO

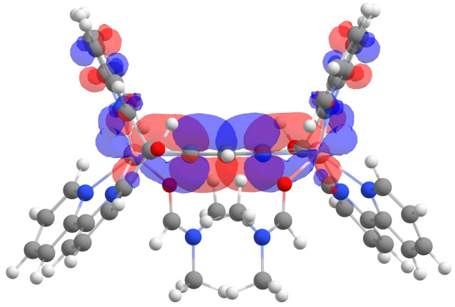

(d) HOMO-1

Figure 17: Delocalized β MOs of **C2** structure after the second reduction.

12.2 Localized Natural Bond Orbitals (NBOs)

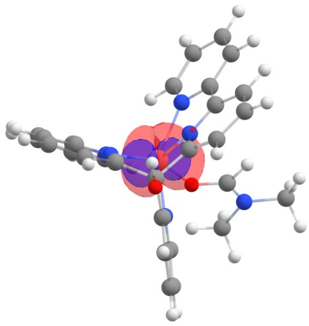

(a) LUMO+1

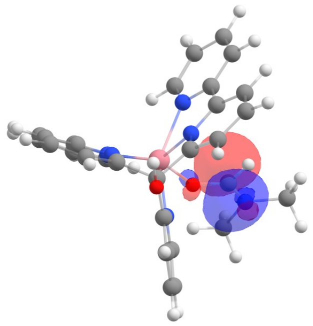

(b) LUMO

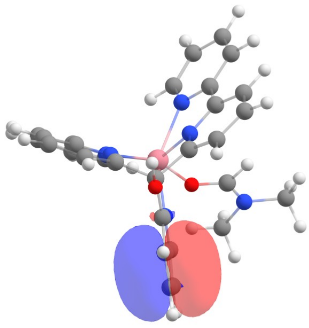

(c) HOMO

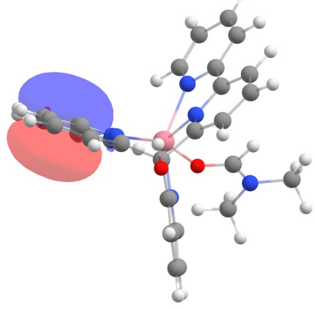

(d) HOMO-1

Figure 18: Localized β NBOs of non-reduced **R1** structure.

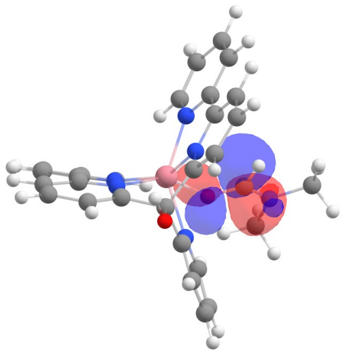

(a) LUMO+1

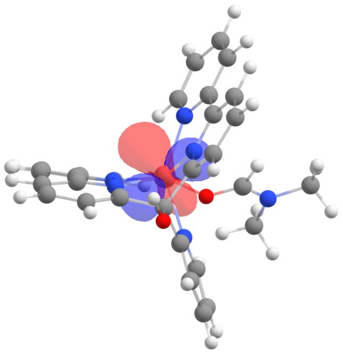

(b) LUMO

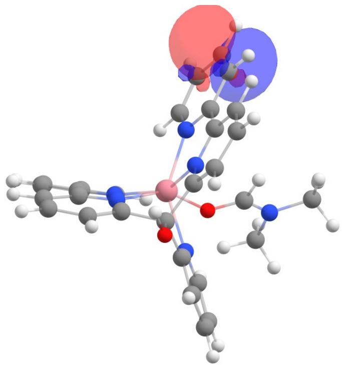

(c) HOMO

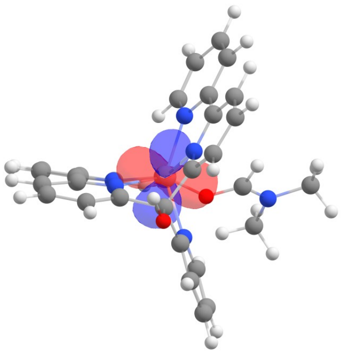

(d) HOMO-1

Figure 19: Localized β NBOs of **R1** structure after the first reduction.

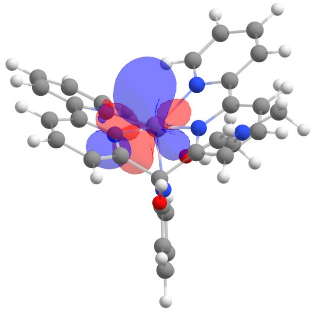

(a) LUMO+1

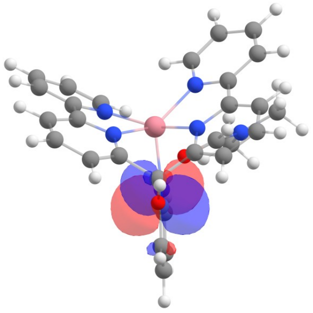

(b) LUMO

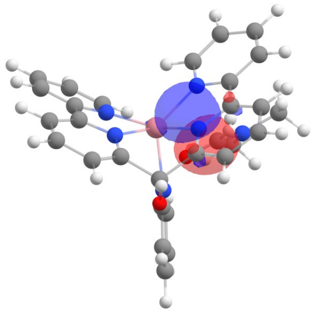

(c) HOMO

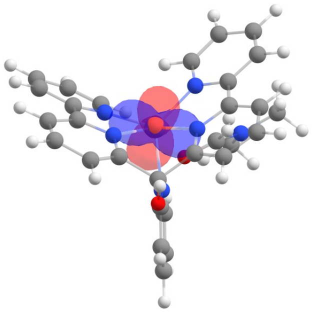

(d) HOMO-1

Figure 20: Localized β NBOs of **R1** structure after the second reduction.

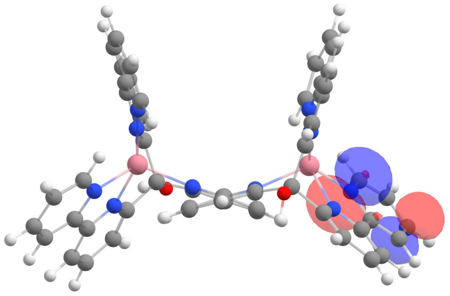

(a) LUMO+1

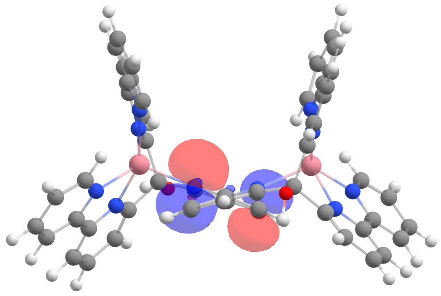

(b) LUMO

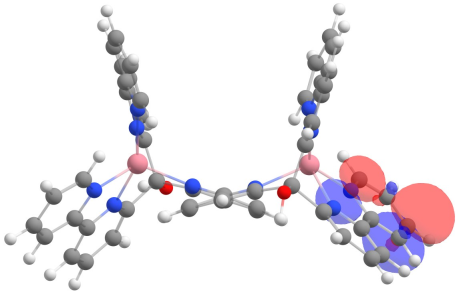

(c) HOMO

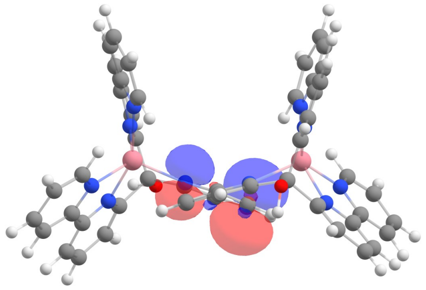

(d) HOMO-1

Figure 21: Localized β NBOs of non-reduced **C1** structure.

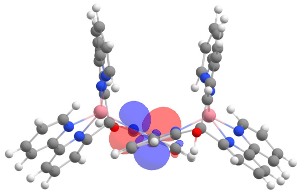

(a) LUMO+2

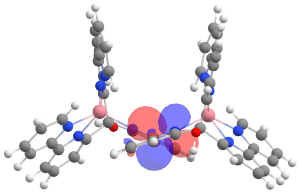

(b) LUMO+1

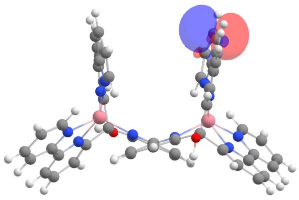

(c) LUMO

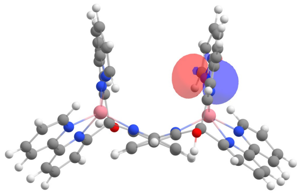

(d) HOMO

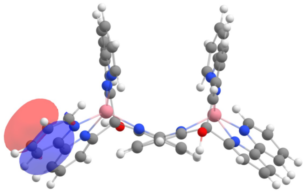

(e) HOMO-1

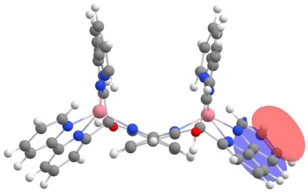

(f) HOMO-2

Figure 22: Localized NBOs of **C1** structure for the first reduction.

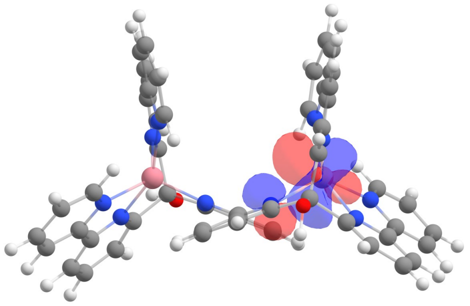

(a) LUMO+1

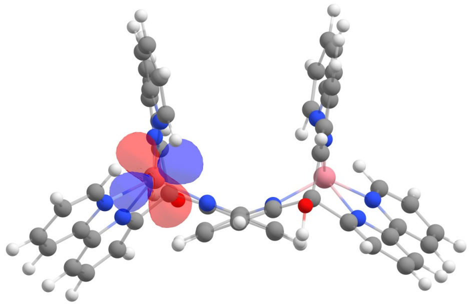

(b) LUMO

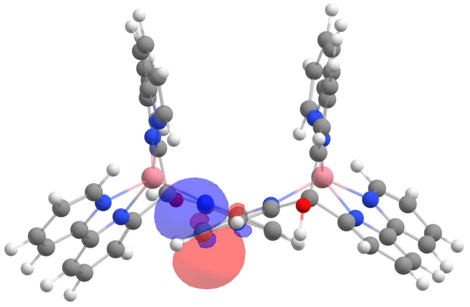

(c) HOMO

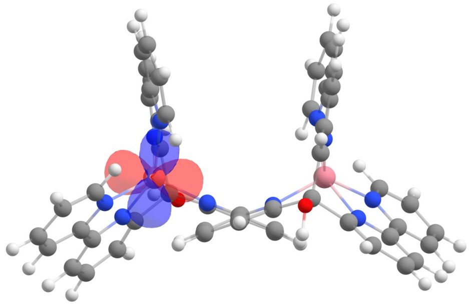

(d) HOMO-1

Figure 23: Localized β NBOs of **C1** structure after the first reduction.

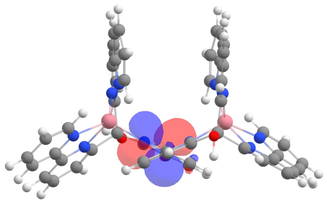

(a) LUMO+2

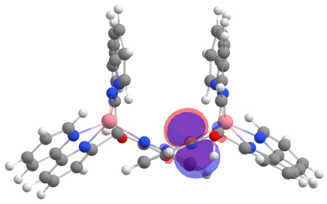

(b) LUMO+1

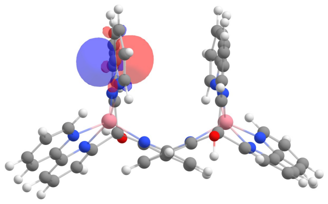

(c) LUMO

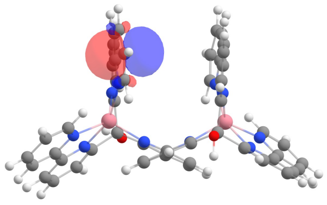

(d) HOMO

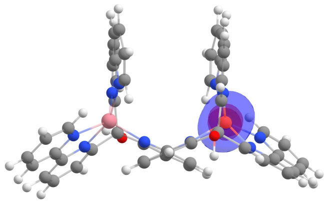

(e) HOMO-1

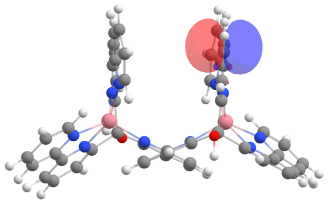

(f) HOMO-2

Figure 24: Localized NBOs of **C1** structure for the second reduction.

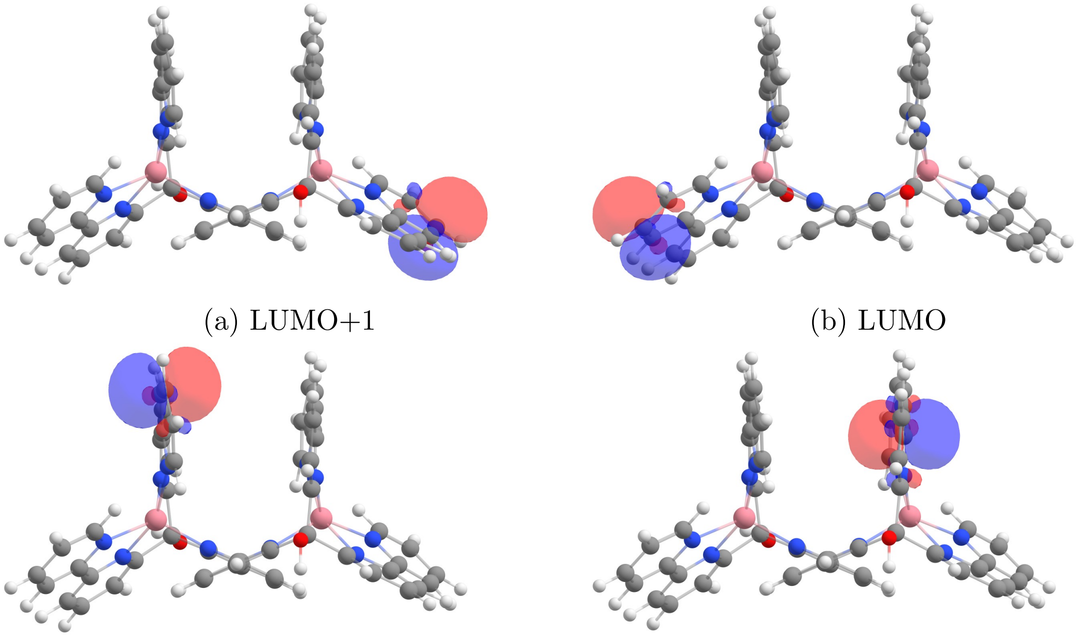

(a) LUMO+1 (b) LUMO

(c) HOMO (d) HOMO-1

Figure 25: Localized β NBOs of **C1** structure after the second reduction.

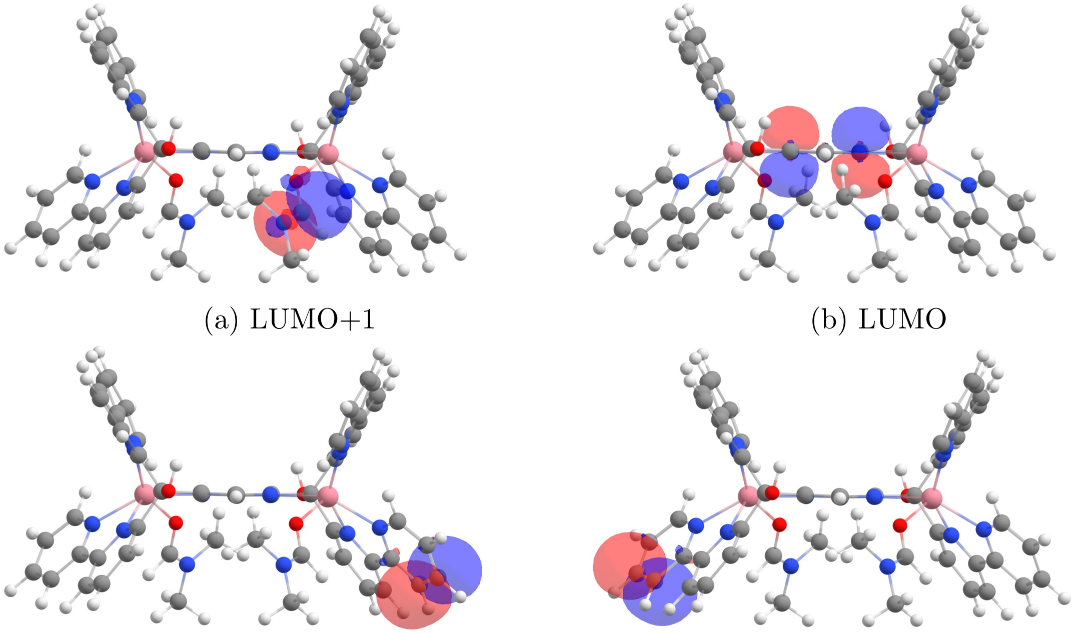

(a) LUMO+1 (b) LUMO

(c) HOMO (d) HOMO-1

Figure 26: Localized β NBOs of non-reduced **C2** structure.

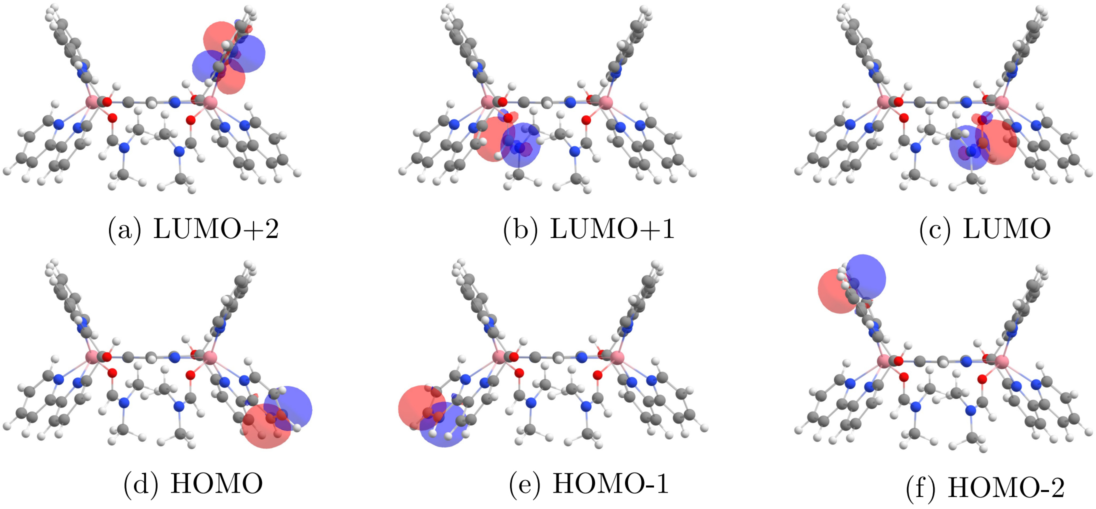

Figure 27: Localized α NBOs of **C2** structure for the first reduction.

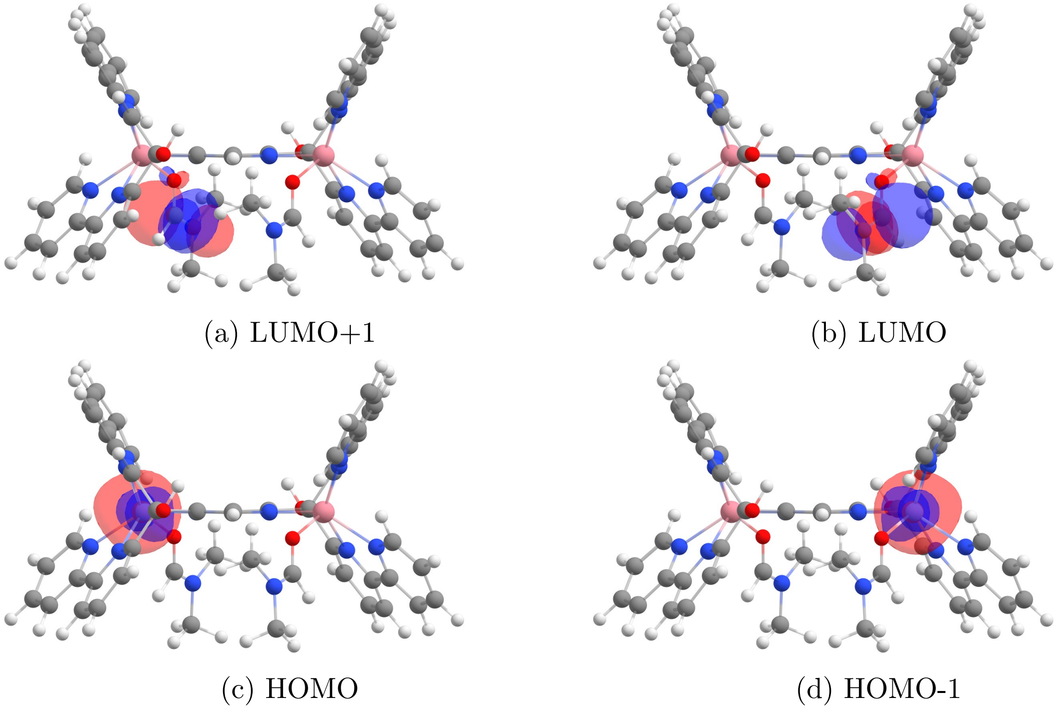

Figure 28: Localized β NBOs of **C2** structure after the first reduction.

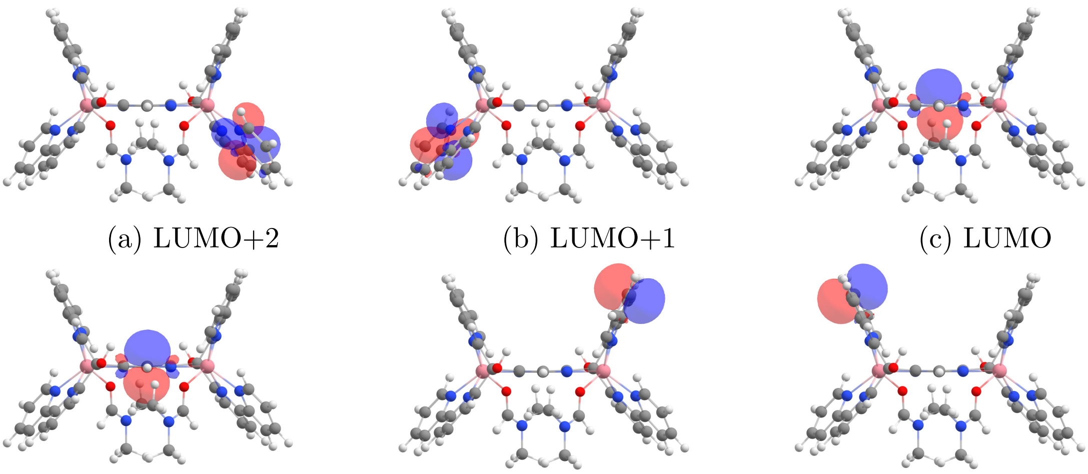

(a) LUMO+2 (b) LUMO+1 (c) LUMO

(d) HOMO (e) HOMO-1 (f) HOMO-2

Figure 29: Localized α NBOs of **C2** structure for the second reduction.

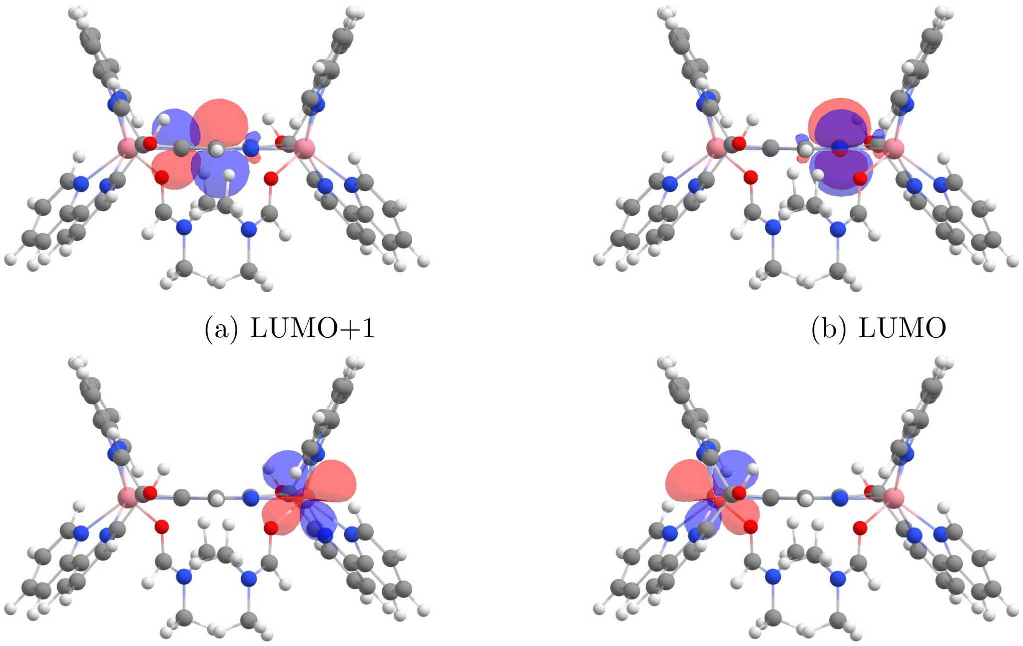

(a) LUMO+1 (b) LUMO

(c) HOMO (d) HOMO-1

Figure 30: Localized β NBOs of **C2** structure after the second reduction.

12.3 Localized Boys Orbitals

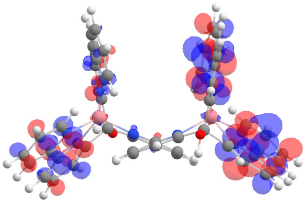

(a) LUMO+3

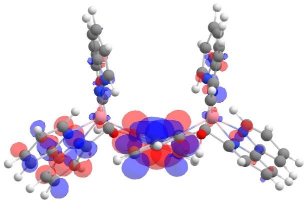

(b) LUMO+2

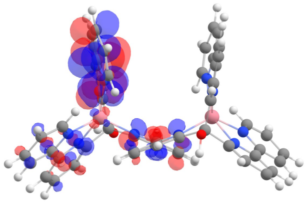

(c) LUMO+1

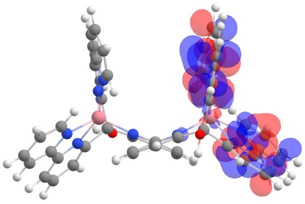

(d) LUMO

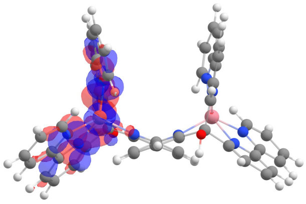

(e) HOMO

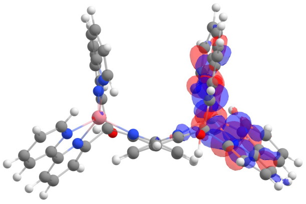

(f) HOMO-1

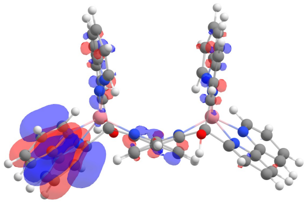

(g) HOMO-2

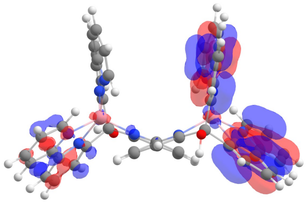

(h) HOMO-3

Figure 31: Localized Boys α orbitals of **C1** structure for the first reduction.

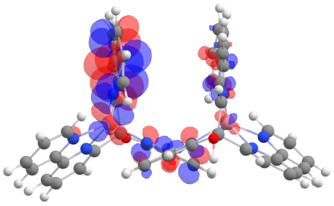

(a) LUMO+3

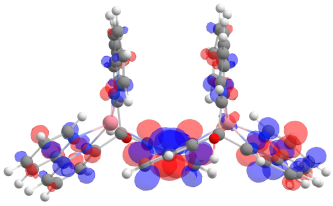

(b) LUMO+2

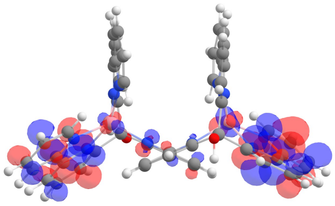

(c) LUMO+1

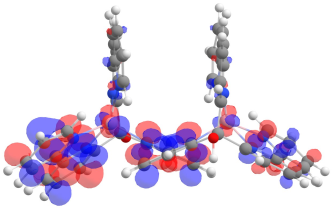

(d) LUMO

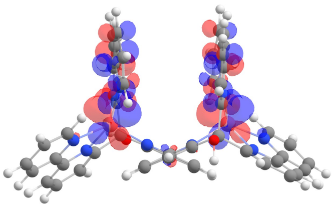

(e) HOMO

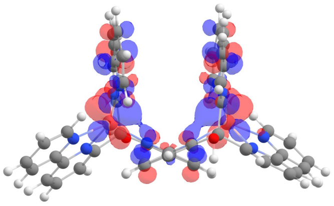

(f) HOMO-1

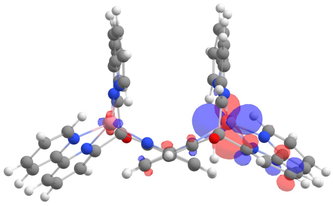

(g) HOMO-2

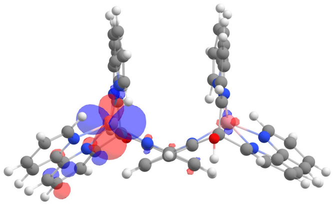

(h) HOMO-3

Figure 32: Localized Boys β orbitals of **C1** structure for the second reduction.

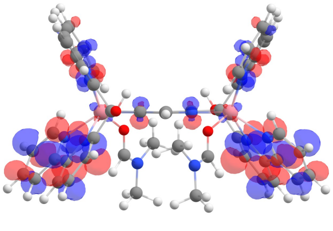

(a) LUMO+3

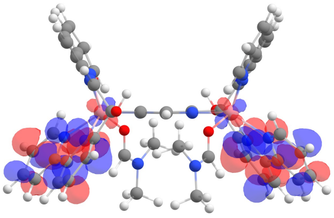

(b) LUMO+2

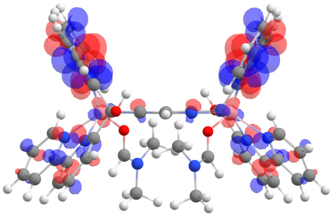

(c) LUMO+1

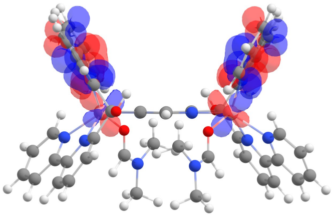

(d) LUMO

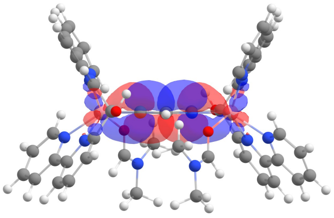

(e) HOMO

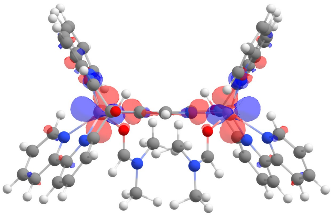

(f) HOMO-1

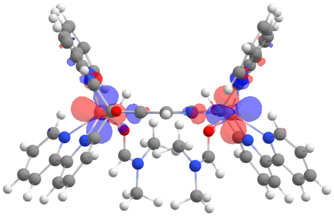

(g) HOMO-2

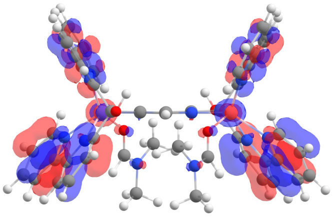

(h) HOMO-3

Figure 33: Localized Boys β orbitals of **C2** structure for the first reduction.

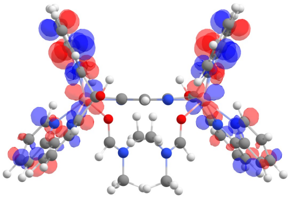

(a) LUMO+3

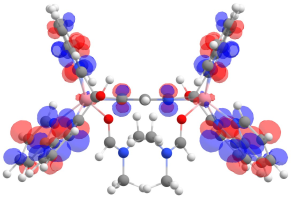

(b) LUMO+2

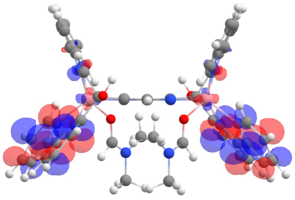

(c) LUMO+1

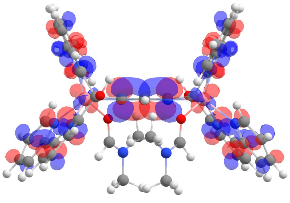

(d) LUMO

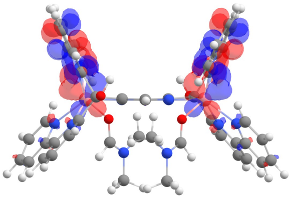

(e) HOMO

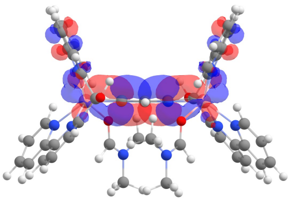

(f) HOMO-1

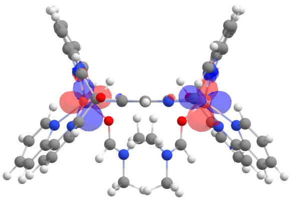

(g) HOMO-2

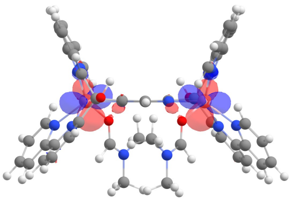

(h) HOMO-3

Figure 34: Localized Boys β orbitals of **C2** structure for the first reduction.

13 References

[1] M. J. Frisch, G. W. Trucks, H. B. Schlegel, G. E. Scuseria, M. A. Robb, J. R. Cheeseman, G. Scalmani, V. Barone, G. A. Petersson, H. Nakatsuji, X. Li, M. Caricato, A. V. Marenich, J. Bloino, B. G. Janesko, R. Gomperts, B. Mennucci, H. P. Hratchian, J. V. Ortiz, A. F. Izmaylov, J. L. Sonnenberg, D. Williams-Young, F. Ding, F. Lipparini, F. Egidi, J. Goings, B. Peng, A. Petrone, T. Henderson, D. Ranasinghe, V. G. Zakrzewski, J. Gao, N. Rega, G. Zheng, W. Liang, M. Hada, M. Ehara, K. Toyota, R. Fukuda, J. Hasegawa, M. Ishida, T. Nakajima, Y. Honda, O. Kitao, H. Nakai, T. Vreven, K. Throssell, J. A. Montgomery, Jr., J. E. Peralta, F. Ogliaro, M. J. Bearpark, J. J. Heyd, E. N. Brothers, K. N. Kudin, V. N. Staroverov, T. A. Keith, R. Kobayashi, J. Normand, K. Raghavachari, A. P. Rendell, J. C. Burant, S. S. Iyengar, J. Tomasi, M. Cossi, J. M. Millam, M. Klene, C. Adamo, R. Cammi, J. W. Ochterski, R. L. Martin, K. Morokuma, O. Farkas, J. B. Foresman, and D. J. Fox. Gaussian 16 Revision C.01, 2016. Gaussian Inc. Wallingford CT.

[2] Lindsay E. Roy, Elena Jakubikova, M. Graham Guthrie, and Enrique R. Batista. Calculation of one-electron redox potentials revisited. is it possible to calculate accurate potentials with density functional methods? *The Journal of Physical Chemistry A*, 113(24):6745–6750, May 2009.

[3] James T. Muckerman and Etsuko Fujita. Theoretical studies of the mechanism of catalytic hydrogen production by a cobaloxime. *Chemical Communications*, 47(46):12456, 2011.

[4] Sergi Grau, Mauro Schilling, Dooshaye Moonshiram, Jordi Benet-Buchholz, Sandra Luber, Antoni Llobet, and Carolina Gimbert-Suriñach. Electrochemically and photochemically induced hydrogen evolution catalysis with co-tetraazamacrocycles occur via different pathways. *ChemSusChem*, 2020.

[5] R. A. Marcus. Relation between charge transfer absorption and fluorescence spectra and the inverted region. *The Journal of Physical Chemistry*, 93(8):3078–3086, April 1989.

[6] Linda Yu Zhang, Richard A. Friesner, and Robert B. Murphy. Ab initio quantum chemical calculation of electron transfer matrix elements for large molecules. *The Journal of Chemical Physics*, 107(2):450–459, July 1997.

[7] E. Aprà, E. J. Bylaska, W. A. de Jong, N. Govind, K. Kowalski, T. P. Straatsma, M. Valiev, H. J. J. van Dam, Y. Alexeev, J. Anchell, V. Anisimov, F. W. Aquino, R. Atta-Fynn, J. Autschbach, N. P. Bauman, J. C. Becca, D. E. Bernholdt, K. Bhaskaran-Nair, S. Bogatko, P. Borowski, J. Boschen, J. Brabec, A. Bruner, E. Cauët, Y. Chen, G. N. Chuev, C. J. Cramer, J. Daily, M. J. O. Deegan, T. H. Dunning, M. Dupuis, K. G. Dyall, G. I. Fann, S. A. Fischer, A. Fonari, H. Früchtl, L. Gagliardi, J. Garza, N. Gawande, S. Ghosh, K. Glaesemann, A. W. Götz, J. Hammond, V. Helms, E. D. Hermes, K. Hirao, S. Hirata, M. Jacquelin, L. Jensen, B. G. Johnson, H. Jónsson, R. A. Kendall, M. Klemm, R. Kobayashi, V. Konkov, S. Krishnamoorthy, M. Krishnan, Z. Lin, R. D. Lins, R. J. Littlefield, A. J. Logsdail, K. Lopata, W. Ma, A. V. Marenich, J. Martin del Campo, D. Mejia-Rodriguez, J. E. Moore, J. M. Mullin, T. Nakajima, D. R. Nascimento, J. A. Nichols, P. J. Nichols, J. Nieplocha, A. Otero de-la Roza, B. Palmer, A. Panyala, T. Pirojsirikul, B. Peng, R. Peverati, J. Pittner, L. Pollack, R. M. Richard, P. Sadayappan, G. C. Schatz, W. A. Shelton, D. W. Silverstein, D. M. A. Smith, T. A. Soares, D. Song, M. Swart, H. L. Taylor, G. S. Thomas, V. Tipparaju, D. G. Truhlar, K. Tsemekhman, T. Van Voorhis, Á. Vázquez-Mayagoitia, P. Verma, O. Villa, A. Vishnu, K. D. Vogiatzis, D. Wang, J. H. Weare, M. J.

Williamson, T. L. Windus, K. Woliński, A. T. Wong, Q. Wu, C. Yang, Q. Yu, M. Zacharias, Z. Zhang, Y. Zhao, and R. J. Harrison. NWChem: Past, present, and future. *The Journal of Chemical Physics*, 152(18):184102, May 2020.

[8] Joseph E Subotnik, Sina Yeganeh, Robert J Cave, and Mark A Ratner. Constructing diabatic states from adiabatic states: Extending generalized mulliken–hush to multiple charge centers with boys localization. *The Journal of chemical physics*, 129(24):244101, 2008.
